# Long-term outcomes after intensive care unit-treated COVID-19, influenza and respiratory sepsis in 2020 – a comparative, population-based cohort study

**DOI:** 10.1007/s15010-025-02644-3

**Published:** 2025-10-03

**Authors:** Franka E. A. Joost, Norman Rose, Aurelia Kimmig, Thomas Ruhnke, Patrik Dröge, Antje Freytag, Christian Günster, Mathias W. Pletz, Martin Roesler, Philipp A. Reuken, Peter Schlattmann, Konrad F. R. Schmidt, Andreas Stallmach, Josephine Storch, Konrad Reinhart, Lisa Wedekind, Carolin Fleischmann-Struzek

**Affiliations:** 1 Institute of Infectious Diseases and Infection Control, Jena University Hospital, Jena, Germany; 2 Center for Sepsis Control and Care, Jena University Hospital, Jena, Germany; 3 AOK Research Institute (WIdO), Berlin, Germany; 4 Institute of General Practice and Family Medicine, Jena University Hospital, Jena, Germany; 5 AOK-Bundesverband, Berlin, Germany; 6 Department of Internal Medicine IV (Gastroenterology, Hepatology, Infectious Diseases), Jena University Hospital, Jena, Germany; 7 Institute of Medical Statistics, Computer and Data Sciences, Jena University Hospital, Jena, Germany; 8 Institute of General Practice and Family Medicine, Charité University Medicine, Berlin, Germany; 9 Institute of General Practice, Faculty of Health Sciences Brandenburg, Brandenburg Medical School Theodor Fontane, Brandenburg, Germany; 10 Sepsis Foundation, Berlin, Germany

**Keywords:** Sepsis, Survivor, Post-Sepsis-Syndrome, Long COVID, Influenza

## Abstract

**Background:**

Sepsis survivors are affected by a broad spectrum of long-term impairments, which overlap with Long-Covid and sequelae after influenza in their clinical presentation. However, we lack comparative assessments on the burden of long-term outcomes, particularly with patients being recruited from the same, contemporary patient population. Therefore we compared long-term outcomes after respiratory sepsis (RS), SARS-CoV-2–associated sepsis (SS) and influenza-associated sepsis (IS).

**Methods:**

Retrospective, population-based cohort study. We included patients > 15 years hospitalized with RS, SS and IS between 01/2020 and 12/2020 in Germany, who received intensive care unit treatment. We compared mortality, readmissions, prevalence of diagnoses in the cognitive, psychological or medical domain, and the number of impaired domains in the 12 months post-discharge between the three survivor cohorts, adjusting for between-group differences in relevant covariates by inverse propensity score weighting based on generalized propensity scores.

**Results:**

Our study included 12,854 patients, of which 8,201 were RS, 3,964 SS and 689 IS survivors. RS survivors had a considerably higher risk for 12-month mortality compared to SS and IS survivors (relative risk, 1.77 [95% CI, 1.54–2.03]; P < 0.001 and relative risk, 1.37 [95% CI, 1.14–1.65]; P = 0.001, respectively). They were more often rehospitalized, affected by multiple domain impairments, cognitive decline and impairments related to the severity of acute disease, e.g. complications of the tracheostoma, compared to survivors after SS and IS. RS survivors had a lower risk for being affected by medical diagnoses compared to SS. Risks for psychological diagnoses did not differ between RS and the other survivor groups.

**Conclusions:**

Although respiratory sepsis survivors seem to be affected by more severe long-term impairments, the overall burden of post-acute sequelae among all survivor groups is high. This warrants efforts to provide targeted aftercare for all survivor populations after life-threatening infections.

**Supplementary Information:**

The online version contains supplementary material available at 10.1007/s15010-025-02644-3.

## Background

Sepsis is a life-threatening disease caused by a dysregulated host response to infection [1], affecting approximately 49 million patients worldwide annually [2]. The immune dysregulation often persists beyond the acute disease and contributes to long-term impairments in survivors, summarized as Post-Sepsis-Syndrome (PSS) [3]. PSS includes cognitive, psychological and medical diagnoses, potentially impeding functional independence for years [4, 5]. In survivors treated in the intensive care unit (ICU), impairments are also referred to as Post-Intensive-Care-Syndrome (PICS) [6].

During the COVID-19 pandemic, it became evident that this viral disease could also lead to long-lasting consequences (Long-Covid (LC)) [7]. There is significant overlap in the clinical presentation of LC and the consequences of sepsis, thus parallels between PSS and LC were discussed early on [8]. Nevertheless, it is still unclear how LC and PSS are pathophysiologically related, and whether they can be considered separate disease entities [9, 10]. A reason for this is the scarcity of comparative long-term studies. The most comprehensive evidence to date comes from a Canadian cohort study which found no difference between COVID-19 and sepsis survivors hospitalized in 2020/2021 with regard to depression and dementia prevalence 12 months post-discharge [11]. However, cardiovascular disease was more common after sepsis than after COVID-19, and sepsis survivors had a higher 12-month mortality [11].

Not only infection with severe acute respiratory syndrome coronavirus type 2 (SARS-CoV-2), but also infection with other bacterial and viral pathogens can lead to long-term health impairments, e.g. infection with influenza viruses or Epstein-Barr virus [12–14]. After influenza infection, post-viral symptoms occurred with lower frequency but persisted similarly as after COVID-19 according to a German retrospective cohort study [15].

Considering these findings, it remains unclear whether the pathogen causing the infection or the severity of infection primarily drives the development of long-term sequelae. Therefore, we compared 12-month outcomes of survivors of ICU-treated (i) SARS-CoV-2–associated sepsis (SS), (ii) influenza-associated sepsis (IS) and (iii) non-SARS-CoV-2-non-influenza pneumonia-associated sepsis (referred to as respiratory sepsis (RS) in the following). As COVID-19 and influenza both lead to respiratory infections, we focused on survivors after respiratory sepsis [12].

## Methods

This study was approved by the Institutional Review Board of the Jena University Hospital, Germany (2023–2992 Daten) as part of the AVENIR study [6]. Reporting follows the STROBE guidelines.

### Study design and data source

A retrospective, population-based cohort study was conducted using nationwide health claims data from 2019 to 2021 of the statutory health insurance “AOK—Die Gesundheitskasse” (AOK), which is the largest German health insurer covering 32% of the German population in 2020 [16, 17]. AOK membership is not tied to any requirements such as health condition, age or regional affiliation. The data included a complete record of the inpatient care, outpatient care and outpatient medication, as reimbursed by the AOK, and survival. Informed consent was waived because all data were deidentified.

### Patient population

**Fig. 1:**
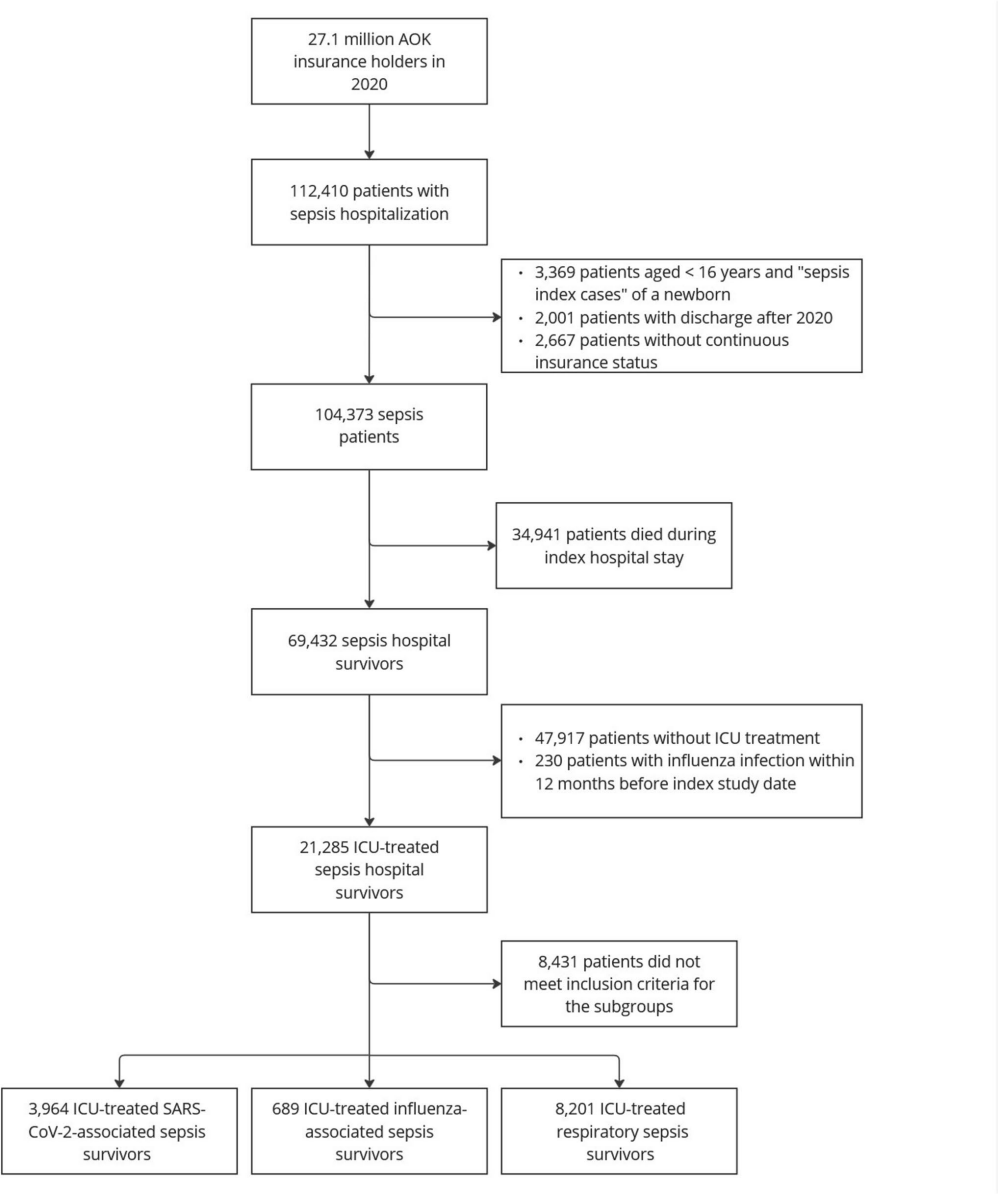
Flowchart of patient inclusion

We identified ICU-treated sepsis patients with hospital discharge in 2020, who survived the hospitalization (Fig. 1). Sepsis was defined by primary or secondary ICD-10 German Modification (ICD-10-GM) hospital discharge codes for sepsis, influenza or COVID-19 combined with ICD-10-GM or procedural codes (*Operationen- und Prozedurenschlüssel* (OPS) codes) for organ dysfunction. The presence of ICU treatment was identified using OPS codes for complex intensive care treatment. For details on codes, see e-Appendix. The first hospitalization with sepsis in 2020 was defined as index stay. Insurance beneficiaries < 16 years of age and without continuous insurance status in the 12 months before and after the study were excluded. By design of the AVENIR study, patients with sepsis 12 months pre-index were excluded. To prevent between-group heterogeneity in terms of previous events in the three groups, we also excluded patients with an influenza infection within 12 months pre-index.

Sepsis patients were divided into three groups (Fig. 1): (i) Patients with SS, (ii) IS, (iii) RS. Group (i) included patients with laboratory-confirmed SARS-CoV-2 infection (ICD-10-GM U07.1!), excluding patients with concurrent influenza infection. Group (ii) included patients with laboratory-confirmed influenza infection (ICD-10-GM J09 or J10), without concurrent SARS-CoV-2 infection. Group (iii) consisted of patients with sepsis with a respiratory focus of infection (for ICD-10-GM codes, see e-Appendix) without SARS-CoV-2 or influenza infection.

### Outcomes

Primary outcomes were: Mortality and the proportion of patients with at least one cognitive, psychological or medical diagnosis. Secondary outcomes included the prevalence of diagnoses in the cognitive, psychological and medical domain, the mean number of impaired domains, the prevalence of specific diagnoses, and the number of rehospitalizations and outpatient treatments. Outpatient treatments were counted if at least one personal or telemedical physician–patient-contact was identified. Specific medical, cognitive and psychological diagnoses are listed in the e-Appendix. All diagnoses were selected based on published literature on LC, PSS, or PICS [18–27] and were reviewed by experts from general medicine, internal medicine, psychiatry, anesthesiology, intensive care medicine, health care service research, post-ICU and post-COVID-19 clinics. A diagnosis was defined as being present if at least one defining ICD-10-GM appeared in hospital discharge data during the 12-month follow-up (primary and secondary discharge diagnoses) or outpatient data (secured diagnoses). For the primary analysis, the prevalence of diagnoses was recorded in the period 0–12 months post-discharge; for a secondary analysis, the period 4–12 months [22, 28] was considered to meet the definition of Post-Covid, defining the occurrence of impairments after three months post-discharge. In this analysis, we included patients who survived three months after hospital discharge.

### Covariates

Potentially confounding covariates were detected during a 12-month look-back pre-index. Covariates were based on findings from literature research [26, 29, 30] and own theory-based considerations. The covariates included age, sex, pre-sepsis nursing care dependency, conditions according to Charlson and Elixhauser comorbidity indices [31], focus of infection, pre-admission vaccination against influenza, the number of pre-sepsis inpatient and outpatient treatments and prior occurrence of all diagnoses considered as outcomes (for definitions, see e-Appendix).

### Statistical analysis

We performed pairwise comparisons between groups: RS vs. SS, RS vs. IS, and IS vs. SS. We used inverse probability of treatment weighting (IPTW) based on generalized propensity scores (GPS) to adjust for differences in covariate distributions across the three groups. GPS are designed for balancing covariate distributions across multiple groups [32, 33]. The individual weights *w*_*ij*_ of an individual in the group *j* are defined as the inverse propensity score *w*_*ij*_ = 1 / *P*_*ij*_(*X*_1_, …, *X*_*M*_), with *P*_*ij*_(*X*_*i*1_, …, *X*_*iM*_) the conditional probability of individual *i* to be in group *j* given the covariates *X*_*i*1_, …, *X*_*iM*_. GPS were estimated by a nonparametric tree-based generalized boosted regression model to account for the large number of covariates and potential higher-order interactions between these covariates [34, 35]. Covariate balancing under IPTW was assessed using absolute standardized mean differences (ASMD) of the weighted sample. ASMD < 0.2 indicate good covariance balancing [34]. Subsequent analyses with the weights require methods for complex samples. Therefore, design corrected Rao-Scott *χ*^2^-Tests were used as well as generalized linear regression models with model-robust sandwich estimators of the standard errors. We report all pairwise adjusted risk differences (RD) and risk ratios (RR) for binary outcomes, and pairwise adjusted unstandardized and standardized mean differences (MD and SMD) for metric outcomes. The SMD is the ratio MD / SD_resiudal_, with SD_resiudal_ the standard deviation of the regression residual of the weighted linear model with the dummy-coded grouping variable. Hence, SD_resiudal_ is an estimator of the pooled within-group standard deviation, and SMD correspond to Cohen’s *d*. RR and SMD serve as effect size measures. All statistical analyses were conducted with R [36]. The R package *twang* was used for GPS estimation. The R package *survey* was used for statistical analyses of the weighted sample [37].

## Results

### Baseline characteristics

Of the 27.1 million AOK insurees in 2020, 112,410 were hospitalized with sepsis. Of these, 21,285 were treated in the ICU and survived hospitalization. Applying the inclusion criteria, the final study population included 3,964 patients with SS, 689 with IS and 8,201 with RS (Fig. 1).

Table 1 and e-Table 3 summarize the characteristics of the unweighted study cohort. Patients with RS were on average older (mean [SD] age, 66.70 [14.10] years vs. mean [SD] age, 63.72 [14.51] years in SS and mean [SD] age, 65.57 [14.22] years in IS) and had a higher unweighted Charlson Comorbidity Index score (mean [SD], 3.21 [2.32] vs. mean [SD], 2.37 [2.18] in SS and mean [SD], 3.01 [2.22] in IS) than patients in the other groups. In addition, patients with RS more frequently had septic shock (26.61% [95% CI, 25.66%-27.57%] vs. 4.26% [95% CI, 3.68%-4.94%] in SS and 8.13% [95% CI, 6.31%-10.41%] in IS) and a longer mean [SD] hospital length of stay of 48.67 [40.28] days (vs. mean [SD], 28.74 [25.62] days in SS and mean [SD], 30.14 [28.14] days in IS). All patients were included in IPTW. The pairwise ASMDs between treatment and control group were substantially reduced by IPTW and were generally below 0.2, for most covariates below 0.1, indicating good covariate balance (see e-Fig. 1 and e-Table 1, for the population of 3-month survivors included in the secondary analysis see e-Fig. 2 and e-Table 2).

**Table 1:** Baseline characteristics of the unweighted cohort in the groups SS, IS and RS

Characteristics of patients	Percentage [95% CI]			
	SARS-CoV-2–associated sepsis (n = 3964)	Influenza-associated sepsis (n = 689)	Respiratory sepsis (n = 8201)	P value^a^
Age, mean (SD)	63.72 (14.51)	65.57 (14.22)	66.70 (14.10)	< 0.001
Female gender	38.12 [36.62–39.64]	42.96 [39.31–46.69]	35.54 [34.52–36.59]	< 0.001
Prior employment	33.15 [31.70–34.63]	23.22 [20.22–26.52]	21.23 [20.36–22.13]	< 0.001
Prior health status				
Dependency on nursing care (care level 1–5)	24.37 [23.07–25.63]	32.80 [29.41–36.14]	39.40 [38.34–40.45]	< 0.001
Inpatient nursing care	4.64 [4.03–5.34]	5.22 [3.80–7.15]	7.27 [6.73–7.85]	< 0.001
Vaccination against influenza	31.61 [30.18–33.07]	28.74 [25.48–32.23]	33.42 [32.41–34.45]	0.011
Charlson Comorbidity Index, mean (SD)	2.37 (2.18)	3.01 (2.22)	3.21 (2.32)	< 0.001
Pre-existing immunosuppression incl. asplenia	22.48 [21.20–23.80]	29.90 [26.60–33.42]	34.76 [33.74–35.80]	< 0.001
Pre-existing long-term mechanical ventilation	1.16 [0.87–1.54]	4.06 [2.83–5.81]	3.19 [2.84–3.60]	< 0.001
Pre-existing dialysis	2.80 [2.33–3.36]	4.35 [3.07–6.15]	3.22 [2.86–3.62]	0.082
Pre-existing immobility	14.08 [13.03–15.19]	19.30 [16.53–22.42]	25.17 [24.24–26.12]	< 0.001
Prior palliative treatment	1.56 [1.22–2.00]	2.03 [1.21–3.38]	3.08 [2.73–3.48]	< 0.001
Characteristics of the index hospital stay				
Septic shock	4.26 [3.68–4.94]	8.13 [6.31–10.41]	26.61 [25.66–27.57]	< 0.001
Hospital length of stay (incl. transfer chains), mean (SD)	28.74 (25.62)	30.14 (28.14)	48.67 (40.28)	< 0.001

Characteristics of patients	Percentage [95% CI]			
	SARS-CoV-2–associated sepsis (n = 3964)	Influenza-associated sepsis (n = 689)	Respiratory sepsis (n = 8201)	*P* value^a^
Organ dysfunction				
Respiratory dysfunction	94.22 [93.45–94.91]	93.32 [91.21–94.96]	90.61 [89.96–91.22]	< 0.001
Coagulopathy	9.11 [8.25–10.04]	12.48 [10.22–15.16]	28.40 [27.43–29.38]	< 0.001
Cardiovascular dysfunction	22.10 [20.83–23.42]	29.75 [26.46–33.27]	49.21 [48.13–50.30]	< 0.001
Hepatic dysfunction	1.11 [0.83–1.49]	1.74 [1.00–3.02]	3.87 [3.47–4.30]	< 0.001
Renal dysfunction	16.40 [15.28–17.58]	25.69 [22.57–29.08]	41.10 [40.04–42.17]	< 0.001
Central nervous system dysfunction	20.59 [19.36–21.87]	29.17 [25.90–32.67]	41.34 [40.27–42.41]	< 0.001
Therapies received during index hospitalization				
Dialysis	6.96 [6.21–7.80]	11.90 [9.69–14.53]	17.85 [17.04–18.70]	< 0.001
Extracorporeal membrane oxygenation (ECMO)	0.98 [0.72–1.34]	1.60 [0.89–2.84]	1.67 [1.41–1.97]	0.012
Mechanical ventilation	57.52 [55.97–59.05]	66.62 [63.01–70.04]	67.66 [66.64–68.67]	< 0.001
Extracorporeal liver replacement therapy	0.05 [0.01–0.18]	0.00 [0.00–0.55]	0.01 [0.00–0.07]	0.342
Surgical treatment	22.12 [20.86–23.44]	30.77 [27.44–34.31]	61.79 [60.73–62.83]	< 0.001
Tracheostomy	10.49 [9.58–11.49]	13.21 [10.88–15.94]	23.22 [22.32–24.14]	< 0.001

*n* = total number; *SD* = standard deviation; *CI* =  confidence interval

^a^*P* value of the *χ*^2^-Test of the Null hypothesis that the three groups do not differ in the distribution of the characteristics (categorical variables), or the *P* value of the *F* Test of the Null hypothesis that the three groups have equal population means (metric variables)

**Fig. 2:**
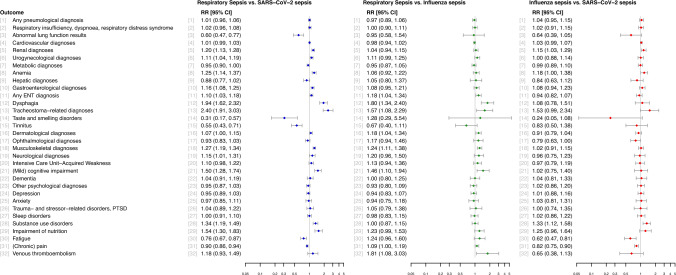
Relative risk of post-acute prevalent diagnoses in RS vs. SS, RS vs. IS and IS vs. SS

**Table 2:** Pairwise RD, MD, RR and SMD of selected outcomes within 0 to 12 months post-discharge

Outcome	Percentage [95% CI]		RD or MD, % [95% CI]	RR [95% CI]	SMD	P* v*alue
Respiratory sepsis vs. SARS-CoV-2–associated sepsis	Respiratory sepsis	SARS-CoV-2–associated sepsis				
Death	26.60 [25.62–27.60]	15.01 [13.13–17.10]	11.59 [9.37–13.80]	1.77 [1.54–2.03]	-	< 0.001
At least one medical, psychological or cognitive diagnosis	94.46 [93.93–94.94]	96.33 [95.30–97.14]	-1.88 [-2.92-(-0.83)]	0.98 [0.97–0.99]	-	0.002
Number of affected domains, mean (SD)	1.89 (0.83)	1.82 (0.77)	0.07 [0.03–0.11]	–	0.08	0.001
Medical diagnosis	94.04 [93.49–94.54]	95.95 [94.91–96.79]	-1,92 [-2,98-(-0,85)]	0.98 [0.97–0.99]	–	0.002
Cognitive diagnosis	33.46 [32.38–34.55]	26.08 [24.10–28.17]	7.37 [5.07–9.68]	1.28 [1.18–1.40]	–	< 0.001
Psychological diagnosis	61.09 [59.93–62.24]	59.78 [57.69–61.84]	1.31 [-1.07–3.69]	1.02 [0.98–1.06]	–	0.280
Number of outpatient treatments, mean (SD)	8.46 (6.02)	9.68 (6.14)	-1.22 [-1.51-(-0.92)]	–	– 0.20	< 0.001
Number of rehospitalizations, mean (SD)	1.74 (2.14)	1.25 (2.03)	0.50 [0.39–0.60]	–	0.23	< 0.001

Respiratory sepsis vs. Influenza-associated sepsis	Respiratory sepsis	Influenza-associated sepsis				
Death	26.60 [25.62–27.60]	19.45 [16.15–23.23]	7.15 [3.48–10.81]	1.37 [1.14–1.65]	–	0.001
At least one medical, psychological or cognitive diagnosis	94.46 [93.93–94.94]	95.23 [92.82–96.86]	-0.78 [-2.81–1.26]	0.99 [0.97–1.01]	–	0.483
Number of affected domains, mean (SD)	1.89 (0.83)	1.86 (0.81)	0.03 [-0.05–0.10]	–	0.03	0.488
Medical diagnosis	94.04 [93.49–94.54]	94.72 [92.28–96.42]	-0,68 [-2.78–1.41]	0.99 [0.97–1.01]	–	0.543
Cognitive diagnosis	33.46 [32.38–34.55]	28.41 [24.81–32.30]	5.05 [1.15–8.95]	1.18 [1.03–1.35]	–	0.015
Psychological diagnosis	61.09 [59.93–62.24]	62.87 [58.69–66.86]	-1.78 [-6.03–2.47]	0.97 [0.91–1.04]	–	0.416
Number of outpatient treatments, mean (SD)	8.46 (6.02)	8.51 (5.69)	-0.04 [-0.54–0.45]	–	– 0.01	0.865
Number of rehospitalizations, mean (SD)	1.74 (2.14)	1.55 (2.19)	0.20 [0.01–0.38]	–	0.09	0.037

Outcome	Percentage [95% CI]		RD or MD, % [95% CI]	RR [95% CI]	SMD	P* v*alue
Influenza-associated sepsis vs. SARS-CoV-2–associated sepsis	Influenza-associated sepsis	SARS-CoV-2–associated sepsis				
Death	19.45 [16.15–23.23]	15.01 [13.13–17.10]	4.44 [0.39–8.49]	1.30 [1.04–1.62]	–	0.025
At least one medical, psychological or cognitive diagnosis	95.23 [92.82–96.86]	96.33 [95.30–97.14]	-1.10 [-3.27–1.07]	0.99 [0.97–1.01]	–	0.288
Number of affected domains, mean (SD)	1.86 (0.81)	1.82 (0.77)	0.04 [-0.04–0.12]	–	0.05	0.299
Medical diagnosis	94.72 [92.28–96.42]	95.95 [94.91–96.79]	-1,23 [-3,46–1,00]	0.99 [0.96–1.01]	–	0.246
Cognitive diagnosis	28.41 [24.81–32.30]	26.08 [24.10–28.17]	2.32 [-1.94–6.59]	1.09 [0.93–1.27]	–	0.279
Psychological diagnosis	62.87 [58.69–66.86]	59.78 [57.69–61.84]	3.09 [-1.50–7.67]	1.05 [0.98–1.13]	–	0.191
Number of outpatient treatments, mean (SD)	8.51 (5.69)	9.68 (6.14)	-1.17 [-1.71-(-0.64)]	–	– 0.20	< 0.001
Number of rehospitalizations, mean (SD)	1.55 (2.19)	1.25 (2.03)	0.30 [0.10–0.50]	–	0.14	0.003

*CI* = confidence interval; *RD *= Risk difference; *MD* = mean difference; *RR* =risk ratio; *SMD* =standardized mean difference

### Prevalence of mortality, health care utilization and impairments

Table 2 shows the outcomes after IPTW

Patients with RS had a higher 1-year mortality rate than patients with SS and IS (RS: 26.60% vs. SS: 15.01%; RR, 1.77 [95% CI, 1.54–2.03]; P < 0.001 and RS: 26.60% vs. IS: 19.45%; RR, 1.37 [95% CI, 1.14–1.65]; P = 0.001). SS survivors were more likely to have symptoms from at least one domain compared to patients with RS, while patients with RS had a higher mean number of domains affected. Such differences were not evident between RS and IS survivors.

RS survivors were more frequently rehospitalized (mean number of rehospitalizations RS: 1.74 vs. SS 1.25; SMD, 0.23; P < 0.001 and RS: 1.74 vs. IS: 1.55; SMD, 0.09; P = 0.037), but had fewer outpatient treatments. They were more likely to experience cognitive sequelae than patients in the other groups (prevalence RS: 33.46% vs. SS: 26.08%; RR, 1.28 [95% CI, 1.18–1.40]; P < 0.001 and RS: 33.46% vs. IS: 28.41%; RR, 1.18 [95% CI, 1.03–1.35]; P = 0.015). However, patients with RS were less likely to have medical sequelae than patients with SS (prevalence RS: 94.04% vs. SS: 95.95%; RR, 0.98; 95% CI, 0.97–0.99; P = 0.002).

Patients with IS did not differ significantly from patients with SS in the type and number of domains affected but were more likely to die during 12-month follow-up (IS: 19.45% vs. SS: 15.01%; RR, 1.30; 95% CI, 1.04–1.62; P = 0.025).

In the secondary analysis (four months to one year, n = 11,396 patients), the results were similar to the overall period. However, patients with RS had more psychological sequelae than patients with SS (prevalence RS: 61.69% vs. SS: 58.29%; RR, 1.06; 95% CI, 1.02–1.10; P = 0.006) (see e-Table 4).

### Prevalent diagnoses within one year

#### Respiratory sepsis vs. SARS-CoV-2–associated sepsis

In RS, a total of 13 diagnoses were more common than in SS (Fig. 2). The three diagnoses with the highest RR were tracheostoma-related diagnoses (prevalence RS: 9.14% vs. SS: 3.80%; RR, 2.40; 95% CI, 1.91–3.03; P < 0.001), dysphagia (RS: 15.11% vs. SS: 7.78%; RR, 1.94; 95% CI, 1.62–2.32; P < 0.001), and impairments of nutrition (RS: 16.43% vs. SS: 10.66%; RR, 1.54; 95% CI, 1.30–1.83; P < 0.001).

Patients with SS were more likely to have taste and smelling disorders (prevalence RS: 0.46% vs. SS: 1.47%; RR, 0.31; 95% CI, 0.17–0.57; P < 0.001), tinnitus (RS: 2.09% vs. SS: 3.77%; RR, 0.55; 95% CI, 0.43–0.71; P < 0.001), and abnormal lung function results (RS: 2.35% vs. SS: 3.90%; RR, 0.60; 95% CI, 0.47–0.77; P < 0.001, Fig. 2), among others.

### Respiratory sepsis vs. influenza-associated sepsis

Patients with RS were more commonly affected by eight diagnoses. The most prominent differences were found in venous thromboembolism (prevalence RS: 4.74% vs. IS: 2.63%; RR, 1.81; 95% CI, 1.08–3.03; P = 0.024), dysphagia (RS: 15.11% vs. IS: 8.41%; RR, 1.80; 95% CI, 1.34–2.40; P < 0.001), and tracheostoma-related diagnoses (RS: 9.14% vs. IS: 5.80%; RR, 1.57; 95% CI, 1.08–2.29; P = 0.016, Fig. 2). For all other conditions, no significant differences were found.

### Influenza-associated sepsis vs. SARS-CoV-2–associated sepsis

Patients with IS more often had substance use disorders (prevalence IS: 25.54% vs. SS: 19.19%; RR, 1.33; 95% CI, 1.12–1.58; P = 0.001), anemia (IS: 27.34% vs. SS: 23.19%; RR, 1.18; 95% CI, 1.00–1.38; P = 0.048) and renal diagnoses (IS: 42.29% vs. SS: 36.74%; RR, 1.15; 95% CI, 1.03–1.29; P = 0.018), but less likely had fatigue (IS: 9.16% vs. SS: 14.87%; RR, 0.62; 95% CI, 0.47–0.81; P < 0.001), ophthalmological diagnoses (IS: 12.32% vs. SS: 15.58%; RR, 0.79; 95% CI, 0.63–1.00; P = 0.043) and (chronic) pain (IS: 48.88% vs. SS: 59.38%; RR, 0.82; 95% CI, 0.75–0.90; P < 0.001, Fig. 2).

## Discussion

In this first population-based study comparing long-term outcomes of ICU-treated RS, SS and IS patients, we found important differences, but also similarities between the patient cohorts after adjustment with IPTW. On the one hand, RS survivors had a considerably higher risk for long-term mortality and hospital readmissions. They were particularly affected by multiple domain impairments, cognitive decline and impairments that may be related to their severe acute disease, such as complications of the tracheostoma. On the other hand, all survivor groups similarly suffered from a large spectrum of post-acute impairments, and with regard to many diagnoses, we could not detect significant between-group differences, particularly not between RS and IS. However, there were some specific long-term impairments that more often affected survivors after SS compared to RS, which may indicate the existence of pathogen-specific sequelae.

Research comparing long-term outcomes of COVID-19 and influenza sepsis is generally scarce [10]. Our results are in line with a previous Canadian study which found an increased 12-month mortality in sepsis compared to COVID-19 survivors [11]. Furthermore, a 2021 review on SARS-CoV-2–associated sepsis concluded a pooled hospital mortality of 33% in ICU-treated COVID-19 [38], which is lower than the estimated mortality of ICU-treated sepsis patients internationally (41.9%) [39]. These differences in acute and long-term mortality may result from variations in initial sepsis severity. In our cohort, RS patients more often developed septic shock and had nearly twice as long hospital length of stays, suggesting more severe organ damage with a higher long-term mortality risk [40].

Existing studies made parly conflicting observations on the burden of post-acute impairments. Comparing ICU-treated sepsis survivors in the Mid-German Sepsis Cohort [41] and COVID-19 survivors treated in an out-patient clinic for post-COVID-19 care, we found higher rates of depression and fatigue among COVID-19 survivors, but comparable rates of cognitive impairments [42]. A Canadian cohort study found similar burdens of dementia, depression and stroke in contemporary sepsis and COVID-19 survivors, while cardiovascular sequelae occurred more frequently in sepsis survivors [11]. Comparing a historical sepsis cohort, all of the above outcomes except stroke occurred at higher rates after sepsis [11]. A 2020–2021 prospective cohort study in Denmark found no differences in cognitive and psychological sequelae between patients with COVID-19, pneumonia and critical illness of other causes [43]. Contrary to this, we detected higher rates of cognitive impairment, but comparable rates of psychological sequelae and cardiovascular diseases. These differences may be explained by differences in design, case definition and severity, as we included only patients after ICU treatment, while e.g. in the Canadian study, only a certain proportion of sepsis and COVID-19 patients were treated in the ICU and received mechanical ventilation [11], thus the disease severity may have been lower and only a part of the population was affected by ICU-specific exposures that may contribute to the development of impairments, such as mechanical ventilation or sedative therapy [44]. Therefore, we hypothesize that the observed pattern of long-term sequelae in our study is to some extent directly related to the disease severity and expression, as sepsis patients in our cohort more frequently had encephalopathy during their index hospitalization, which is a major risk factor for long-term cognitive deficits. RS patients also had longer hospitalizations and received more frequently mechanical ventilation, which increases the risk for sequelae such as dysphagia, tracheostoma-related impairments and musculoskeletal impairments such as critical illness polyneuropathy/myopathy (CIP/CIM) [45, 46]. On the other hand, some differences in long-term outcomes may relate to pathogen-specific effects, such as olfactory dysfunction. A meta-analysis found that approximately one third of patients infected with the SARS-CoV-2 virus continue to have a measurable olfactory dysfunction until one year post-infection [47]. As underlying pathomechanisms, a blockage of odorant transits to the olfactory receptors and damage to the olfactory neuroepithelium, the olfactory bulb and other olfaction-related central nervous system structures are discussed, which may be specifically caused by SARS-CoV-2 [48]. Furthermore, a higher rate of fatigue, chronic pain and abnormal results of lung function testing were found after COVID-19 compared to RS of other origin, confirming previous studies which found higher prevalence of fatigue after COVID-19 than after sepsis [42].

Understanding the specific pattern of sequelae in survivors can be considered a prerequisite for targeted, personalized therapies [10, 49]. Nevertheless, our findings suggest that there is considerable overlap between the post-acute impairments, whose synergistic treatment can also have potential benefits for patients [8, 10]. This applies e.g. to the transferability of aftercare strategies. In this context, the overarching concept of targeting infection-associated chronic conditions (IACC) in aftercare seems useful, which, as a collective term for entities such as LC, PSS or other post-viral/post-bacterial syndromes [50], can combine and strengthen research and therapy approaches in the future. Such novel strategies are urgently needed, as our study confirms the high prevalence of long-term sequelae and post-acute mortality in all survivor populations.

The strengths of our study include the population-based design of the study based on a full health claims record of 27 million insurance holders in Germany. Guided by literature review and expert counseling, we developed a comprehensive definition of sequelae for the use in health claims data.

However, there are important limitations. First, the results relate to the consequences of infection with the SARS-CoV-2 wild type dominant in 2020. They are therefore not generally transferable to other periods of the pandemic and virus variants. Furthermore, in 2020, the influenza season was shorter than usual and less severe [51], potentially impacting the results of our study. Second, health claims data were used to detect long-term outcomes, which necessarily presuppose a utilization of the health care system. This utilization may have been influenced by the restrictions imposed during the pandemic (e.g. hospital capacity restrictions, closure of outpatient practices) and not all diagnoses may be coded validly. For example, misclassification may occur because ventilated patients with viral illnesses often develop bacterial superinfections which routine data probably fail to capture. Such bacterial co-infections could not be excluded in the study and may influence the results. To some extent, the recording of diagnoses also mirror diagnostic habits which might be influenced e.g. by the media and professional attention for LC. Third, we cannot rule out unmeasured confounding. We are also unable to further explore causative mechanisms, thus, preclinical studies should be prioritized to identify underlying pathomechanisms of IACC in general and to determine the impact of the specific pathogen vs. the dysregulated host response or iatrogenic factors.

## Conclusions

While the overall burden of long-term impairments is high, RS survivors seem to be affected particularly by potentially severe long-term impairments compared to SS and IS. This underlines the need for personalized and comprehensive aftercare in survivors after life-threatening infections.

## Supplementary Information

Below is the link to the electronic supplementary material.

Supplementary file1 (DOCX 839 KB)

## Data Availability

The authors confirm that the data utilized in this study cannot be made available in the manuscript, the supplemental files, or in a public repository due to German data protection laws (‘Bundesdatenschutzgesetz’, BDSG). Therefore, they are stored on a secure drive at the WIdO, to facilitate replication of the results. Generally, access to data of statutory health insurance funds for research purposes is possible only under the conditions defined in German Social Law (SGB V § 287). Requests for data access can be sent as a formal proposal specifying the recipient and purpose of the data transfer to the appropriate data protection agency. Access to the data used in this study can only be provided to external parties under the conditions of the cooperation contract of this research project and after written approval by the sickness fund. For assistance in obtaining access to the data, please contact wido@wido.bv.aok.de.

## Abbreviations

AOK: AOK - Die Gesundheitskasse
ASMD: Absolute standardized mean difference
CIP/CIM: Critical illness polyneuropathy/myopathy
GPS: Generalized propensity scores
IACC: Infection-associated chronic condition
ICD-10-GM: ICD-10 German Modification
ICU: Intensive care unit
IPTW: Inverse propensity score weighting
LC: Long-Covid
MD: Mean difference
OPS: Operationen- und Prozedurenschlüssel
PICS: Post-Intensive-Care-Syndrome
PSS: Post-Sepsis-Syndrome
RD: Risk difference
RR: Risk ratio
SARS-CoV-2: Severe acute respiratory syndrome coronavirus type 2
SMD: Standardized mean difference
