# Supplementary material for: Long-term outcomes after intensive care unit-treated COVID-19, influenza and respiratory sepsis in 2020 – a comparative, population-based cohort study
Source: Infection. 2025 Oct 3;54(1):179–89. doi: 10.1007/s15010-025-02644-3 (PMC12864265; doi:10.1007/s15010-025-02644-3)
Supplement: Supplementary file 1 — Supplementary file1 (DOCX 839 KB) [file 15010_2025_2644_MOESM1_ESM.docx]

**Supplemental Online Content**

Long-term outcomes after intensive care unit-treated COVID-19, influenza and respiratory sepsis in 2020 – a comparative, population-based cohort study

[**e-Appendix.** Definitions and Codes 2](#_Toc208926424)

[**e-Figure 1.** Covariate balance checks based on absolute standardized mean differences in sub-sample of N = 12854 sepsis survivors 40](#_Toc208926425)

[**e-Table 1.** Unadjusted (e.g. unweighted) and adjusted (e.g. weighted IPTW) means and absolute standardized mean differences (ASMD) between three groups of N = 12854 sepsis survivors (e.g., influenza-associated sepsis (group 1), SARS-CoV-2–associated sepsis (group 2), and respiratory sepsis (group 3)) 41](#_Toc208926426)

[**e-Figure 2.** Covariate balance checks based on absolute standardized mean differences in sub-sample of N = 11396 sepsis survivors with minimum survival time of 3 months after hospital discharge 52](#_Toc208926427)

[**e-Table 2.** Unadjusted (e.g. unweighted) and adjusted (e.g.weighted IPTW) means and absolute standardized mean differences (ASMD) between three groups of N = 11396 sepsis survivors with minimum survival time of 3 months (e.g., influenza-associated sepsis (group 1), SARS-CoV-2–associated sepsis (group 2), and respiratory Sepsis (group 3)) 53](#_Toc208926428)

[**e-Table 3.** Baseline characteristics of the unweighted cohort in the groups SS, IS and RS 64](#_Toc208926429)

[**e-Table 4.** Pairwise risk differences (RD), mean differences (MD), risk ratios (RR) and standardized mean differences (SMD) of selected outcomes within 3 to 12 months from hospital discharge among N = 11396 3-month survivors 66](#_Toc208926430)

[**e-References** 67](#_Toc208926431)

# **e-Appendix.** Definitions and Codes

**Identification of ICU-treated sepsis survivors**

| Sepsis |  |
| --- | --- |
| ICD-10-GM codes |  |
| R57.2 | Septic shock |
| R65.1 | Systemic Inflammatory Response Syndrome of infectious origin with organ failure |
| A02.1 | Salmonella sepsis |
| A20.0 | Bubonic plague |
| A20.7 | Septicaemic plague |
| A21.7 | Generalized tularaemia |
| A22.7 | Anthrax sepsis |
| A24.1 | Acute or fulminating melioidosis, incl. sepsis |
| A26.7 | Erysipelothrix sepsis |
| A28.2 | Extraintestinal yersiniosis |
| A32.7 | Listerial sepsis |
| A39.1 | Waterhouse-Friderichsen syndrome |
| A39.2 | Acute meningococcal sepsis |
| A39.3 | Chronic meningococcal sepsis |
| A39.4 | Meningococcal sepsis, unspecified |
| A40 | Streptococcal sepsis |
| A41 | Other sepsis |
| A42.7 | Actinomycotic sepsis |
| A48.3 | Toxic shock syndrome |
| A49.9 | Bacterial infection, unspecified |
| A54.8 | Other gonococcal infections, incl. sepsis |
| B00.7 | Disseminated herpesviral disease |
| B37.6 | Candidal endocarditis |
| B37.7 | Candidal sepsis |
| B49 | Unspecified mycosis, incl. fungaemia |
| O75.3 | Other infection during labour, incl. sepsis during labour |
| O85 | Other puerperal infections, incl. sepsis |
| T80.2 | Infections following infusion, transfusion and therapeutic injection, incl. sepsis |
| T81.4 | Infection following a procedure, not elsewhere classified, incl. sepsis |
| T88.0 | Infection following immunization, incl. sepsis after vaccination [immunization] |

| SARS-CoV-2 or influenza infection |  |
| --- | --- |
| ICD-10-GM codes |  |
| J09 | Influenza due to identified zoonotic or pandemic influenza virus |
| J10 | Influenza due to identified seasonal influenza virus |
| U07.1! | Laboratory-confirmed SARS-CoV-2 infection |

| **Organ dysfunction** |  |
| --- | --- |
| Respiratory dysfunction |  |
| ICD-10-GM codes |  |
| J80.0 | Adult respiratory distress syndrome [ARDS] |
| J95.1 | Acute pulmonary insufficiency following thoracic surgery |
| J95.2 | Acute pulmonary insufficiency following nonthoracic surgery |
| J96.0 | Acute respiratory failure, not elsewhere classified |
| J96.9 | Respiratory failure, unspecified |
| R09.2 | Respiratory arrest |
| OPS codes |  |
| 8-701 | Access during mechanical ventilation and using airway clearance techniques: Simple endotracheal intubation |
| 8-704 | Access during mechanical ventilation and using airway clearance techniques: Intubation with double-lumen tube |
| 8-706 | Access during mechanical ventilation and using airway clearance techniques: Mechanical ventilation via face mask |
| 5-311 | Other larynx surgery and surgery on the trachea: Temporary tracheostomy |
| 5-312 | Other larynx surgery and surgery on the trachea: Permanent tracheostomy |

| **Organ dysfunction** |  |
| --- | --- |
| Abnormal coagulation |  |
| ICD-10-GM codes |  |
| D65 | Disseminated intravascular coagulation [defibrination syndrome] |
| D69.57 | Other secondary thrombocytopenia, specified as transfusion-refractory |
| D69.58 | Other secondary thrombocytopenia, not specified as transfusion-refractory |
| D69.59 | Secondary thrombocytopenia, unspecified |
| D69.6 | Thrombozytopenia, unspecified |
| OPS codes |  |
| 8-800.6 | Blood cell transfusion: Whole blood transfusion, red blood cell transfusion and platelet concentrate transfusion: Autologous platelet concentrates |
| 8-800.d | Blood cell transfusion: Whole blood transfusion, red blood cell transfusion and platelet concentrate transfusion: Pathogen inactivated apheresis platelet concentrate |
| 8-800.f | Blood cell transfusion: Whole blood transfusion, red blood cell transfusion and platelet concentrate transfusion: Apheresis platelet concentrate |
| 8-800.g | Blood cell transfusion: Whole blood transfusion, red blood cell transfusion and platelet concentrate transfusion: Platelet concentrate |
| 8-800.h | Blood cell transfusion: Whole blood transfusion, red blood cell transfusion and platelet concentrate transfusion: Pathogen inactivated platelet concentrate |
| 8-800.j | Blood cell transfusion: Whole blood transfusion, red blood cell transfusion and platelet concentrate transfusion: Further pathogen inactivated apheresis platelet concentrates |
| 8-800.k | Blood cell transfusion: Whole blood transfusion, red blood cell transfusion and platelet concentrate transfusion: Further apheresis platelet concentrates |
| 8-800.m | Blood cell transfusion: Whole blood transfusion, red blood cell transfusion and platelet concentrate transfusion: Further platelet concentrates |
| 8-800.n | Blood cell transfusion: Whole blood transfusion, red blood cell transfusion and platelet concentrate transfusion: Further pathogen inactivated platelet concentrates |
| 8-810.j | Transfusion of plasma, plasma components and infusion of volume replacement fluids: Transfusion of plasma components and genetically engineered plasma proteins: Fibrinogen concentrate |
| 8-810.x | Transfusion of plasma, plasma components and infusion of volume replacement fluids: Transfusion of plasma components and genetically engineered plasma proteins: Other |
| 8-812.5 | Transfusion of plasma, plasma components and infusion of volume replacement fluids: Transfusion of plasma and other plasma components and genetically engineered plasma proteins: Prothrombin complex |
| 8-812.6 | Transfusion of plasma, plasma components and infusion of volume replacement fluids: Transfusion of plasma and other plasma components and genetically engineered plasma proteins: Normal plasma |
| 8-812.8 | Transfusion of plasma, plasma components and infusion of volume replacement fluids: Transfusion of plasma and other plasma components and genetically engineered plasma proteins: Pathogen inactivated plasma |

| **Organ dysfunction** |  |
| --- | --- |
| Cardiovascular dysfunction/ shock |  |
| ICD-10 GM codes |  |
| R57.8 | Other shock |
| E86 | Volume depletion |
| I95.9 | Hypotension, unspecified |
| I46.0 | Cardiac arrest |
| I46.9 | Cardiac arrest, unspecified |
| R57.2 | Septic shock |
| OPS codes |  |
| 8-771 | Resuscitation measures: Cardiac or cardiopulmonary resuscitation |
| 8-779 | Resuscitation measures: Other resuscitation measures |
| 8-852 | Extracorporeal circulation and treatment of blood: Extracorporeal gas exchange without and with cardiac assist and pre-ECMO therapy |

| **Organ dysfunction** |  |
| --- | --- |
| Hepatic dysfunction |  |
| ICD-10-GM codes |  |
| K72.0 | Acute and subacute hepatic failure, not elsewhere classified |
| K72.9 | Hepatic failure, unspecified |
| R17 | Hyperbilirubinemia, with or without jaundice, not elsewhere classified |
| OPS code |  |
| 8-858 | Extracorporeal circulation and treatment of blood: Extracorporeal liver support (dialysis) |

| **Organ dysfunction** |  |
| --- | --- |
| Renal dysfunction |  |
| ICD-10-GM codes |  |
| N17.02 | Acute kidney failure with tubular necrosis, stage 2 |
| N17.03 | Acute kidney failure with tubular necrosis, stage 3 |
| N17.12 | Acute kidney failure with acute cortical necrosis, stage 2 |
| N17.13 | Acute kidney failure with acute cortical necrosis, stage 3 |
| N17.22 | Acute kidney failure with medullary necrosis, stage 2 |
| N17.23 | Acute kidney failure with medullary necrosis, stage 3 |
| N17.82 | Other acute kidney failure, stage 2 |
| N17.83 | Other acute kidney failure, stage 3 |
| N17.92 | Acute kidney failure, unspecified, stage 2 |
| N17.93 | Acute kidney failure, unspecified, stage 3 |
| 8-821.2 | Plasmapheresis, immunoadsorption and related procedures: Immunoadsorption and related procedures: Adsorption to remove hydrophobic substances (low and/or moderate molecular weight) |
| 8-853.1 | Extracorporeal circulation and treatment of blood: Haemofiltration: Continuous arteriovenous haemofiltration (CAVH) |
| 8-853.3 | Extracorporeal circulation and treatment of blood: Haemofiltration: Intermittent haemofiltration, anticoagulation with heparin or without anticoagulation |
| 8-853.4 | Extracorporeal circulation and treatment of blood: Haemofiltration: Intermittent haemofiltration, anticoagulation with other substances |
| 8-853.5 | Extracorporeal circulation and treatment of blood: Haemofiltration: Prolonged intermittent haemofiltration, anticoagulation with heparin or without anticoagulation |
| 8-853.6 | Extracorporeal circulation and treatment of blood: Haemofiltration: Prolonged intermittent haemofiltration, anticoagulation with other substances |
| 8-853.7 | Extracorporeal circulation and treatment of blood: Haemofiltration: Pump-assisted continuous venovenous haemofiltration (CVVH), anticoagulation with heparin or without anticoagulation |
| 8-853.8 | Extracorporeal circulation and treatment of blood: Haemofiltration: Pump-assisted continuous venovenous haemofiltration (CVVH), anticoagulation with other substances |
| 8-853.x | Extracorporeal circulation and treatment of blood: Haemofiltration: Other |
| 8-853.y | Extracorporeal circulation and treatment of blood: Haemofiltration: Unspecified |
| 8-854.2 | Extracorporeal circulation and treatment of blood: Haemodialysis: Intermittent haemofiltration, anticoagulation with heparin or without anticoagulation |
| 8-854.3 | Extracorporeal circulation and treatment of blood: Haemodialysis: Intermittent haemofiltration, anticoagulation with other substances |
| 8-854.4 | Extracorporeal circulation and treatment of blood: Haemodialysis: Prolonged intermittent haemofiltration, anticoagulation with heparin or without anticoagulation |
| 8-854.5 | Extracorporeal circulation and treatment of blood: Haemodialysis: Prolonged intermittent haemofiltration, anticoagulation with other substances |
| 8-854.6 | Extracorporeal circulation and treatment of blood: Haemodialysis: Pump-assisted continuous venovenous haemofiltration (CVVHD), anticoagulation with heparin or without anticoagulation |
| 8-854.7 | Extracorporeal circulation and treatment of blood: Haemodialysis: Pump-assisted continuous venovenous haemofiltration (CVVHD), anticoagulation with other substances |
| 8-854.8 | Extracorporeal circulation and treatment of blood: Haemodialysis: Prolonged intermittent haemodialysis to clear protein molecules up to 60,000 |
| 8-854.x | Extracorporeal circulation and treatment of blood: Haemodialysis: Other |
| 8-854.y | Extracorporeal circulation and treatment of blood: Haemodialysis: Unspecified |
| 8-855.1 | Extracorporeal circulation and treatment of blood: Haemodiafiltration: Continuous arteriovenous haemofiltration (CAVHDF) |
| 8-855.3 | Extracorporeal circulation and treatment of blood: Haemodiafiltration: Intermittent haemofiltration, anticoagulation with heparin or without anticoagulation |
| 8-855.4 | Extracorporeal circulation and treatment of blood: Haemodiafiltration: Intermittent haemofiltration, anticoagulation with other substances |
| 8-855.5 | Extracorporeal circulation and treatment of blood: Haemodiafiltration: Prolonged intermittent haemofiltration, anticoagulation with heparin or without anticoagulation |
| 8-855.6 | Extracorporeal circulation and treatment of blood: Haemodiafiltration: Prolonged intermittent haemofiltration, anticoagulation with other substances |
| 8-855.7 | Extracorporeal circulation and treatment of blood: Haemodiafiltration: Pump-assisted continuous venovenous haemofiltration (CVVHDF), anticoagulation with heparin or without anticoagulation |
| 8-855.8 | Extracorporeal circulation and treatment of blood: Haemodiafiltration: Pump-assisted continuous venovenous haemofiltration (CVVHDF), anticoagulation with other substances |
| 8-855.x | Extracorporeal circulation and treatment of blood: Haemodiafiltration: Other |
| 8-855.y | Extracorporeal circulation and treatment of blood: Haemodiafiltration: Unspecified |
| 8-856 | Extracorporeal circulation and treatment of blood: Haemoperfusion |
| 8-85a.0 | Extracorporeal circulation and treatment of blood: Dialysis following no graft function and failure of kidney transplant: Intermittent |
| 8-85a.1 | Extracorporeal circulation and treatment of blood: Dialysis following no graft function and failure of kidney transplant: Continuous |

| **Organ dysfunction** |  |
| --- | --- |
| Encephalopathy |  |
| ICD-10-GM codes |  |
| F05 | Delirium, not induced by alcohol or other psychoactive substances |
| G94.32* | Septic encephalopathy |
| K72.72! | Hepatic encephalopathy grade 2 |
| K72.73! | Hepatic encephalopathy grade 3 |
| K72.74! | Hepatic encephalopathy grade 4 |
| R40 | Somnolence, stupor and coma |
| G93.4 | Encephalopathy, unspecified |

| Complex intensive care treatment |  |
| --- | --- |
| OPS codes |  |
| 8-980 | Other complex multimodal treatment: Complex intensive care (basic procedure) |
| 8-98f | Other complex multimodal treatment: High-scoring complex intensive care (basic procedure) |
| 8-98d | Other complex multimodal treatment: Complex intensive pediatric care (basic procedure) |

**Prior health status**

| Vaccination against influenza |  |
| --- | --- |
| Anatomial Therapeutic Chemical code |  |
| J07BB | Influenza vaccines |
| Fee schedule position |  |
| 89111 | Standard influenza vaccination |
| 89112 | Indication vaccination influenza |

| Pre-existing immunosuppression incl. asplenia |  |
| --- | --- |
| ICD-10-GM codes |  |
| B20 | Human immunodeficiency virus [HIV] disease resulting in infectious and parasitic diseases |
| B21 | Human immunodeficiency virus [HIV] disease resulting in malignant neoplasms |
| B22 | Human immunodeficiency virus [HIV] disease resulting in other specified diseases |
| B23 | Human immunodeficiency virus [HIV] disease resulting in other conditions |
| B24 | Unspecified human immunodeficiency virus [HIV] disease |
| C00 | Malignant neoplasm of lip |
| C01 | Malignant neoplasm of base of tongue |
| C02 | Malignant neoplasm of other and unspecified parts of tongue |
| C03 | Malignant neoplasm of gum |
| C04 | Malignant neoplasm of floor of mouth |
| C05 | Malignant neoplasm of palate |
| C06 | Malignant neoplasm of other and unspecified parts of mouth |
| C07 | Malignant neoplasm of parotid gland |
| C08 | Malignant neoplasm of other and unspecified major salivary glands |
| C09 | Malignant neoplasm of tonsil |
| C10 | Malignant neoplasm of oropharynx |
| C11 | Malignant neoplasm of nasopharynx |
| C12 | Malignant neoplasm of piriform sinus |
| C13 | Malignant neoplasm of hypopharynx |
| C14 | Malignant neoplasm of other and ill-defined sites in the lip, oral cavity and pharynx |
| C15 | Malignant neoplasm of oesophagus |
| C16 | Malignant neoplasm of stomach |
| C17 | Malignant neoplasm of small intestine |
| C18 | Malignant neoplasm of colon |
| C19 | Malignant neoplasm of rectosigmoid junction |
| C20 | Malignant neoplasm of rectum |
| C21 | Malignant neoplasm of anus and anal canal |
| C22 | Malignant neoplasm of liver and intrahepatic bile ducts |
| C23 | Malignant neoplasm of gallbladder |
| C24 | Malignant neoplasm of other and unspecified parts of biliary tract |
| C25 | Malignant neoplasm of pancreas |
| C26 | Malignant neoplasm of other and ill-defined digestive organs |
| C30 | Malignant neoplasm of nasal cavity and middle ear |
| C31 | Malignant neoplasm of accessory sinuses |
| C32 | Malignant neoplasm of larynx |
| C33 | Malignant neoplasm of trachea |
| C34 | Malignant neoplasm of bronchus and lung |
| C37 | Malignant neoplasm of thymus |
| C40 | Malignant neoplasm of bone and articular cartilage of limbs |
| C41 | Malignant neoplasm of bone and articular cartilage of other and unspecified sites |
| C43 | Malignant melanoma and other malignant neoplasms of skin |
| C45 | Mesothelioma |
| C46 | Kaposi sarcoma |
| C47 | Malignant neoplasm of peripheral nerves and autonomic nervous system |
| C48 | Malignant neoplasm of retroperitoneum and peritoneum |
| C49 | Malignant neoplasm of other connective and soft tissue |
| C50 | Malignant neoplasm of breast |
| C51 | Malignant neoplasm of vulva |
| C52 | Malignant neoplasm of vagina |
| C53 | Malignant neoplasm of cervix uteri |
| C54 | Malignant neoplasm of corpus uteri |
| C55 | Malignant neoplasm of uterus, part unspecified |
| C56 | Malignant neoplasm of ovary |
| C57 | Malignant neoplasm of other and unspecified female genital organs |
| C58 | Malignant neoplasm of placenta |
| C60 | Malignant neoplasm of penis |
| C61 | Malignant neoplasm of prostate |
| C62 | Malignant neoplasm of testis |
| C63 | Malignant neoplasm of other and unspecified male genital organs |
| C64 | Malignant neoplasm of kidney, except renal pelvis |
| C65 | Malignant neoplasm of renal pelvis |
| C66 | Malignant neoplasm of ureter |
| C67 | Malignant neoplasm of bladder |
| C68 | Malignant neoplasm of other and unspecified urinary organs |
| C69 | Malignant neoplasm of eye and adnexa |
| C70 | Malignant neoplasm of meninges |
| C71 | Malignant neoplasm of brain |
| C72 | Malignant neoplasm of spinal cord, cranial nerves and other parts of central nervous system |
| C73 | Malignant neoplasm of thyroid gland |
| C74 | Malignant neoplasm of adrenal gland |
| C75 | Malignant neoplasm of other endocrine glands and related structures |
| C76 | Malignant neoplasm of other and ill-defined sites |
| C77 | Secondary and unspecified malignant neoplasm of lymph nodes |
| C78 | Secondary malignant neoplasm of respiratory and digestive organs |
| C79 | Secondary malignant neoplasm of other and unspecified sites |
| C80 | Malignant neoplasm, without specification of site |
| C81 | Hodgkin lymphoma |
| C82 | Follicular lymphoma |
| C83 | Non-follicular lymphoma |
| C84 | Mature T/NK-cell lymphomas |
| C85 | Other and unspecified types of non-Hodgkin lymphoma |
| C88 | Malignant immunoproliferative diseases |
| C90 | Plasmocytoma and malignant plasma cell neoplasms |
| C91 | Lymphoid leukaemia |
| C92 | Myeloid leukaemia |
| C93 | Monocytic leukaemia |
| C94 | Other leukaemias of specified cell type |
| C95 | Leukaemia of unspecified cell type |
| C96 | Other and unspecified malignant neoplasms of lymphoid, haematopoietic and related tissue |
| C97 | Malignant neoplasms of independent (primary) multiple sites |
| D45 | Polycythaemia vera |
| D46 | Myelodysplastic syndromes |
| D47.1 | Chronic myeloproliferative disease |
| D47.3 | Essential (haemorrhagic) thrombocythaemia |
| D47.4 | Osteomyelofibrosis |
| D47.5 | Chronic eosinophilic leukaemia [hypereosinophilic syndrome] |
| D47.7 | Other specified neoplasms of uncertain or unknown behaviour of lymphoid, haematopoietic and related tissue |
| D47.9 | Neoplasm of uncertain or unknown behaviour of lymphoid, haematopoietic and related tissue, unspecified |
| D56 | Thalassaemia |
| D57 | Sickle-cell disorders |
| D58 | Other hereditary haemolytic anaemias |
| D59 | Acquired haemolytic anaemia |
| D60 | Acquired pure red cell aplasia [erythroblastopenia] |
| D61 | Other aplastic anaemias |
| D63* | Anaemia in chronic diseases classified elsewhere |
| D65 | Disseminated intravascular coagulation [defibrination syndrome] |
| D66 | Hereditary factor VIII deficiency |
| D67 | Hereditary factor IX deficiency |
| D68 | Other coagulation defects |
| D69 | Purpura and other haemorrhagic conditions |
| D70 | Agranulocytosis and neutropenia |
| D71 | Functional disorders of polymorphonuclear neutrophils |
| D72 | Other disorders of white blood cells |
| D73.0 | Hyposplenism |
| D73.1 | Hypersplenism |
| D73.2 | Chronic congestive splenomegaly |
| D73.5 | Infarction of spleen |
| D73.8 | Other diseases of spleen |
| D73.9 | Disease of spleen, unspecified |
| D74 | Methaemoglobinaemia |
| D75 | Other diseases of blood and blood-forming organs |
| D76 | Other specified diseases with participation of lymphoreticular and reticulohistiocytic tissue |
| D77 | Other disorders of blood and blood-forming organs in diseases classified elsewhere |
| D80 | Immunodeficiency with predominantly antibody defects |
| D81 | Combined immunodeficiencies |
| D82 | Immunodeficiency associated with other major defects |
| D83 | Common variable immunodeficiency |
| D84 | Other immunodeficiencies |
| D89 | Other disorders involving the immune mechanism, not elsewhere classified |
| D90 | Immunocompromise due to radiotherapy, chemotherapy and other immunosuppressive therapies |
| Q89.0 | Congenital malformations of spleen |
| U60 | Clinical categories of HIV disease |
| U61 | T-helper cells count in HIV disease |
| Z08 | Follow-up examination after treatment for malignant neoplasms |
| Z21 | Asymptomatic human immunodeficiency virus [HIV] infection status |
| Z51.0 | Radiotherapy session |
| Z51.1 | Chemotherapy session for neoplasm |
| Z51.2 | Other chemotherapy |
| Z94 | Transplanted organ and tissue status |

| Pre-existing medical immunosuppression |  |
| --- | --- |
| Anatomial Therapeutic Chemical codes |  |
| L01 | Antineoplastic agents |
| L01A | Alkylating agents |
| L01B | Antimetabolites |
| L01C | Plant alkaloids and other natural products |
| L01D | Cytotoxic antibiotics and related substances |
| L01E | Protein kinase inhibitors |
| L01F | Monoclonal antibodies and antibody drug conjugates |
| L01X | Other antineoplastic agents |
| L04A | Immunosuppressants |
| L04AA | Selective immunosuppressants |
| L04AB | Tumor necrosis factor alpha (TNF-α) inhibitors |
| L04AC | Interleukin inhibitors |
| L04AD | Calcineurin inhibitors |
| L04AX | Other immunosuppressants |
| H02AB | Glucocorticoids |
| H02BX | Corticosteroids for systemic use, combinations |

| Pre-existing asplenia |  |
| --- | --- |
| ICD-10-GM codes |  |
| D73.0 | Hyposplenism |
| Q89.0 | Asplenia (congenital) |
| OPS code |  |
| 5-4131 | Operations on spleen and bone marrow: Splenectomy: Total |

| Prior organ transplantation |  |
| --- | --- |
| ICD-10-GM code |  |
| Z94 | Transplanted organ and tissue status |
| OPS codes |  |
| 5-504 | Operations on the liver: Liver transplantation |
| 5-375 | Rhythm surgery and other operations on heart and pericardium: Heart and heart-lung transplantation |
| 5-555 | Operations on the kidney: Kidney transplantation |
| 5-335 | Other operations on lungs and bronchus: Lung transplantation |
| 5-5281 | Operations on the pancreas: Pancreatic (tissue) transplantation: Transplantation of a pancreatic segment |
| 5-5282 | Operations on the pancreas: Pancreatic (tissue) transplantation: Transplantation of pancreas (entire organ) |
| 5-4676 | Other operations on small and large intestine: Other intestinal reconstruction: Small intestine transplantation |

| Pre-existing infection/colonisation with multi-resistant pathogens |  |
| --- | --- |
| ICD-10-GM codes |  |
| U80 | Gram-positive bacteria with specified antibiotic resistance, requiring special therapeutic or hygienic measures |
| U81 | Gram-negative bacteria with specified antibiotic resistance, requiring special therapeutic or hygienic measures |
| U82 | Mycobacteria with resistance against TB drugs (first line) |
| U83 | Candida with resistance against Fluconazole and Voriconazole |
| U84 | Herpes virus with restistance against antivirals |
| OPS code |  |
| 8-987 | Complex treatment in the case of colonisation or infection with multidrug resistant pathogens [MDR] |

| Previously implanted foreign material |  |
| --- | --- |
| ICD-10-GM codes |  |
| Z95 | Presence of cardiac and vascular implants and grafts |
| Z96.4 | Presence of endocrine implants |
| Z96.5 | Presence of tooth-root and mandibular implants |
| Z96.6 | Presence of orthopedic joint implants |
| Z96.7 | Presence of other bone and tendon implants |
| Z98.2 | Presence of cerebrospinal fluid drainage device |
| Z45 | Encounter for adjustment and management of implanted device |
| Z99.4 | Dependence on artificial heart |

| Pre-existing mechanical ventilation |  |
| --- | --- |
| OPS codes |  |
| 8-713 | Mechanical ventilation and respiratory assistance via face mask or tube and weaning from mechanical ventilation: Mechanical ventilation and respiratory assistance in adults |
| 8-718 | Mechanical ventilation and respiratory assistance via face mask or tube and weaning from mechanical ventilation: Weaning from mechanical ventilation |
| 8-716.01 | Mechanical ventilation and respiratory assistance via face mask or tube and weaning from mechanical ventilation: Home mechanical ventilation setup: Initial setup: Invasive home ventilation following unsuccessful weaning from mechanical ventilation |
| 8-716.02 | Mechanical ventilation and respiratory assistance via face mask or tube and weaning from mechanical ventilation: Home mechanical ventilation setup: Initial setup: Invasive home ventilation as elective measure or with no attempt at weaning from mechanical ventilation |
| 8-701 | Access during mechanical ventilation and using airway clearance techniques: Simple endotracheal intubation |
| 8-704 | Access during mechanical ventilation and using airway clearance techniques: Intubation with double-lumen tube |
| 8-706 | Access during mechanical ventilation and using airway clearance techniques: Mechanical ventilation via face mask |
| 5-311 | Other larynx surgery and surgery on the trachea: Temporary tracheostomy |
| 5-312 | Other larynx surgery and surgery on the trachea: Permanent tracheostomy |

| Pre-existing long-term mechanical ventilation |  |
| --- | --- |
| ICD-10-GM codes |  |
| Z99.0 | Dependence on aspirator |
| Z99.1 | Dependence on respirator |
| OPS codes |  |
| 8-716.01 | Mechanical ventilation and respiratory assistance via face mask or tube and weaning from mechanical ventilation: Home mechanical ventilation setup: Initial setup: Invasive home ventilation following unsuccessful weaning from mechanical ventilation |
| 8-716.02 | Mechanical ventilation and respiratory assistance via face mask or tube and weaning from mechanical ventilation: Home mechanical ventilation setup: Initial setup: Invasive home ventilation as elective measure or with no attempt at weaning from mechanical ventilation |

| Pre-existing dialysis |  |
| --- | --- |
| ICD-10-GM codes |  |
| Z99.2 | Dependence on renal dialysis |
| Z49 | Care involving dialysis |
| OPS codes |  |
| 8-821.2 | Plasmapheresis, immunoadsorption and related procedures: Immunoadsorption and related procedures: Adsorption to remove hydrophobic substances (low and/or moderate molecular weight) |
| 8-853.1 | Extracorporeal circulation and treatment of blood: Haemofiltration: Continuous arteriovenous haemofiltration (CAVH) |
| 8-853.3 | Extracorporeal circulation and treatment of blood: Haemofiltration: Intermittent haemofiltration, anticoagulation with heparin or without anticoagulation |
| 8-853.4 | Extracorporeal circulation and treatment of blood: Haemofiltration: Intermittent haemofiltration, anticoagulation with other substances |
| 8-853.5 | Extracorporeal circulation and treatment of blood: Haemofiltration: Prolonged intermittent haemofiltration, anticoagulation with heparin or without anticoagulation |
| 8-853.6 | Extracorporeal circulation and treatment of blood: Haemofiltration: Prolonged intermittent haemofiltration, anticoagulation with other substances |
| 8-853.7 | Extracorporeal circulation and treatment of blood: Haemofiltration: Pump-assisted continuous venovenous haemofiltration (CVVH), anticoagulation with heparin or without anticoagulation |
| 8-853.8 | Extracorporeal circulation and treatment of blood: Haemofiltration: Pump-assisted continuous venovenous haemofiltration (CVVH), anticoagulation with other substances |
| 8-853.x | Extracorporeal circulation and treatment of blood: Haemofiltration: Other |
| 8-853.y | Extracorporeal circulation and treatment of blood: Haemofiltration: Unspecified |
| 8-854.2 | Extracorporeal circulation and treatment of blood: Haemodialysis: Intermittent haemofiltration, anticoagulation with heparin or without anticoagulation |
| 8-854.3 | Extracorporeal circulation and treatment of blood: Haemodialysis: Intermittent haemofiltration, anticoagulation with other substances |
| 8-854.4 | Extracorporeal circulation and treatment of blood: Haemodialysis: Prolonged intermittent haemofiltration, anticoagulation with heparin or without anticoagulation |
| 8-854.5 | Extracorporeal circulation and treatment of blood: Haemodialysis: Prolonged intermittent haemofiltration, anticoagulation with other substances |
| 8-854.6 | Extracorporeal circulation and treatment of blood: Haemodialysis: Pump-assisted continuous venovenous haemofiltration (CVVHD), anticoagulation with heparin or without anticoagulation |
| 8-854.7 | Extracorporeal circulation and treatment of blood: Haemodialysis: Pump-assisted continuous venovenous haemofiltration (CVVHD), anticoagulation with other substances |
| 8-854.8 | Extracorporeal circulation and treatment of blood: Haemodialysis: Prolonged intermittent haemodialysis to clear protein molecules up to 60,000 |
| 8-854.x | Extracorporeal circulation and treatment of blood: Haemodialysis: Other |
| 8-854.y | Extracorporeal circulation and treatment of blood: Haemodialysis: Unspecified |
| 8-855.1 | Extracorporeal circulation and treatment of blood: Haemodiafiltration: Continuous arteriovenous haemofiltration (CAVHDF) |
| 8-855.3 | Extracorporeal circulation and treatment of blood: Haemodiafiltration: Intermittent haemofiltration, anticoagulation with heparin or without anticoagulation |
| 8-855.4 | Extracorporeal circulation and treatment of blood: Haemodiafiltration: Intermittent haemofiltration, anticoagulation with other substances |
| 8-855.5 | Extracorporeal circulation and treatment of blood: Haemodiafiltration: Prolonged intermittent haemofiltration, anticoagulation with heparin or without anticoagulation |
| 8-855.6 | Extracorporeal circulation and treatment of blood: Haemodiafiltration: Prolonged intermittent haemofiltration, anticoagulation with other substances |
| 8-855.7 | Extracorporeal circulation and treatment of blood: Haemodiafiltration: Pump-assisted continuous venovenous haemofiltration (CVVHDF), anticoagulation with heparin or without anticoagulation |
| 8-855.8 | Extracorporeal circulation and treatment of blood: Haemodiafiltration: Pump-assisted continuous venovenous haemofiltration (CVVHDF), anticoagulation with other substances |
| 8-855.x | Extracorporeal circulation and treatment of blood: Haemodiafiltration: Other |
| 8-855.y | Extracorporeal circulation and treatment of blood: Haemodiafiltration: Unspecified |
| 8-856 | Extracorporeal circulation and treatment of blood: Haemoperfusion |
| 8-85a.0 | Extracorporeal circulation and treatment of blood: Dialysis following no graft function and failure of kidney transplant: Intermittent |
| 8-85a.1 | Extracorporeal circulation and treatment of blood: Dialysis following no graft function and failure of kidney transplant: Continuous |
| Fee schedule position |  |
| 13602 | Flat rate supplementary fee for continuous care of a patient requiring dialysis |
| 13610 | Flat rate supplementary fee for medical care in the case of haemodialysis, peritoneal dialysis and special procedures |
| 13611 | Flat rate supplementary fee for medical care in the case of peritoneal dialysis |
| 04562 | Flat rate supplementary fee for continuous care of a patient requiring dialysis |
| 04564 | Flat rate supplementary fee for paediatric nephrology care when carrying out haemodialysis |
| 04565 | Flat rate supplementary fee for paediatric nephrology care when carrying out peritoneal dialysis |
| 40815 | Flat rate fee for dialysis in patients up to the age of 18 years at their place of residence |
| 40816 | Flat rate fee for peritoneal dialysis in patients up to the age of 18 years |
| 40817 | Flat rate fee for peritoneal dialysis in patients up to the age of 18 years at their place of residence |
| 40818 | Flat rate fee for haemodialysis in patients up to the age of 18 years during a holiday or other absence |
| 40819 | Flat rate fee for peritoneal dialysis in patients up to the age of 18 years during a holiday or other absence |
| 40823 | Flat rate fee for dialysis in insured persons from the age of 18 years |
| 40824 | Flat rate fee for dialysis in insured persons from the age of 18 years at their place of residence |
| 40825 | Flat rate fee for peritoneal dialysis in insured persons from the age of 18 years |
| 40826 | Flat rate fee for peritoneal dialysis in insured persons from the age of 18 years at their place of residence |
| 40827 | Flat rate fee for intermittent peritoneal dialysis in insured persons from the age of 18 years at their place of residence |
| 40828 | Flat rate fee for dialysis from the age of 18 years during a holiday or workrelated stay |
| 40829 | Supplement to flat rate fee 40823 or 40825 for insured persons aged 59-69 years |
| 40830 | Supplement to flat rate fee 40824, 40826 and 40827 for insured persons aged 59-69 years |
| 40831 | Supplement to flat rate fee 40823 or 40825 for insured persons aged 69-79 years |
| 40832 | Supplement to flat rate fee 40824, 40826 and 40827 for insured persons aged 69-79 years |
| 40833 | Supplement to flat rate fee 40823 or 40825 for insured persons from 79 years of age |
| 40834 | Supplement to flat rate fee 40824, 40826 and 40827 for insured persons from 79 years of age |
| 40835 | Supplement to flat rate fee 40816, 40823 or 40825 for dialysis in a patient with an infection |
| 40836 | Supplement to flat rate fee 40815, 40817, 40818, 40819, 40824, 40826 to 40828 for dialysis in a patient with an infection |
| 40837 | Supplement to flat rate fee 40816 or 40825 for intermittent peritoneal dialysis |
| 40838 | Supplement to flat rate fee 40817, 40819, 40827 or 40828 for intermittent peritoneal dialysis |

| Pre-existing immobility |  |
| --- | --- |
| ICD-10-GM codes |  |
| R26.2 | Difficulty in walking, not elsewhere classified |
| R26.3 | Immobility |
| R29.6 | Tendency to fall, not elsewhere classified |
| Z99.3 | Dependence on wheelchair |
| Z74.0 | Need for assistance due to reduced mobility |

| Prior palliative treatment |  |
| --- | --- |
| ICD-10-GM codes |  |
| Z51.5 | Palliative care |
| OPS codes |  |
| 8-982 | Palliative medical complex treatment |
| 8-98e | Specialized inpatient palliative medical complex treatment |
| 8-98h | Specialized palliative medical complex treatment through a palliative care service |
| Fee schedule position |  |
| 01425 | Initial prescription for specialized outpatient palliative care |
| 01426 | Follow-up prescription for the continuation of specialized outpatient palliative care |
| 03370 | Initial palliative medical assessment of the patient's status including treatment plan |
| 03371 | Supplement to the insured person's flat rate fee 03000 for palliative medical care of the patient at the doctor's office |
| 03372 | Supplement to flat rate fee 01410 or 01413 for palliative medical care in the patient's home |
| 03373 | Supplement to flat rate fee 01411, 01412 or 01415 for palliative medical care in the patients' home |
| 04370 | Initial palliative medical assessment of the patient's status including treatment plan |
| 04371 | Supplement to the insured person's flat rate fee 04000 for palliative medical care of the patient at the doctor's office |
| 04372 | Supplement to flat rate fee 01410 or 01413 for palliative medical care in the patient's home |
| 04373 | Supplement to flat rate fee 01411, 01412 or 01415 for palliative medical care in the patient's home |
| 37300 | Initial palliative medical assessment of the patient's status including treatment plan |
| 37302 | Supplement to the insured or basic flat rate fee or to GOP 25210, 25211 or 25214 for the coordinating contract doctor |
| 37305 | Supplement to flat rate fee to GOPs 01410 and 01413 for palliative medical care in the patient's home |
| 37306 | Supplement flat rate fee to GOPs 01411, 01412 and 01415 for palliative medical care in the patient's home |
| 37314 | Consultation Doctor with additional qualification in palliative medicine |
| 37317 | Supplement to flat rate fee to GOP 37302 for availability and attendance during critical phases |
| 37318 | Telephone advice |
| 37320 | Case conference |

**Patient characteristics and characteristics of the index hospital stay**

| Septic shock |  |
| --- | --- |
| ICD-10-GM code |  |
| R57.2 | Septic shock |

| **Focus of infection** |  |
| --- | --- |
| Upper respiratory tract |  |
| ICD-10-GM codes |  |
| A36.0 | Pharyngeal diphtheria |
| A36.1 | Nasopharyngeal diphtheria |
| A36.2 | Laryngeal diphtheria |
| B87.3 | Nasopharyngeal myiasis |
| J00 | Acute nasopharyngitis [common cold] |
| J01 | Acute sinusitis |
| J02 | Acute pharyngitis |
| J04 | Acute laryngitis and tracheitis |
| J05 | Acute obstructive laryngitis [croup] and epiglottitis |
| J06 | Acute upper respiratory infections of multiple and unspecified sites |
| J31 | Chronic rhinitis, nasopharyngitis and pharyngitis |
| J32 | Chronic sinusitis |
| J37 | Chronic laryngitis and laryngotracheitis |
| J38.3 | Other diseases of vocal cords |
| J38.7 | Other diseases of larynx |

| **Focus of infection** |  |
| --- | --- |
| Lower respiratory tract |  |
| ICD-10-GM codes |  |
| J18.0 | Bronchopneumonia, unspecified organism |
| A06.5 | Amebic lung abscess |
| Lower respiratory tract, spec. bronchi |  |
| ICD-10-GM codes |  |
| J20 | Acute bronchitis |
| J21 | Acute bronchiolitis |
| J40 | Bronchitis, not specified as acute or chronic |
| J41 | Simple and mucopurulent chronic bronchitis |
| J42 | Unspecified chronic bronchitis |
| J44.0 | Chronic obstructive pulmonary disease with (acute) lower respiratory infection |
| Lower respiratory tract, spec. lung |  |
| A15.0 | Tuberculosis of lung, confirmed by microscopic examination of the sputum, with or without detection by culture or molecular biological methods |
| A15.1 | Tuberculosis of lung, only confirmed by culture |
| A15.2 | Pulmonary tuberculosis, histologically confirmed |
| A15.3 | Tuberculosis of lung, confirmed by other and unspecified methods |
| A16.0 | Tuberculosis of lung, neither bacteriologically, molecular biologically nor histologically confirmed |
| A16.1 | Tuberculosis of lung, bacteriological, molecular biological and histological examination not performed |
| A16.2 | Tuberculosis of lung without information on bacteriological, molecular biological or histological confirmation |
| A20.2 | Pneumonic plague |
| A21.2 | Pulmonary tularemia |
| A22.1 | Pulmonary anthrax |
| A31.0 | Infection of the lung by other mycobacteria |
| A42.0 | Pulmonary actinomycosis |
| A43.0 | Pulmonary nocardiosis |
| A48.1 | Legionnaires' disease with pneumonia |
| B01.2 | Varicella pneumonia |
| B25.0 | Cytomegaloviral pneumonitis |
| B37.1 | Pulmonary candidiasis |
| B38.0 | Acute pulmonary coccidioidomycosis |
| B38.1 | Chronic pulmonary coccidioidomycosis |
| B38.2 | Pulmonary coccidioidomycosis, unspecified |
| B39.0 | Acute pulmonary histoplasmosis capsulati |
| B39.1 | Chronic pulmonary histoplasmosis capsulati |
| B39.2 | Pulmonary histoplasmosis capsulati, unspecified |
| B40.0 | Acute pulmonary blastomycosis |
| B40.1 | Chronic pulmonary blastomycosis |
| B40.2 | Pulmonary blastomycosis, unspecified |
| B41.0 | Pulmonary paracoccidioidomycosis |
| B42.0 | Pulmonary sporotrichosis |
| B44.0 | Invasive pulmonary aspergillosis |
| B44.1 | Other pulmonary aspergillosis |
| B45.0 | Pulmonary cryptococcosis |
| B46.0 | Pulmonary mucormycosis |
| B58.3 | Pulmonary toxoplasmosis |
| B67.1 | Echinococcus granulosus infection [cystic echinococcosis] of lung |
| J10.0 | Influenza due to identified seasonal influenza virus with pneumonia |
| J11.0 | Influenza with pneumonia, virus not detected |
| J12 | Viral pneumonia, not elsewhere classified |
| J12.9 | Viral pneumonia, unspecified |
| J13 | Pneumonia due to Streptococcus pneumoniae |
| J14 | Pneumonia due to Haemophilus influenzae |
| J15 | Bacterial pneumonia, not elsewhere classified |
| J16 | Pneumonia due to other infectious organisms, not elsewhere classified |
| J17 | Pneumonia in diseases classified elsewhere |
| J18.1 | Lobar pneumonia, unspecified organism |
| J18.2 | Hypostatic pneumonia, unspecified organism |
| J18.8 | Other pneumonia, unspecified organism |
| J18.9 | Pneumonia, unspecified organism |
| J69 | Pneumonitis due to solids and liquids |
| O29.0 | Pulmonary complications of anesthesia during pregnancy |
| O74.0 | Aspiration pneumonitis due to anesthesia during labor and delivery |
| U04 | Severe acute respiratory syndrome [SARS] |
| U69.0 | Hospital-acquired pneumonia classified elsewhere |

| **Focus of infection** |  |
| --- | --- |
| Respiratory tract, non-specific |  |
| ICD-10-GM codes |  |
| A15.5 | Tuberculosis of larynx, trachea and bronchus, confirmed by bacteriology, molecular biology or histology |
| A15.7 | Primary respiratory tuberculosis, confirmed by bacteriology, molecular biology or histology |
| A15.8 | Other respiratory tuberculosis, confirmed by bacteriology, molecular biology or histology |
| A15.9 | Respiratory tuberculosis unspecified, confirmed by bacteriology, molecular biology or histology |
| A16.4 | Tuberculosis of larynx, trachea and bronchus without information on bacteriological, molecular biological or histological confirmation |
| A16.7 | Primary respiratory tuberculosis without information on bacteriological, molecular biological or histological confirmation |
| A16.8 | Other respiratory tuberculosis without information on bacteriological, molecular biological or histological confirmation |
| A16.9 | Respiratory tuberculosis unspecified without information on bacteriological, molecular biological or histological confirmation |
| A37 | Whooping cough |
| J09 | Influenza due to identified zoonotic or pandemic influenza virus |
| J10.1 | Influenza due to identified seasonal influenza virus with other respiratory manifestations |
| J10.8 | Influenza due to identified seasonal influenza virus with other manifestations |
| J11.1 | Influenza due to unidentified influenza virus with other respiratory manifestations |
| J11.8 | Influenza due to unidentified influenza virus with other manifestations |
| J98.7 | Respiratory tract infection, not elsewhere classified |
| J99.8* | Diseases of the respiratory tract in diseases elsewhere classified |

| **Focus of infection** |  |
| --- | --- |
| Genitourinary system |  |
| ICD-10-GM codes |  |
| N29.1 | Other diseases of kidney and ureter in infectious and parasitic diseases classified elsewhere |
| T83.5 | Infection and inflammatory reaction due to prosthetic device, implant and graft in urinary system |
| N10 | Acute tubulo-interstitial nephritis |
| N11 | Chronic tubulo-interstitial nephritis |
| N12 | Tubulo-interstitial nephritis, not specified as acute or chronic |
| N13.6 | Pyonephrosis |
| N15.1 | Renal and perinephric abscess |
| N15.9 | Renal tubulo-interstitial disease, unspecified |
| N16.0 | Renal tubulo-interstitial disease in infectious and parasitic diseases classified elsewhere |
| N29.0 | Late syphilis of the kidney |
| N30.0 | Acute cystitis |
| N30.1 | Interstitial cystitis (chronic) |
| N30.2 | Other chronic cystitis |
| N30.8 | Other cystitis |
| N33* | Bladder disorders in diseases classified elsewhere |
| N34.0 | Urethral abscess |
| N34.1 | Nonspecific urethritis |
| N34.2 | Other urethritis |
| N37.0 | Urethral disorders in diseases classified elsewhere |
| N39.0 | Urinary tract infection, site not specified |

| **Focus of infection** |  |
| --- | --- |
| Wound and soft tissue infection |  |
| ICD-10-GM codes |  |
| A06.7 | Cutaneous amebiasis |
| A18.4 | Tuberculosis of skin and subcutaneous tissue |
| A18.5 | Tuberculosis of eye |
| A18.6 | Tuberculosis of (inner) (middle) ear |
| A20.1 | Cellulocutaneous plague |
| A22.0 | Cutaneous anthrax |
| A26.0 | Cutaneous erysipeloid |
| A31.1 | Cutaneous infection by other mykobacteria |
| A32.0 | Cutaneous listeriosis |
| A36.3 | Cutaneous diphtheria |
| A42.2 | Cervicofacial actinomycosis |
| A43.1 | Cutaneous nocardiosis |
| A44.1 | Cutaneous and mucocutaneous bartonellosis |
| A46 | Erysipelas |
| A48.0 | Gas gangrene |
| A67 | Pinta [carate] |
| A71 | Trachoma |
| B00.0 | Eczema herpeticum |
| B00.1 | Herpesviral vesicular dermatitis |
| B00.2 | Herpesviral gingivostomatitis and pharyngotonsillitis |
| B00.5 | Herpesviral ocular disease |
| B07 | Viral warts |
| B08 | Other viral infections characterized by skin and mucous membrane lesions, not elsewhere classified |
| B09 | Unspecified viral infection characterized by skin and mucous membrane lesions |
| B30 | Viral conjunctivitis |
| B35 | Dermatophytosis |
| B36 | Other superficial mycoses |
| B37.2 | Candidiasis of skin and nail |
| B38.3 | Cutaneous coccidioidomycosis |
| B40.3 | Cutaneous blastomycosis |
| B42.1 | Lymphocutaneous sporotrichosis |
| B43.0 | Cutaneous chromomycosis |
| B43.2 | Subcutaneous chromomycotic abscess and cyst |
| B44.2 | Tonsillar aspergillosis |
| B45.2 | Cutaneous cryptococcosis |
| B46.3 | Cutaneous mucormycosis |
| B55.1 | Cutaneous leishmaniasis |
| B58.0 | Toxoplasma oculopathy |
| B85 | Pediculosis and phthiriasis |
| B86 | Scabies |
| B87.0 | Cutaneous myiasis |
| B87.1 | Wound myiasis |
| B87.2 | Ocular myiasis |
| B87.4 | Aural myiasis |
| B88 | Other parasitic infestation of the skin |
| E06.0 | Acute thyroiditis |
| E32.1 | Abscess of thymus |
| H00.0 | Hordeolum and other deep eyelid inflammation |
| H03.0 | Parasitic infestation of the eyelid in diseases classified elsewhere |
| H03.1 | Involvement of the eyelid in other infectious diseases classified elsewhere |
| H04.3 | Acute and unspecified inflammation of lacrimal passages |
| H10.0 | Mucopurulent conjunctivitis |
| H13.0 | Filarial infestation of the conjunctiva |
| H13.1 | Conjunctivitis in infectious and parasitic diseases classified elsewhere |
| H15 | Scleritis |
| H16 | Keratitis |
| H19.0 | Scleritis and episcleritis in diseases classified elsewhere |
| H19.1 | Keratitis and keratoconjunctivitis caused by herpes viruses |
| H19.2 | Keratitis and keratoconjunctivitis in other infectious and parasitic diseases classified elsewhere |
| H20.0 | Acute and subacute iridocyclitis |
| H22.0 | Iridozyklitis in other infectious and parasitic diseaeses classified elsewhere |
| H30 | Chorioretinitis |
| H32.0 | Chorioretinitis in other infectious and parasitic diseases classified elsewhere |
| H44.0 | Purulent endophthalmitis |
| H44.1 | Other endophthalmitis |
| H45.1 | Endophthalmitis in diseases classified elsewhere |
| H60.0 | Abscess of external ear |
| H60.1 | Phlegmon of external ear |
| H60.2 | Malignant otitis externa |
| H60.3 | Other infective otitis externa |
| H60.8 | Other otitis externa |
| H60.9 | Unspecified otitis externa |
| H62.0 | Otitis externa in bacterial diseases classified elsewhere |
| H62.1 | Otitis externa in viral diseases classified elsewhere |
| H62.2 | Otitis externa in mycoses classified elsewhere |
| H62.3 | Otitis externa in other infectious and parasitic diseases classified elsewhere |
| H65 | Nonsuppurative otitis media |
| H66 | Suppurative and unspecified otitis media |
| H67 | Otitis media in diseases classified elsewhere |
| H74.1 | Otitis media adhaesiva |
| J03 | Acute tonsillitis |
| J34.0 | Abscess, furuncle and carbuncle of nose |
| J35.0 | Chronic tonsillitis |
| J36 | Peritonsillar abscess |
| J39.0 | Retropharyngeal and parapharyngeal abscess |
| J39.1 | Other abscess of pharynx |
| K04.0 | Pulpitis |
| K04.6 | Periapical abscess with fistula |
| K04.7 | Periapical abscess without fistula |
| K05.0 | Acute gingivitis |
| K05.1 | Chronic gingivitis |
| K11.3 | Abscess of salivary gland |
| K12.2 | Phlegmon and abscess of mouth |
| K12.3 | Oral mucositis (ulcerative) |
| K14.0 | Glossitis |
| K61 | Abscess of anal and rectal regions |
| L01 | Impetigo |
| L02 | Cutaneous abscess, furuncle and carbuncle |
| L03 | Phlegmon |
| L04 | Acute lymphadenitis |
| L05 | Pilonidal cyst |
| L08 | Other local infections of skin and subcutaneous tissue |
| L30.3 | Eczematoid dermatitis |
| M60.0 | Infective myositis |
| M63.0 | Myositis in bacterial diseases classified elsewhere |
| M63.1 | Myositis in protozoal and parasitic infections classified elsewhere |
| M63.2 | Myositis in other infectious diseases classified elsewhere |
| M72.6 | Necrotizing fasciitis |
| M72.8 | Other fibromatoses |
| N61 | Inflammatory disorders of breast [breast gland] |
| O91 | Infections of breast [breast gland] associated with gestation |
| T79.3 | Posttraumatic wound infection, not elsewhere classified |
| T87.4 | Infection of amputation stump |

| **Focus of infection** |  |
| --- | --- |
| Intraabdominal and retroperitoneal |  |
| ICD-10-GM codes |  |
| A42.1 | Abdominal actinomycosis |
| K65 | Peritonitis |
| K67 | Disorders of peritoneum in infectious diseases classified elsewhere |
| K80.0 | Calculus of gallbladder with acute cholecystitis |
| K80.1 | Calculus of gallbladder with other cholecystitis |
| K80.3 | Calculus of bile duct with cholangitis |
| K80.4 | Calculus of bile duct with cholecystitis |
| K81.0 | Acute cholecystitis |
| K83.08 | Other cholangitis |
| A06.4 | Amebic liver abscess |
| A18.7 | Tuberculosis of adrenal glands |
| B15 | Acute hepatitis A |
| B16 | Acute hepatitis B |
| B17 | Other acute viral hepatitis |
| B18 | Chronic viral hepatitis |
| B19 | Unspecified viral hepatitis |
| B25.1 | Cytomegaloviral hepatitis |
| B25.2 | Cytomegaloviral pancreatitis |
| B26.3 | Mumps pancreatitis |
| B58.1 | Toxoplasma hepatitis |
| B67.0 | Echinococcus granulosus infection [cystic echinococcosis] of liver |
| B67.5 | Echinococcus multilocularis infection [alveolar echinococcosis] of liver |
| B67.8 | Echinococcosis, unspecified, of liver |
| D73.3 | Abscess of spleen |
| K75.0 | Abscess of liver |
| K77.0 | Liver disorders in infectious and parasitic diseases classified elsewhere |
| K85.01 | Idiopathic acute pancreatitis with organ complication |
| K85.11 | Biliary acute pancreatitis with organ complication |
| K85.21 | Alcohol induced acute pancreatitis with organ complication |
| K85.31 | Drug induced acute pancreatitis with organ complication |
| K85.81 | Other acute pancreatitis with organ complication |
| K85.91 | Acute pancreatitis, unspecified, with organ complication |
| K86.1 | Other chronic pancreatitis |
| A06.3 | Ameboma of intestine |
| A18.3 | Tuberculosis of intestines, peritoneum and mesenteric glands |
| A22.2 | Gastrointestinal anthrax |
| B25.80 | Infection of the digestive tract by cytomegaloviruses |
| B46.2 | Gastrointestinal mucormycosis |
| K35 | Acute appendicitis |
| K36 | Other appendicitis |
| K37 | Unspecified appendicitis |
| K57.0 | Diverticulitis of small intestine with perforation and abscess |
| K57.12 | Diverticulitis of small intestine without perforation or abscess without bleeding |
| K57.13 | Diverticulitis of small intestine without perforation or abscess with bleeding |
| K57.2 | Diverticulitis of large intestine with perforation and abscess |
| K57.32 | Diverticulitis of large intestine without perforation or abscess without bleeding |
| K57.33 | Diverticulitis of large intestine without perforation or abscess with bleeding |
| K57.4 | Diverticulitis of both small and large intestine with perforation and abscess |
| K57.52 | Diverticulitis of both small and large intestine without perforation or abscess without bleeding |
| K57.53 | Diverticulitis of both small and large intestine without perforation or abscess with bleeding |
| K57.8 | Diverticulitis of intestine, part unspecified, with perforation and abscess |
| K57.92 | Diverticulitis of intestine, part unspecified, without perforation or abscess without bleeding |
| K57.93 | Diverticulitis of intestine, part unspecified, without perforation or abscess with bleeding |
| K63.0 | Abscess of intestine |
| K93.0 | Tuberculosis of intestines, peritoneum and mesenteric glands |

| **Focus of infection** |  |
| --- | --- |
| Intrathoracic |  |
| ICD-10-GM codes |  |
| A15.4 | Tuberculosis of intrathoracic lymph nodes, confirmed by bacteriology, molecular biology or histology |
| A16.3 | Tuberculosis of intrathoracic lymph nodes without information on bacteriological, molecular biological or histological confirmation |
| K20 | Esophagitis |
| K23.0* | Tuberculosis of esophagus |
| A15.6 | Tuberculous pleurisy, confirmed by bacteriology, molecular biology or histology |
| A16.5 | Tuberculous pleurisy without information on bacteriological, molecular biological or histological confirmation |
| J86 | Pyothorax |
| A39.5 | Meningococcal heart disease |
| I30.1 | Infective pericarditis |
| I32.0 | Pericarditis in bacterial diseases classified elsewhere |
| I32.1* | Pericarditis in other infectious and parasitic diseases classified elsewhere |
| I40 |  |
| I41.0* | Myocarditis in bacterial diseases classified elsewhere |
| I41.1* | Myocarditis in viral diseases classified elsewhere |
| I41.2* | Myocarditis in other infectious and parasitic diseases classified elsewhere |
| I51.4 | Myocarditis, unspecified |
| J85 | Abscess of lung and mediastinum |
| J98.50 | Mediastinitis |

| **Focus of infection** |  |
| --- | --- |
| Central nervous system |  |
| ICD-10-GM codes |  |
| A06.6 | Amebic brain abscess |
| A17 | Tuberculosis of nervous system |
| A20.3 | Plague meningitis |
| A32.1 | Listerial meningitis and meningoencephalitis |
| A39.0 | Meningococcal meningitis |
| A52.1 | Symptomatic neurosyphilis |
| A52.2 | Asymptomatic neurosyphilis |
| A52.3 | Neurosyphilis, unspecified |
| A80 | Acute poliomyelitis [spinal polio] |
| A81 | Atypical virus infections of central nervous system |
| A82 | Rabies |
| A83 | Mosquito-borne viral encephalitis |
| A84 | Tick-borne viral encephalitis |
| A85 | Other viral encephalitis, not elsewhere classified |
| A86 | Unspecified viral encephalitis |
| A87 | Viral meningitis |
| A88 | Other viral infections of central nervous system, not elsewhere classified |
| A89 | Unspecified viral infection of central nervous system |
| B00.3 | Herpesviral meningitis |
| B00.4 | Herpesviral encephalitis |
| B01.0 | Varicella meningitis |
| B01.1 | Varicella encephalitis |
| B02.0 | Zoster encephalitis |
| B02.1 | Zoster meningitis |
| B02.2 | Zoster with other nervous system involvement |
| B02.3 | Zoster ocular disease |
| B26.1 | Mumps meningitis |
| B26.2 | Mumps encephalitis |
| B37.5 | Candidal meningitis |
| B38.4 | Coccidioidomycosis meningitis |
| B43.1 | Chromomycotic brain abscess |
| B45.1 | Cerebral cryptococcosis |
| B46.1 | Rhinocerebral mucormycosis |
| B58.2 | Toxoplasma meningoencephalitis |
| G00 | Bacterial meningitis, not elsewhere classified |
| G01 | Meningitis in bacterial diseases classified elsewhere |
| G02* | Meningitis in other infectious and parasitic diseases classified elsewhere |
| G03 | Meningitis due to other and unspecified causes |
| G04 | Encephalitis, myelitis and encephalomyelitis |
| G05** | Encephalitis, myelitis and encephalomyelitis in diseases classified elsewhere |
| G06 | Intracranial and intraspinal abscess and granuloma |
| G07 | Intracranial and intraspinal abscess and granuloma in diseases classified elsewhere |
| G94.31 | Encephalopathy in infectious and parasitic diseases classified elsewhere |
| G94.32* | septic encephalopathy |
| H73.0 | Acute myringitis |
| H73.1 | Chronic myringitis |
| H83.0 | Labyrinthitis |
| H94.0 | Inflammation of the vestibulocochlear nerve [VIII cranial nerve] in infectious and parasitic diseases classified elsewhere |

| **Focus of infection** |  |
| --- | --- |
| Bloodstream and vascular infection |  |
| ICD-10-GM codes |  |
| A52.0 | Cardiovascular syphilis |
| I68.1* | Cerebral arteritis in infectious and parasitic diseases classified elsewhere |
| I98.0* | Cardiovascular syphilis |
| O88.3 | Obstetric pyemic and septic embolism |
| T82.7 | Infection and inflammatory reaction due to other cardiac and vascular devices, implants and grafts |
| I33 | Acute and subacute endocarditis |
| I38 | Endocarditis, valve unspecified |
| I39 | Endocarditis and heart valve disorders in diseases classified elsewhere |
| T82.6 | Infection and inflammatory reaction due to cardiac valve prosthesis |
| G08 | Intracranial and intraspinal phlebitis and thrombophlebitis |
| I80 | Thombosis, phlebitis and thrombophlebitis |
| K75.1 | Phlebitis of portal vein |
| O22.2 | Superficial thrombophlebitis in pregnancy |
| O87.0 | Superficial thrombophlebitis in the puerperium |
| O87.1 | Deep phlebothrombosis in the puerperium |
| O87.9 | Venous complication in the puerperium, unspecified |

| **Focus of infection** |  |
| --- | --- |
| Bones, cartilage, joints |  |
| ICD-10-GM codes |  |
| A18.0 | Tuberculosis of bones and joints |
| B45.3 | Osseous cryptococcosis |
| B67.2 | Echinococcus granulosus infection [cystic echinococcosis] of bone |
| H05.0 | Acute inflammation of orbit |
| H06.1* | Parasitic infestation of the orbit in diseases classified elsewhere |
| H61.0 | Perichondritis of external ear |
| H70 | Mastoiditis and related conditions |
| H75.0 | Mastoiditis in infectious and parasitic diseases classified elsewhere |
| H95.0 | Recurrent cholesteatoma of postmastoidectomy cavity |
| K04.4 | Acute apical periodontitis of pulpal origin |
| K04.5 | Chronic apical periodontitis |
| K05.2 | Aggressive periodontitis |
| K05.3 | Chronic periodontitis |
| K10.2- | Inflammatory conditions of the jaw |
| M00 | Pyogenic arthritis |
| M01 | Direct infections of joint in infectious and parasitic diseases classified elsewhere |
| M65.0 | Abscess of tendon sheath |
| M65.1 | Other infective (teno)synovitis |
| M68.0 | Synovitis and tenosynovitis in bacterial diseases classified elsewhere |
| M71.0 | Abscess of bursa |
| M71.1 | Other infective bursitis |
| M86 | Osteomyelitis |
| M90.0 | Tuberculosis of bone |
| M90.1 | Periostitis in other infectious diseases classified elsewhere |
| T84.5 | Infection and inflammatory reaction due to internal joint prosthesis |
| T84.6 | Infection and inflammatory reaction due to internal fixation device [any site] |
| T84.7 | Infection and inflammatory reaction due to other internal orthopedic prosthetic devices, implants and grafts |
| M46.2 | Osteomyelitis of vertebra |
| M46.3 | Infection of intervertebral disc (pyogenic) |
| M46.4 | Discitis, unspecified |
| M46.5 | Other infective spondylopathies |
| M49.0 | Tuberculosis of spine |
| M49.1 | Spondylitis brucellosa |
| M49.2 | Spondylitis due to bacterial intestinal infection |
| M49.3 | Spondylopathy in other infectious and parasitic diseases classified elsewhere |

| **Focus of infection** |  |
| --- | --- |
| Gastrointestinal tract and diarrhea |  |
| ICD-10-GM codes |  |
| A00 | Cholera |
| A02.0 | Salmonella enteritis |
| A03 | Shigellosis |
| A04 | Other bacterial intestinal infections |
| A05 | Other bacterial foodborne intoxications, not elsewhere classified |
| A06.0 | Acute amebic dysentery |
| A06.1 | Chronic intestinal amebiasis |
| A06.2 | Amebic nondysenteric colitis |
| A07 | Other protozoal intestinal diseases |
| A08 | Viral and other specified intestinal infections |
| A09 | Other gastroenteritis and colitis of infectious and unspecified origin |
| A21.3 | Gastrointestinal tularemia |
| B37.0 | Candidal stomatitis |
| B37.81 | Candidal esophagitis |
| B79 | Trichuriasis |
| B80 | Enterobiasis |
| B81 | Other intestinal helminthiases, not elsewhere classified |
| B82 | Unspecified intestinal parasitism |
| U69.40! | Recurrent infection with Clostridium difficile |

| **Focus of infection** |  |
| --- | --- |
| Systemic viral infection |  |
| ICD-10-GM codes |  |
| A92 | Other mosquito-borne viral fevers |
| A93 | Other arthropod-borne viral fevers, not elsewhere classified |
| A94 | Unspecified arthropod-borne viral fever |
| A95 | Yellow fever |
| A96 | Arenaviral hemorrhagic fever |
| A97 | Dengue |
| A98 | Other viral hemorrhagic fevers, not elsewhere classified |
| A99 | Unspecified viral hemorrhagic fever |
| B00 | Herpesviral [herpes simplex] infections |
| B00.8 | Other forms of herpesviral infections |
| B00.9 | Herpesviral infection, unspecified |
| B01.8 | Varicella with other complications |
| B01.9 | Varicella without complication |
| B02.7 | Disseminated zoster |
| B02.8 | Zoster with other complications |
| B02.9 | Zoster without complications |
| B03 | Smallpox |
| B04 | Monkeypox |
| B05 | Measles |
| B06 | Rubella [German measles] |
| B25.8 | Other cytomegaloviral diseases |
| B25.88 | Other cytomegaloviral diseases |
| B25.9 | Cytomegaloviral disease, unspecified |
| B26.8 | Mumps with other complications |
| B26.9 | Mumps without complication |
| B27 | Infectious mononucleosis |
| B33 | Other viral diseases, not elsewhere classified |
| B34 | Viral infection of unspecified site |
| U07.1! | Laboratory-confirmed SARS-CoV-2 infection |
| U10.9 | Multisystemic inflammatory syndrome associated with COVID-19, unspecified |

| **Focus of infection** |  |
| --- | --- |
| Infections of the genital tract and sexually transmitted diseases |  |
| ICD-10-GM codes |  |
| A50 | Congenital syphilis |
| A51 | Early syphilis |
| A52.7 | Other symptomatic late syphilis |
| A52.8 | Late syphilis, latent |
| A52.9 | Late syphilis, unspecified |
| A53 | Other and unspecified syphilis |
| A54.0 | Gonococcal infection of lower genitourinary tract without periurethral or accessory gland abscess |
| A54.1 | Gonococcal infection of lower genitourinary tract with periurethral and accessory gland abscess |
| A54.2 | Gonococcal pelviperitonitis and other gonococcal genitourinary infection |
| A54.3 | Gonococcal infection of eye |
| A54.4 | Gonococcal infection of musculoskeletal system |
| A54.5 | Gonococcal pharyngitis |
| A54.6 | Gonococcal infection of anus and rectum |
| A54.9 | Gonococcal infection, unspecified |
| A55 | Chlamydial lymphogranuloma (venereum) |
| A56 | Other sexually transmitted chlamydial diseases |
| A57 | Chancroid |
| A58 | Granuloma inguinale |
| A59 | Trichomoniasis |
| A60 | Anogenital herpesviral [herpes simplex] infections |
| A63 | Other predominantly sexually transmitted diseases, not elsewhere classified |
| A64 | Unspecified sexually transmitted disease |
| A66 | Yaws |
| B20 | Infectious and parasitic diseases due to HIV disease [human immunodeficiency virus disease] |
| B21 | Malignant neoplasms due to HIV disease [human immunodeficiency virus disease] |
| B22 | Other specified diseases due to HIV disease [human immunodeficiency virus disease] |
| B23 | Other medical conditions due to HIV disease [human immunodeficiency virus disease] |
| B24 | Unspecified HIV disease [human immunodeficiency virus disease] |
| B26.0 | Mumps orchitis |
| B37.3 | Candidiasis of vulva and vagina |
| N41 | Inflammatory diseases of prostate |
| N43.1 | Infected hydrocele |
| N45 | Orchitis and epididymitis |
| N48.1 | Balanitis |
| N48.2 | Other inflammatory disorders of penis |
| N49 | Inflammatory disorders of male genital organs, not elsewhere classified |
| N51* | Disorders of male genital organs in diseases classified elsewhere |
| N70 | Salpingitis and oophoritis |
| N71 | Inflammatory disease of uterus, except cervix |
| N72 | Inflammatory disease of cervix uteri |
| N73 | Other female pelvic inflammatory diseases |
| N74* | Female pelvic inflammatory disorders in diseases classified elsewhere |
| N75.1 | Abscess of Bartholin's gland |
| N75.8 | Other diseases of Bartholin's gland |
| N76 | Other inflammation of vagina and vulva |
| N77.0* | Ulceration of vulva in infectious and parasitic diseases classified elsewhere |
| N77.1* | Vaginitis, vulvitis or vulvovaginitis in infectious and parasitic diseases classified elsewhere |
| N98.0 | Infection associated with artificial insemination |
| O03.0 | Spontaneous abortion, complicated by genital tract and pelvic infection, incomplete |
| O04.0 | Medical abortion, complicated by genital tract and pelvic infection, incomplete |
| O05.0 | Other abortion, complicated by genital tract and pelvic infectio, incomplete |
| O06.0 | Unspecified abortion, complicated by genital tract and pelvic infection, incomplete |
| O03.5 | Spontaneous abortion, complicated by genital tract and pelvic infection, complete or unspecified |
| O04.5 | Medical abortion, complicated by genital tract and pelvic infection, complete or unspecified |
| O05.5 | Other abortion, complicated by genital tract and pelvic infection, complete or unspecified |
| O06.5 | Unspecified abortion, complicated by genital tract and pelvic infection, complete or unspecified |
| O07.0 | Failed medical abortion, complicated by genital tract and pelvic infection |
| O07.5 | Other and unspecified failed attempted abortion, complicated by genital tract and pelvic infection |
| O08.0 | Genital tract and pelvic infection following abortion and ectopic and molar pregnancy |
| T83.6 | Infection and inflammatory reaction due to prosthetic device, implant and graft in genital tract |
| U60! | Clinical categories of HIV disease [human immunodeficiency virus disease] |
| U61! | Number of T helper cells in HIV disease [human immunodeficiency virus disease] |
| U85! | Human immunodeficiency virus with resistance to virustatics or proteinase inhibitors |
| Z21 | Asymptomatic HIV infection [human immunodeficiency virus infection] |

| **Focus of infection** |  |
| --- | --- |
| Non-specific |  |
| ICD-10-GM codes |  |
| A01 | Typhoid and paratyphoid fevers |
| A02.2 | Localized salmonella infections |
| A02.8 | Other specified salmonella infections |
| A02.9 | Salmonella infection, unspecified |
| A06.8 | Amebic infection of other sites |
| A06.9 | Amebiasis, unspecified |
| A18.1 | Tuberculosis of genitourinary system |
| A18.2 | Tuberculous peripheral lymphadenopathy |
| A18.8 | Tuberculosis of other specified organs |
| A19 | Miliary tuberculosis |
| A20.8 | Other forms of plague |
| A20.9 | Plague, unspecified |
| A21.0 | Ulceroglandular tularemia |
| A21.1 | Oculoglandular tularemia |
| A21.8 | Other forms of tularemia |
| A21.9 | Tularemia, unspecified |
| A22.8 | Other forms of anthrax |
| A22.9 | Anthrax, unspecified |
| A23 | Brucellosis |
| A24.0 | Glanders |
| A24.2 | Subacute and chronic melioidosis |
| A24.3 | Other melioidosis |
| A24.4 | Melioidosis, unspecified |
| A25 | Rat-bite fevers |
| A26.8 | Other forms of erysipeloid |
| A26.9 | Erysipeloid, unspecified |
| A27 | Leptospirosis |
| A28.0 | Pasteurellosis |
| A28.1 | Cat-scratch disease |
| A28.8 | Other specified zoonotic bacterial diseases, not elsewhere classified |
| A28.9 | Zoonotic bacterial disease, unspecified |
| A30 | Leprosy [Hansen's disease] |
| A31.8 | Other mycobacterial infections |
| A31.80 | Disseminated atypical mycobacteriosis |
| A31.88 | Other infections caused by mycobacteria |
| A31.9 | Infection caused by mycobacteria, unspecified |
| A32.8 | Other forms of listeriosis |
| A32.9 | Listeriosis, unspecified |
| A34 | Obstetrical tetanus |
| A35 | Other tetanus |
| A36.8 | Other diphtheria |
| A36.9 | Diphtheria, unspecified |
| A38 | Scarlet fever |
| A39.8 | Other meningococcal infections |
| A39.9 | Meningococcal infection, unspecified |
| A42.8 | Other forms of actinomycosis |
| A42.9 | Actinomycosis, unspecified |
| A43.8 | Other forms of nocardiosis |
| A43.9 | Nocardiosis, unspecified |
| A44.0 | Systemic bartonellosis |
| A44.8 | Other forms of bartonellosis |
| A44.9 | Bartonellosis, unspecified |
| A48.2 | Nonpneumonic Legionnaires' disease [Pontiac fever] |
| A48.4 | Brazilian purpuric fever |
| A48.8 | Other specified bacterial diseases |
| A49.0 | Staphylococcal infection, unspecified site |
| A49.1 | Streptococcal and enterococcal infection, unspecified site |
| A49.2 | Hemophilus influenzae infection, unspecified site |
| A49.3 | Mycoplasma infection, unspecified site |
| A49.8 | Other bacterial infections of unspecified site |
| A65 | Nonvenereal syphilis |
| A68 | Relapsing fevers |
| A69 | Other spirochetal infections |
| A70 | Chlamydia psittaci infections |
| A74 | Other diseases caused by chlamydiae |
| A75 | Typhus fever |
| A77 | Spotted fever [tick-borne rickettsioses] |
| A78 | Q fever |
| A79 | Other rickettsioses |
| B37.4 | Candidiasis of other urogenital sites |
| B37.8 | Candidiasis of other sites |
| B37.88 | Candidiasis of other sites |
| B37.9 | Candidiasis, unspecified |
| B38.7 | Disseminated coccidioidomycosis |
| B38.8 | Other forms of coccidioidomycosis |
| B38.9 | Coccidioidomycosis, unspecified |
| B39.3 | Disseminated histoplasmosis capsulati |
| B39.4 | Histoplasmosis capsulati, unspecified |
| B39.5 | Histoplasmosis duboisii |
| B39.9 | Histoplasmosis, unspecified |
| B40.7 | Disseminated blastomycosis |
| B40.8 | Other forms of blastomycosis |
| B40.9 | Blastomycosis, unspecified |
| B41.7 | Disseminated paracoccidioidomycosis |
| B41.8 | Other forms of paracoccidioidomycosis |
| B41.9 | Paracoccidioidomycosis, unspecified |
| B42.7 | Disseminated sporotrichosis |
| B42.8 | Other forms of sporotrichosis |
| B42.9 | Sporotrichosis, unspecified |
| B43.8 | Other forms of chromomycosis |
| B43.9 | Chromomycosis, unspecified |
| B44.7 | Disseminated aspergillosis |
| B44.8 | Other forms of aspergillosis |
| B44.9 | Aspergillosis, unspecified |
| B45.7 | Disseminated cryptococcosis |
| B45.8 | Other forms of cryptococcosis |
| B45.9 | Cryptococcosis, unspecified |
| B46.4 | Disseminated mucormycosis |
| B46.5 | Mucormycosis, unspecified |
| B46.8 | Other zygomycoses |
| B46.9 | Zygomycosis, unspecified |
| B47 | Mycetoma |
| B48 | Other mycoses, not elsewhere classified |
| B50 | Plasmodium falciparum malaria |
| B51 | Plasmodium vivax malaria |
| B52 | Plasmodium malariae malaria |
| B53 | Other specified malaria |
| B54 | Unspecified malaria |
| B55.0 | Visceral leishmaniasis |
| B55.2 | Mucocutaneous leishmaniasis |
| B55.9 | Leishmaniasis, unspecified |
| B56 | African trypanosomiasis |
| B57 | Chagas' disease |
| B58.8 | Toxoplasmosis with other organ involvement |
| B58.9 | Toxoplasmosis, unspecified |
| B60 | Other protozoal diseases, not elsewhere classified |
| B64 | Unspecified protozoal disease |
| B65 | Schistosomiasis [bilharziasis] |
| B66 | Other fluke infections |
| B67.3 | Echinococcus granulosus infection [cystic echinococcosis], other and multiple sites |
| B67.4 | Echinococcus granulosus infection [cystic echinococcosis], unspecified |
| B67.6 | Echinococcus multilocularis infection [alveolar echinococcosis], other and multiple sites |
| B67.7 | Echinococcus multilocularis infection [alveolar echinococcosis], unspecified |
| B67.9 | Echinococcosis, other and unspecified |
| B68 | Taeniasis |
| B69 | Cysticercosis |
| B70 | Diphyllobothriasis and sparganosis |
| B72 | Dracunculiasis |
| B73 | Onchocerciasis |
| B74 | Filariasis |
| B75 | Trichinellosis |
| B76 | Hookworm diseases |
| B77 | Ascariasis |
| B78 | Strongyloidiasis |
| B83 | Other helminthiases |
| B87.8 | Myiasis of other sites |
| B87.9 | Myiasis, unspecified |
| B89 | Unspecified parasitic disease |
| B95 | Streptococcus and Staphylococcus as the cause of diseases classified elsewhere |
| B96 | Other bacterial agents as the cause of diseases classified elsewhere |
| B97 | Viral agents as the cause of diseases classified elsewhere |
| B98 | Other infectious agents as the cause of diseases classified elsewhere |
| B99 | Other and unspecified infectious diseases |
| L00 | Staphylococcal scalded skin syndrome |
| O08.2 | Embolism following abortion and ectopic and molar pregnancy |
| O23 | Infections of genitourinary tract in pregnancy |
| O41.1 | Infection of amniotic sac and membranes |
| O75.2 | Pyrexia during labor, not elsewhere classified |
| O86 | Other puerperal infections |
| O89.0 | Pulmonary complications of anesthesia during the puerperium |
| O98 | Maternal infectious and parasitic diseases classifiable elsewhere but complicating pregnancy, childbirth and the puerperium |
| T85.7 | Infection and inflammatory reaction due to other internal prosthetic devices, implants and grafts |
| T89.01 | Other specified complications of trauma, foreign body (with or without infection) |
| T89.02 | Other specified complications of trauma, infection |
| U80 | Gram-positive pathogens with certain antibiotic resistances that require special therapeutic or hygienic measures |
| U81 | Gram-negative pathogens with certain antibiotic resistances that require special therapeutic or hygienic measures |
| U82! | Mycobacteria with resistance to antituberculotics (first-line drugs) |
| U83! | Human pathogenic fungi with resistance to antimycotics |
| U84! | Herpes viruses with resistance to virustatics |

| **Focus of infection** |  |
| --- | --- |
| Device-related infection |  |
| ICD-10-GM codes |  |
| T82.6 | Infection and inflammatory reaction due to cardiac valve prosthesis |
| T82.7 | Infection and inflammatory reaction due to other cardiac and vascular devices, implants and grafts |
| T83.5 | Infection and inflammatory reaction due to prosthetic device, implant and graft in urinary system |
| T83.6 | Infection and inflammatory reaction due to prosthetic device, implant and graft in genital tract |
| T84.5 | Infection and inflammatory reaction due to internal joint prosthesis |
| T84.6 | Infection and inflammatory reaction due to internal fixation device [any site] |
| T84.7 | Infection and inflammatory reaction due to other internal orthopedic prosthetic devices, implants and grafts |
| T85.7 | Infection and inflammatory reaction due to other internal prosthetic devices, implants and grafts |
| T89.01 | Other specified complications of trauma, foreign body (with or without infection) |

| **Focus of infection** |  |
| --- | --- |
| Nosocomial infection, pneumonia |  |
| ICD-10-GM code |  |
| U69.0 | Hospital-acquired pneumonia in patients aged 18 years or older |

| **Therapies received during index hospitalization** |  |
| --- | --- |
| Dialysis |  |
| OPS codes |  |
| 8-821.2 | Plasmapheresis, immunoadsorption and related procedures: Immunoadsorption and related procedures: Adsorption to remove hydrophobic substances (low and/or moderate molecular weight) |
| 8-853.1 | Extracorporeal circulation and treatment of blood: Haemofiltration: Continuous arteriovenous haemofiltration (CAVH) |
| 8-853.3 | Extracorporeal circulation and treatment of blood: Haemofiltration: Intermittent haemofiltration, anticoagulation with heparin or without anticoagulation |
| 8-853.4 | Extracorporeal circulation and treatment of blood: Haemofiltration: Intermittent haemofiltration, anticoagulation with other substances |
| 8-853.5 | Extracorporeal circulation and treatment of blood: Haemofiltration: Prolonged intermittent haemofiltration, anticoagulation with heparin or without anticoagulation |
| 8-853.6 | Extracorporeal circulation and treatment of blood: Haemofiltration: Prolonged intermittent haemofiltration, anticoagulation with other substances |
| 8-853.7 | Extracorporeal circulation and treatment of blood: Haemofiltration: Pump-assisted continuous venovenous haemofiltration (CVVH), anticoagulation with heparin or without anticoagulation |
| 8-853.8 | Extracorporeal circulation and treatment of blood: Haemofiltration: Pump-assisted continuous venovenous haemofiltration (CVVH), anticoagulation with other substances |
| 8-853.x | Extracorporeal circulation and treatment of blood: Haemofiltration: Other |
| 8-853.y | Extracorporeal circulation and treatment of blood: Haemofiltration: Unspecified |
| 8-854.2 | Extracorporeal circulation and treatment of blood: Haemodialysis: Intermittent haemofiltration, anticoagulation with heparin or without anticoagulation |
| 8-854.3 | Extracorporeal circulation and treatment of blood: Haemodialysis: Intermittent haemofiltration, anticoagulation with other substances |
| 8-854.4 | Extracorporeal circulation and treatment of blood: Haemodialysis: Prolonged intermittent haemofiltration, anticoagulation with heparin or without anticoagulation |
| 8-854.5 | Extracorporeal circulation and treatment of blood: Haemodialysis: Prolonged intermittent haemofiltration, anticoagulation with other substances |
| 8-854.6 | Extracorporeal circulation and treatment of blood: Haemodialysis: Pump-assisted continuous venovenous haemofiltration (CVVHD), anticoagulation with heparin or without anticoagulation |
| 8-854.7 | Extracorporeal circulation and treatment of blood: Haemodialysis: Pump-assisted continuous venovenous haemofiltration (CVVHD), anticoagulation with other substances |
| 8-854.8 | Extracorporeal circulation and treatment of blood: Haemodialysis: Prolonged intermittent haemodialysis to clear protein molecules up to 60,000 |
| 8-854.x | Extracorporeal circulation and treatment of blood: Haemodialysis: Other |
| 8-854.y | Extracorporeal circulation and treatment of blood: Haemodialysis: Unspecified |
| 8-855.1 | Extracorporeal circulation and treatment of blood: Haemodiafiltration: Continuous arteriovenous haemofiltration (CAVHDF) |
| 8-855.3 | Extracorporeal circulation and treatment of blood: Haemodiafiltration: Intermittent haemofiltration, anticoagulation with heparin or without anticoagulation |
| 8-855.4 | Extracorporeal circulation and treatment of blood: Haemodiafiltration: Intermittent haemofiltration, anticoagulation with other substances |
| 8-855.5 | Extracorporeal circulation and treatment of blood: Haemodiafiltration: Prolonged intermittent haemofiltration, anticoagulation with heparin or without anticoagulation |
| 8-855.6 | Extracorporeal circulation and treatment of blood: Haemodiafiltration: Prolonged intermittent haemofiltration, anticoagulation with other substances |
| 8-855.7 | Extracorporeal circulation and treatment of blood: Haemodiafiltration: Pump-assisted continuous venovenous haemofiltration (CVVHDF), anticoagulation with heparin or without anticoagulation |
| 8-855.8 | Extracorporeal circulation and treatment of blood: Haemodiafiltration: Pump-assisted continuous venovenous haemofiltration (CVVHDF), anticoagulation with other substances |
| 8-855.x | Extracorporeal circulation and treatment of blood: Haemodiafiltration: Other |
| 8-855.y | Extracorporeal circulation and treatment of blood: Haemodiafiltration: Unspecified |
| 8-856 | Extracorporeal circulation and treatment of blood: Haemoperfusion |
| 8-85a.0 | Extracorporeal circulation and treatment of blood: Dialysis following no graft function and failure of kidney transplant: Intermittent |
| 8-85a.1 | Extracorporeal circulation and treatment of blood: Dialysis following no graft function and failure of kidney transplant: Continuous |

| Extracorporeal membrane oxygenation (ECMO) |  |
| --- | --- |
| OPS code |  |
| 8-852 | Extracorporeal circulation and treatment of blood: Extracorporeal gas exchange without and with cardiac assist and pre-ECMO therapy |

| Mechanical ventilation |  |
| --- | --- |
| OPS codes |  |
| 8-701 | Access during mechanical ventilation and using airway clearance techniques: Simple endotracheal intubation |
| 8-704 | Access during mechanical ventilation and using airway clearance techniques: Intubation with double-lumen tube |
| 8-706 | Access during mechanical ventilation and using airway clearance techniques: Mechanical ventilation via face mask |
| 5-311 | Other larynx surgery and surgery on the trachea: Temporary tracheostomy |
| 5-312 | Other larynx surgery and surgery on the trachea: Permanent tracheostomy |

| Extracorporeal liver replacement therapy |  |
| --- | --- |
| OPS code |  |
| 8-858 | Extracorporeal circulation and treatment of blood: Extracorporeal liver support (dialysis) |

| Surgical treatment |  |
| --- | --- |
| OPS code |  |
| 5 | Operations |

| Tracheostomy |  |
| --- | --- |
| OPS codes |  |
| 5-311 | Other larynx surgery and surgery on the trachea: Temporary tracheostomy |
| 5-312 | Other larynx surgery and surgery on the trachea: Permanent tracheostomy |

**Outcomes**

**Postsepsis morbidity**

| **Diagnoses in the medical domain** |  |
| --- | --- |
| Any pneumological diagnosis |  |
| ICD-10-GM codes |  |
| J96 | Respiratory failure, not elsewhere classified |
| R06.0 | Dyspnoea |
| J80 | Adult respiratory distress syndrome [ARDS] |
| R94.2 | Abnormal results of pulmonary function studies |
| J98 | Other respiratory disorders |
| R04.2 | Hemoptysis |
| U69.6! | Presence of a chronic idiopathic and/or chronic refractory cough |
| R09.3 | Abnormal sputum |
| R04.8 | Hemorrhage from other sites in respiratory passages |
| R04.9 | Hemorrhage from respiratory passages, unspecified |
| R05 | Cough |
| R06 | Abnormalities of breathing |
| R06.2 | Wheezing |
| R06.7 | Sneezing |
| J84 | Other interstitial pulmonary diseases |
| J95.9 | Postprocedural complications and disorders of respiratory system, unspecified |
| Z99.0 | Dependence on aspirator |
| Z99.1 | Dependence on respirator |
| Respiratory insufficiency, dyspnoea, respiratory distress syndrome |  |
| ICD-10-GM codes |  |
| J96 | Respiratory failure, not elsewhere classified |
| R06.0 | Dyspnoea |
| J80 | Adult respiratory distress syndrome [ARDS] |
| Abnormal lung function results |  |
| ICD-10-GM code |  |
| R94.2 | Abnormal results of pulmonary function studies |

| Cardiovascular diagnoses |  |
| --- | --- |
| ICD-10-GM codes |  |
| I20 | Angina pectoris |
| I21 | Acute myocardial infarction |
| I22 | Subsequent myocardial infarction |
| I24 | Other acute ischaemic heart diseases |
| I25 | Chronic ischemic heart disease |
| I42 | Cardiomyopathy |
| I51.4 | Myocarditis, unspecified |
| I50 | Heart failure |
| I47 | Paroxysmal tachycardia |
| I48 | Atrial fibrillation and flutter |
| I49 | Other cardiac arrhythmias |
| R00.0 | Tachycardia, unspecified |
| R00.2 | Palpitations |
| I95.1 | Orthostatic hypotension |
| I10 | Essential (primary) hypertension |
| I11 | Hypertensive heart disease |

| Renal diagnoses |  |
| --- | --- |
| ICD-10-GM codes |  |
| N10 | Acute tubulo-interstitial nephritis |
| N17 | Acute kidney failure |
| N18 | Chronic kidney disease (CKD) |
| N19 | Unspecified kidney failure |
| Z49 | Encounter for care involving renal dialysis |

| Urogynecological diagnoses |  |
| --- | --- |
| ICD-10-GM codes |  |
| R32 | Unspecified urinary incontinence |
| N39.3 | Stress incontinence |
| N39.4 | Other specified urinary incontinence |
| R15 | Fecal incontinence |
| N99.1 | Postprocedural urethral stricture |
| F52 | Sexual dysfunction, not caused by organic disorder or disease |
| N89.8 | Other specified noninflammatory disorders of vagina |
| N89.9 | Noninflammatory disorder of vagina, unspecified |
| N92 | Excessive, frequent or irregular menstruation |
| N95.1 | Menopausal and female climacteric states |
| R33 | Retention of urine |

| Metabolic diagnoses |  |
| --- | --- |
| ICD-10-GM codes |  |
| E11 | Type 2 diabetes mellitus |
| E12 | Malnutrition-related diabetes mellitus |
| E13 | Other specified diabetes mellitus |
| E14 | Unspecified diabetes mellitus |
| E27 | Other disorders of adrenal gland |
| E35* | Disorders of endocrine glands in diseases classified elsewhere |
| E34.9 | Endocrine disorder, unspecified |
| E23 | Hypofunction and other disorders of the pituitary gland |

| Anemia |  |
| --- | --- |
| ICD-10-GM codes |  |
| D50 | Iron deficiency anemia |
| D51 | Vitamin B12 deficiency anemia |
| D52 | Folate deficiency anemia |
| D53 | Other nutritional anemias |
| D63 | Anemia in chronic diseases classified elsewhere |
| D64.9 | Anemia, unspecified |

| Hepatic diagnoses |  |
| --- | --- |
| ICD-10-GM codes |  |
| K72.1 | Chronic hepatic failure |
| K76.0 | Fatty (change of) liver, not elsewhere classified |
| K83.01 | Secondary sclerosing cholangitis |
| K71 | Toxic liver disease |

| Gastroenterological diagnoses |  |
| --- | --- |
| ICD-10-GM codes |  |
| K12.0 | Recurrent oral aphthae |
| K52.9 | Noninfective gastroenteritis and colitis, unspecified |
| K59.09 | Other and unspecified constipation |
| K59.1 | Functional diarrhea |
| R11 | Nausea and vomiting |
| R19.4 | Change in bowel habit |
| A09 | Other infectious gastroenteritis and colitis, unspecified |
| K12.1 | Other forms of stomatitis |
| K29 | Gastritis and duodenitis |
| R14 | Flatulence and related conditions |
| K91 | Postprocedural complications and disorders of digestive system, not elsewhere classified |
| K58 | Irritable bowel syndrome |
| R12 | Heartburn |

| Any Ear, Nose and Throat (ENT) diagnosis |  |
| --- | --- |
| ICD-10-GM codes |  |
| R13 | Dysphagia |
| Z43.0 | Encounter for attention to tracheostomy |
| Z93.0 | Tracheostomy status |
| J95.0 | Tracheostomy complications |
| R43 | Disturbances of smell and taste |
| H93.1 | Tinnitus |
| R49 | Voice disturbances |
| J95.5 | Postprocedural subglottic stenosis |
| J95.81 | Tracheal stenosis following a procedure |
| J38.6 | Stenosis of larynx |
| J39.8 | Acquired tracheal stenosis |
| R42 | Dizziness and giddiness |
| H81 | Disorders of vestibular function |
| H90 | Conductive and sensorineural hearing loss |
| H91 | Other hearing loss |
| H93.0 | Degenerative and vascular disorders of ear |
| H93.2 | Other abnormal auditory perceptions |
| H93.3 | Disorders of acoustic nerve |
| H93.8 | Other specified disorders of ear |
| H93.9 | Unspecified disorder of ear |
| J31.0 | Chronic rhinitis |
| K11.7 | Disturbances of salivary secretion |
| Dysphagia |  |
| ICD-10-GM-code |  |
| R13 | Dysphagia |
| Tracheostoma-related diagnoses |  |
| ICD-10-GM codes |  |
| Z43.0 | Encounter for attention to tracheostomy |
| Z93.0 | Tracheostomy status |
| J95.0 | Tracheostomy complications |
| Taste and smelling disorders |  |
| ICD-10-GM code |  |
| R43 | Disturbances of smell and taste |
| Tinnitus |  |
| ICD-10-GM code |  |
| H93.1 | Tinnitus |

| Dermatological diagnoses |  |
| --- | --- |
| ICD-10-GM codes |  |
| L63 | Alopecia areata |
| L65 | Other nonscarring hair loss |
| L66 | Cicatricial alopecia [scarring hair loss] |
| L20 | Atopic (endogenous) eczema |
| L29 | Pruritus |
| L60 | Nail disorders |
| L85.3 | Xerosis cutis |
| R20 | Disturbances of skin sensation |
| R21 | Rash and other nonspecific skin eruption |
| R22 | Localized swelling, mass and lump of skin and subcutaneous tissue |
| R23.8 | Other and unspecified skin changes |
| R60 | Edema, not elsewhere classified |
| R61 | Hyperhidrosis |
| L89 | Decubitus ulcer and pressure area |

| Ophthalmological diagnoses |  |
| --- | --- |
| ICD-10-GM codes |  |
| H04.1 | Other disorders of lacrimal gland |
| H10 | Conjunctivitis |
| H53 | Visual disturbances |
| H54 | Blindness and visual impairment |

| Musculoskeletal diagnoses |  |
| --- | --- |
| ICD-10-GM codes |  |
| U50 | Motor function impairment |
| M60 | Myositis |
| M67 | Other disorders of synovium and tendon |
| Z89 | Acquired absence of limb |
| M62.4 | Contracture of muscle |
| M24.5 | Contracture of joint |
| M25.6 | Stiffness of joint, not elsewhere classified |
| M21.62 | Acquired Pes equinus |
| R26.2 | Difficulty in walking, not elsewhere classified |
| R26.3 | Immobility |
| R29.6 | Tendency to fall, not elsewhere classified |
| Z99.3 | Dependence on wheelchair |
| Z74.0 | Need for assistance due to reduced mobility |
| R25 | Abnormal involuntary movements |

| Neurological diagnoses |  |
| --- | --- |
| ICD-10-GM codes |  |
| I63 | Cerebral infarction |
| I64 | Stroke, not specified as haemorrhage or infarction |
| I65 | Occlusion and stenosis of precerebral arteries, not resulting in cerebral infarction |
| I66 | Occlusion and stenosis of cerebral arteries, not resulting in cerebral infarction |

| Intensive Care Unit-Acquired Weakness |  |
| --- | --- |
| ICD-10-GM codes |  |
| G62.8 | Critical illness polyneuropathy |
| G72.8 | Critical illness myopathy |
| G62 | Other polyneuropathies |
| G72 | Other and myopathies |

| Impairment of the general condition |  |
| --- | --- |
| Impairment of nutrition |  |
| ICD-10-GM codes |  |
| E41 | Nutritional marasmus |
| E43 | Unspecified severe protein-energy malnutrition |
| E44 | Protein-energy malnutrition of moderate and mild degree |
| E46 | Unspecified protein-energy malnutrition |
| R63.0 | Anorexia |
| R63.3 | Feeding difficulties and mismanagement |
| R63.4 | Abnormal weight loss |
| R63.6 | Insufficient intake of food and water |
| R63.8 | Other symptoms and signs concerning food and fluid intake |
| R64 | Cachexia |
| R63.1 | Polydipsia |
| Fatigue |  |
| ICD-10-GM codes |  |
| R53 | Malaise and fatigue |
| G93.3 | Chronic fatigue syndrome |
| (Chronic) pain |  |
| ICD-10-GM codes |  |
| R52 | Pain, not elsewhere classified |
| R10 | Abdominal and pelvic pain |
| R10.1 | Pain localized to upper abdomen |
| R10.4 | Other and unspecified abdominal pain |
| R52.1 | Chronic intractable pain |
| R52.2 | Other chronic pain |
| R52.9 | Pain unspecified |
| F45.4 | Persistent somatoform pain disorder |
| F45.41 | Chronic pain disorder associated with psychological and behavioural factors |
| G54.6 | Phantom limb syndrome with pain |
| R51 | Headache |
| G43 | Migraine |
| G44 | Other headache syndromes |
| R07 | Pain in throat and chest |
| R07.1 | Chest pain on breathing |
| R07.3 | Other chest pain |
| R07.4 | Chest pain, unspecified |
| M25.5 | Pain in joint |
| M79.1 | Myalgia |
| M54 | Dorsalgia |
| M79.6 | Pain in the extremities |
| M79.9 | Soft tissue disorder, unspecified |
| M79.2 | Neuralgia and neuritis, unspecified |

| **Diagnoses in the cognitive domain** |  |
| --- | --- |
| **Cognitive diagnoses** |  |
| ICD-10-GM codes |  |
| F06.7 | Mild cognitive disorder |
| U51 | Impairment of cognitive function |
| F00* | Dementia in Alzheimer disease |
| F01 | Vascular dementia |
| F02* | Dementia in other diseases classified elsewhere |
| F03 | Unspecified dementia |
| G30 | Alzheimer disease |
| R40.0 | Somnolence |
| R41.0 | Disorientation, unspecified |
| F04 | Organic amnesic syndrome, not induced by alcohol and other psychoactive substances |
| F05 | Delirium, not induced by alcohol and other psychoactive substances |
| F06.9 | Unspecified organic mental disorder due to brain damage or dysfunction or to physical disease |
| F07.8 | Other organic personality and behavioural disorders due to brain disease, damage or dysfunction |
| F07.9 | Unspecified organic personality and behavioural disorder due to brain disease, damage or dysfunction |
| G31.0 | Circumscribed brain atrophy |
| G31.1 | Senile degeneration of brain, not elsewhere classified |
| G31.9 | Degenerative disease of nervous system, unspecified |
| G32* | Other degenerative disorders of nervous system in diseases classified elsewhere |
| G93.4 | Encephalopathy, unspecified |
| (Mild) cognitive impairment |  |
| ICD-10-GM codes |  |
| F06.7 | Mild cognitive disorder |
| U51 | Impairment of cognitive function |
| Dementia |  |
| ICD-10-GM codes |  |
| F00* | Dementia in Alzheimer disease |
| F01 | Vascular dementia |
| F02* | Dementia in other diseases classified elsewhere |
| F03 | Unspecified dementia |
| G30 | Alzheimer disease |

| **Diagnoses in the psychological domain** |  |
| --- | --- |
| **Psychological diagnoses** |  |
| Other psychological diagnoses |  |
| ICD-10-GM codes |  |
| F06.8 | Other organic mental disorders due to brain damage or dysfunction or to physical disease |
| F06.9 | Unspecified organic mental disorder due to brain damage or dysfunction or to physical disease |
| F34 | Persistent mood [affective] disorders |
| F39 | Unspecified mood [affective] disorder |
| F44 | Dissociative disorders [conversion disorders] |
| F45 | Somatoform disorders |
| F48 | Other neurotic disorders |
| F30 | Manic episode |
| F31 | Bipolar disorder |
| R45.2 | Unhappiness |
| F07 | Personality and behavioral disorder due to brain disease, damage or dysfunction |
| F48.0 | Neurasthenia |
| F62 | Persistent personality disorders, not due to brain damage or disease |
| F23 | Brief psychotic disorder |
| Depression |  |
| ICD-10-GM codes |  |
| F32 | Depressive episode |
| F33 | Recurrent depressive disorder |
| F34.1 | Dysthymia |
| F38 | Other mood [affective] disorders |
| F41.2 | Mixed anxiety and depressive disorder |
| F06.3 | Organic mood [affective] disorders |
| Anxiety |  |
| ICD-10-GM codes |  |
| F40 | Phobic anxiety disorders |
| F41 | Other anxiety disorders |
| F06.4 | Organic anxiety disorder |
| Trauma- and stressor-related disorders, PTSD |  |
| ICD-10-GM codes |  |
| F43 | Reaction to severe stress, and adjustment disorders |
| F43.0 | Acute stress reaction |
| F43.1 | Post-traumatic stress disorder |
| F43.2 | Adjustment disorders |
| F43.8 | Other reactions to severe stress |
| F43.9 | Reaction to severe stress, unspecified |
| Sleep disorders |  |
| ICD-10-GM codes |  |
| F51 | Nonorganic sleep disorders |
| G47 | Sleep disorders |
| G47.0 | Insomnia |
| G47.1 | Hypersomnia |
| G47.9 | Sleep disorder, unspecified |
| Substance use disorders |  |
| ICD-10-GM codes |  |
| F10 | Mental and behavioural disorders due to use of alcohol |
| F11 | Mental and behavioural disorders due to use of opioids |
| F12 | Mental and behavioural disorders due to use of cannabinoids |
| F13 | Mental and behavioural disorders due to use of sedatives or hypnotics |
| F14 | Mental and behavioural disorders due to use of cocaine |
| F15 | Mental and behavioural disorders due to use of other stimulants, including caffeine |
| F16 | Mental and behavioural disorders due to use of hallucinogens |
| F17 | Mental and behavioural disorders due to use of tobacco |
| F18 | Mental and behavioural disorders due to use of volatile solvents |
| F19 | Mental and behavioural disorders due to multiple drug use and use of other psychoactive substances |

| **Diagnoses without assignment to a domain** |  |
| --- | --- |
| Venous thromboembolism |  |
| ICD-10-GM codes |  |
| I80.1 | Thrombosis, phlebitis and thrombophlebitis of femoral vein |
| I80.2 | Thrombosis, phlebitis and thrombophlebitis of other deep vessels of lower extremities |
| I80.3 | Thrombosis, phlebitis and thrombophlebitis of lower extremities, unspecified |
| I80.8 | Thrombosis, phlebitis and thrombophlebitis of other sites |
| I80.9 | Thrombosis, phlebitis and thrombophlebitis of unspecified site |

**Covariates used for IPTW**

| **Pre-existing medication** |  |
| --- | --- |
| Pre-existing contraceptive medication |  |
| Anatomial Therapeutic Chemical code |  |
| G03 | Sex hormones and modulators of the genital system |
| Pre-existing anticoagulation |  |
| Anatomial Therapeutic Chemical codes |  |
| B01AA | Vitamin K antagonists |
| B01AB | Heparin group |
| B01AE | Direct thrombin inhibitors |
| B01AF | Direct factor Xa inhibitors |
| B01AX | Other antithrombotic agents |

| Long-Covid morbidity^1^ |  |
| --- | --- |
| Pre-existing disturbances of smell and taste |  |
| ICD-10-GM code |  |
| R43 | Disturbances of smell and taste |

| Pre-existing post-exertional malaise |  |
| --- | --- |
| ICD-10-GM codes |  |
| F48.0 | Neurasthenia |
| R53 | Malaise and fatigue |

| Pre-existing cough |  |
| --- | --- |
| ICD-10-GM code |  |
| R05 | Cough |

| Pre-existing brain fog |  |
| --- | --- |
| ICD-10-GM codes |  |
| F06.7 | Mild cognitive disorder |
| U51 | Impairment of cognitive function |

| Pre-existing polydipsia |  |
| --- | --- |
| ICD-10-GM code |  |
| R63.1 | Polydipsia |

| Pre-existing palpitations |  |
| --- | --- |
| ICD-10-GM code |  |
| R00.2 | Palpitations |

| Pre-existing chest pain |  |
| --- | --- |
| ICD-10-GM codes |  |
| R07.1 | Chest pain on breathing |
| R07.2 | Precordial pain |
| R07.3 | Other chest pain |
| R07.4 | Chest pain, unspecified |

| Pre-existing fatigue |  |
| --- | --- |
| ICD-10-GM code |  |
| G93.3 | Chronic fatigue syndrome |

| Pre-existing sexual dysfunction |  |
| --- | --- |
| ICD-10-GM code |  |
| F52 | Sexual dysfunction, not caused by organic disorder or disease |

| Pre-existing dizziness |  |
| --- | --- |
| ICD-10-GM code |  |
| R42 | Dizziness and giddiness |

| Pre-existing gastrointestinal disorders |  |
| --- | --- |
| ICD-10-GM codes |  |
| K59.09 | Other and unspecified constipation |
| K59.1 | Functional diarrhea |
| R11 | Nausea and vomiting |
| R19.4 | Change in bowel habit |
| R14 | Flatulence and related conditions |

| Pre-existing movement disorders | Pre-existing movement disorders |
| --- | --- |
| ICD-10-GM code |  |
| R25 | Abnormal involuntary movements |

| Pre-existing hair loss |  |
| --- | --- |
| ICD-10-GM codes |  |
| L63 | Alopecia areata |
| L65 | Other nonscarring hair loss |
| L66 | Cicatricial alopecia [scarring hair loss] |

Furthermore, pre-existing conditions according to Charlson and Elixhauser comorbidity indices ^2^ were included. If the ICD-10-GM codes of the two indices were identical, the Elixhauser comorbidity was selected,^3^ otherwise the comorbidity of the index with the most comprehensive selection of codes.

| **Charlson comorbidity index** |
| --- |
| Cerebrovascular disease |
| Dementia |
| Peptic ulcer disease |
| Renal disease |

| **Elixhauser comorbidity index** |
| --- |
| Alcohol abuse |
| Cardiac arrhythmias |
| Chronic pulmonary disease |
| Congestive heart failure |
| Depression |
| Diabetes, complicated |
| Diabetes, uncomplicated |
| Fluid and electrolyte disorders |
| Hypertension, complicated |
| Hypothyroidism |
| Liver disease |
| Lymphoma |
| Obesity |
| Other neurological disorders |
| Paralysis |
| Peripheral vascular disorders |
| Psychoses |
| Pulmonary circulation disorders |
| Rheumatic arthritis/ collagen vascular diseases |
| Solid tumor without metastasis |
| Valvular disease |
| Weight loss |

# **e-Figure 1. Covariate balance checks based on absolute standardized mean differences in sub-sample of N = 12854 sepsis survivors**


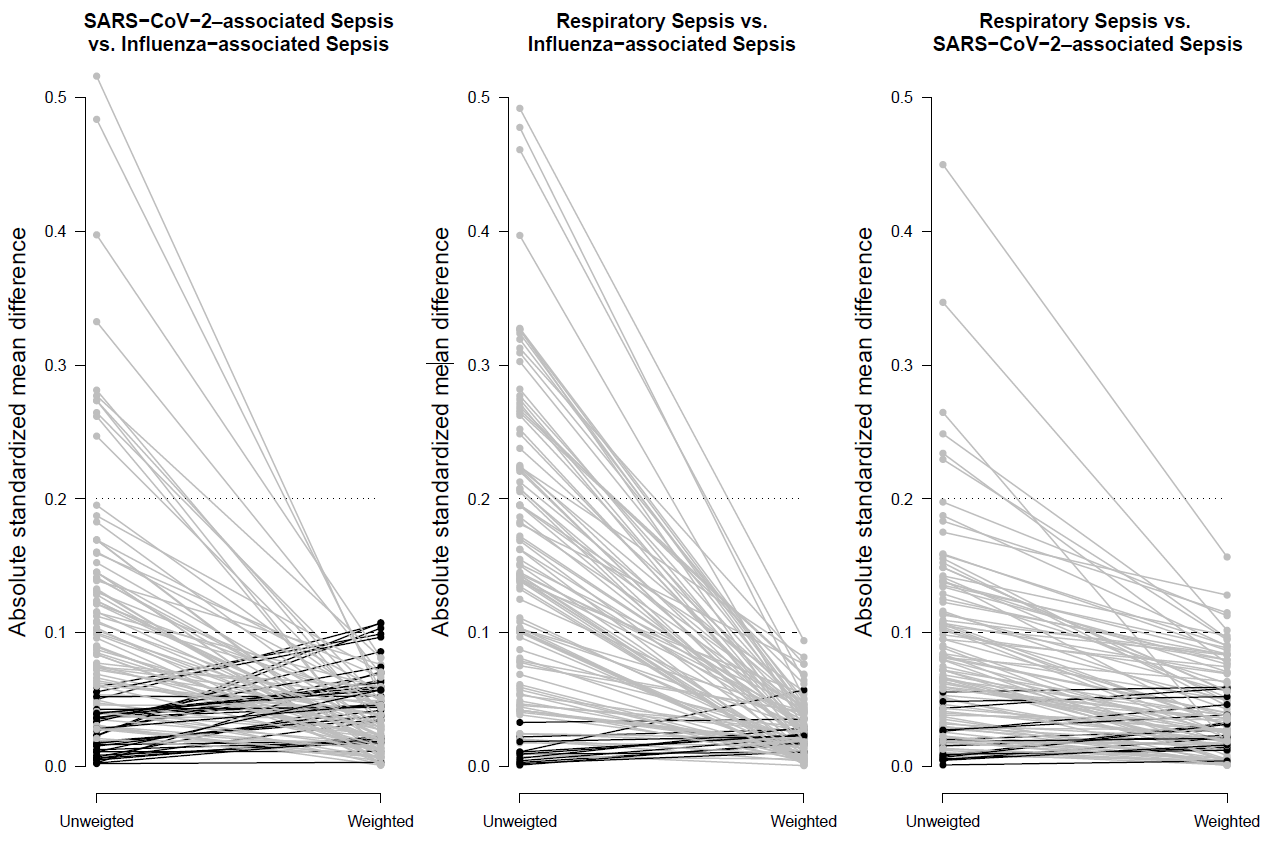


Pairwise unadjusted (e.g. unweighted) and adjusted (e.g. weighted IPTW) absolute standardized mean differences (ASMD) for the three groups of N = 12854 sepsis survivors

# **e-Table 1. Unadjusted (e.g. unweighted) and adjusted (e.g. weighted IPTW) means and absolute standardized mean differences (ASMD) between three groups of N = 12854 sepsis survivors (e.g., influenza-associated sepsis (group 1), SARS-CoV-2–associated sepsis (group 2), and respiratory sepsis (group 3))**

|  |  |  | Unadjusted | | |  | Adjusted (IPTW) | | | |
| --- | --- | --- | --- | --- | --- | --- | --- | --- | --- | --- |
| Variable | Contrast group *j* - group *k* |  | M group *k* | M group *j* | ASMD |  | M group *k* | M group *j* | ASMD | |
| Age | 2-1 |  | 65,567 | 63,723 | 0,128 |  | 66,205 | 66,304 | 0,007 | |
| Sex | 2-1 |  | 0,430 | 0,381 | 0,099 |  | 0,386 | 0,376 | 0,020 | |
| Infection of genitourinary system | 2-1 |  | 0,206 | 0,217 | 0,027 |  | 0,274 | 0,300 | 0,064 | |
| Wound and soft tissue infection | 2-1 |  | 0,054 | 0,036 | 0,084 |  | 0,073 | 0,075 | 0,006 | |
| Intraabdominal and retroperitoneal infection | 2-1 |  | 0,039 | 0,030 | 0,050 |  | 0,083 | 0,102 | 0,107 | |
| Intrathoracic infection | 2-1 |  | 0,015 | 0,012 | 0,023 |  | 0,013 | 0,025 | 0,108 | |
| Central nervous system infection | 2-1 |  | 0,007 | 0,005 | 0,035 |  | 0,006 | 0,011 | 0,059 | |
| Bloodstream and vascular infection | 2-1 |  | 0,055 | 0,051 | 0,019 |  | 0,077 | 0,085 | 0,035 | |
| Infection of bones, cartilage, joints | 2-1 |  | 0,017 | 0,005 | 0,124 |  | 0,018 | 0,015 | 0,023 | |
| Infection of the gastrointestinal tract and diarrhea | 2-1 |  | 0,094 | 0,086 | 0,031 |  | 0,106 | 0,105 | 0,001 | |
| Infections of the genital tract and sexually transmitted diseases | 2-1 |  | 0,010 | 0,007 | 0,037 |  | 0,008 | 0,012 | 0,045 | |
| Device-related infection | 2-1 |  | 0,049 | 0,042 | 0,037 |  | 0,065 | 0,073 | 0,036 | |
| Pre-existing inpatient nursing care | 2-1 |  | 0,052 | 0,046 | 0,027 |  | 0,052 | 0,067 | 0,069 | |
| Previous vaccination against influenza | 2-1 |  | 0,287 | 0,316 | 0,063 |  | 0,305 | 0,323 | 0,039 | |
| Pre-existing anticoagulation | 2-1 |  | 0,155 | 0,139 | 0,046 |  | 0,166 | 0,182 | 0,045 | |
| Pre-existing contraceptive medication | 2-1 |  | 0,013 | 0,014 | 0,007 |  | 0,007 | 0,013 | 0,049 | |
| Pre-existing number of outpatient treatments | 2-1 |  | 9,534 | 9,569 | 0,006 |  | 9,276 | 9,399 | 0,021 | |
| Pre-existing number of rehospitalizations | 2-1 |  | 1,279 | 0,778 | 0,273 |  | 1,175 | 1,249 | 0,041 | |
| Pre-existing immunosuppression incl. asplenia | 2-1 |  | 0,299 | 0,225 | 0,169 |  | 0,300 | 0,304 | 0,009 | |
| Pre-existing medical immunosuppression | 2-1 |  | 0,255 | 0,147 | 0,274 |  | 0,190 | 0,187 | 0,007 | |
| Pre-existing asplenia | 2-1 |  | 0,000 | 0,002 | 0,059 |  | 0,000 | 0,003 | 0,099 | |
| Prior organ transplantation | 2-1 |  | 0,015 | 0,010 | 0,040 |  | 0,015 | 0,012 | 0,025 | |
| Pre-existing infection/colonisation with multi-resistant pathogens | 2-1 |  | 0,026 | 0,015 | 0,077 |  | 0,023 | 0,030 | 0,053 | |
| Previously implanted foreign material | 2-1 |  | 0,200 | 0,191 | 0,023 |  | 0,211 | 0,252 | 0,103 | |
| Pre-existing mechanical ventilation | 2-1 |  | 0,102 | 0,032 | 0,281 |  | 0,085 | 0,077 | 0,030 | |
| Pre-existing long-term mechanical ventilation | 2-1 |  | 0,041 | 0,012 | 0,183 |  | 0,030 | 0,025 | 0,028 | |
| Pre-existing dialysis | 2-1 |  | 0,044 | 0,028 | 0,084 |  | 0,035 | 0,033 | 0,010 | |
| Pre-existing immobility | 2-1 |  | 0,193 | 0,141 | 0,141 |  | 0,199 | 0,210 | 0,029 | |
| Prior palliative treatment | 2-1 |  | 0,020 | 0,016 | 0,035 |  | 0,023 | 0,025 | 0,017 | |
| Any pre-existing pneumological diagnosis | 2-1 |  | 0,427 | 0,270 | 0,332 |  | 0,375 | 0,343 | 0,069 | |
| Pre-existing respiratory insufficiency, dyspnoea, respiratory distress syndrome | 2-1 |  | 0,329 | 0,162 | 0,397 |  | 0,278 | 0,244 | 0,081 | |
| Prior abnormal lung function results | 2-1 |  | 0,032 | 0,020 | 0,074 |  | 0,023 | 0,019 | 0,028 | |
| Pre-existing cardiovascular diagnoses | 2-1 |  | 0,792 | 0,744 | 0,116 |  | 0,793 | 0,787 | 0,016 | |
| Pre-existing renal diagnoses | 2-1 |  | 0,305 | 0,242 | 0,141 |  | 0,305 | 0,313 | 0,018 | |
| Pre-existing metabolic diagnoses | 2-1 |  | 0,421 | 0,426 | 0,011 |  | 0,426 | 0,431 | 0,011 | |
| Pre-existing anemia | 2-1 |  | 0,176 | 0,128 | 0,132 |  | 0,179 | 0,181 | 0,004 | |
| Pre-existing hepatic diagnoses | 2-1 |  | 0,116 | 0,118 | 0,006 |  | 0,109 | 0,120 | 0,034 | |
| Pre-existing gastroenterological diagnoses | 2-1 |  | 0,292 | 0,307 | 0,033 |  | 0,292 | 0,332 | 0,086 | |
| Any pre-existing ENT diagnosis | 2-1 |  | 0,253 | 0,307 | 0,122 |  | 0,290 | 0,311 | 0,045 | |
| Pre-existing dysphagia | 2-1 |  | 0,036 | 0,029 | 0,039 |  | 0,044 | 0,053 | 0,046 | |
| Pre-existing tracheostoma-related diagnoses | 2-1 |  | 0,004 | 0,005 | 0,003 |  | 0,006 | 0,010 | 0,071 | |
| Pre-existing taste and smelling disorders | 2-1 |  | 0,004 | 0,003 | 0,017 |  | 0,003 | 0,006 | 0,038 | |
| Pre-existing tinnitus | 2-1 |  | 0,028 | 0,054 | 0,133 |  | 0,038 | 0,038 | 0,002 | |
| Pre-existing dermatological diagnoses | 2-1 |  | 0,241 | 0,220 | 0,050 |  | 0,246 | 0,247 | 0,002 | |
| Pre-existing urogynecological diagnoses | 2-1 |  | 0,277 | 0,264 | 0,031 |  | 0,290 | 0,288 | 0,004 | |
| Pre-existing ophthalmological diagnoses | 2-1 |  | 0,170 | 0,198 | 0,072 |  | 0,169 | 0,184 | 0,039 | |
| Pre-existing musculoskeletal diagnoses | 2-1 |  | 0,303 | 0,229 | 0,169 |  | 0,312 | 0,315 | 0,007 | |
| Pre-existing neurological diagnoses | 2-1 |  | 0,120 | 0,099 | 0,069 |  | 0,126 | 0,133 | 0,022 | |
| Pre-existing Intensive Care Unit-Acquired Weakness | 2-1 |  | 0,118 | 0,090 | 0,090 |  | 0,113 | 0,116 | 0,009 | |
| Pre-existing cognitive diagnoses | 2-1 |  | 0,173 | 0,131 | 0,115 |  | 0,177 | 0,198 | 0,059 | |
| Pre-existing (mild) cognitive impairment | 2-1 |  | 0,065 | 0,045 | 0,090 |  | 0,071 | 0,068 | 0,011 | |
| Pre-existing dementia | 2-1 |  | 0,084 | 0,070 | 0,054 |  | 0,085 | 0,098 | 0,049 | |
| Pre-existing psychological diagnoses | 2-1 |  | 0,653 | 0,518 | 0,277 |  | 0,615 | 0,575 | 0,081 | |
| Other pre-existing psychological diagnoses | 2-1 |  | 0,229 | 0,227 | 0,005 |  | 0,216 | 0,226 | 0,022 | |
| Pre-existing depression | 2-1 |  | 0,305 | 0,275 | 0,066 |  | 0,286 | 0,277 | 0,021 | |
| Pre-existing anxiety | 2-1 |  | 0,126 | 0,096 | 0,096 |  | 0,098 | 0,097 | 0,003 | |
| Pre-existing trauma- and stressor-related disorders, PTSD | 2-1 |  | 0,091 | 0,077 | 0,052 |  | 0,086 | 0,072 | 0,053 | |
| Pre-existing sleep disorders | 2-1 |  | 0,187 | 0,181 | 0,016 |  | 0,179 | 0,187 | 0,019 | |
| Pre-existing substance use disorders | 2-1 |  | 0,313 | 0,109 | 0,516 |  | 0,252 | 0,231 | 0,052 | |
| Pre-existing impairment of the general condition | 2-1 |  | 0,611 | 0,665 | 0,112 |  | 0,605 | 0,630 | 0,051 | |
| Pre-existing impairment of nutrition | 2-1 |  | 0,070 | 0,038 | 0,139 |  | 0,061 | 0,078 | 0,076 | |
| Pre-existing fatigue | 2-1 |  | 0,070 | 0,058 | 0,048 |  | 0,060 | 0,065 | 0,021 | |
| Pre-existing (chronic) pain | 2-1 |  | 0,565 | 0,647 | 0,169 |  | 0,566 | 0,593 | 0,056 | |
| Pre-existing medical diagnoses | 2-1 |  | 0,955 | 0,949 | 0,028 |  | 0,949 | 0,951 | 0,008 | |
| Pre-existing venous thromboembolism | 2-1 |  | 0,019 | 0,027 | 0,056 |  | 0,020 | 0,035 | 0,097 | |
| Pre-existing hair loss | 2-1 |  | 0,009 | 0,011 | 0,019 |  | 0,006 | 0,008 | 0,019 | |
| Pre-existing disturbances of smell and taste | 2-1 |  | 0,004 | 0,003 | 0,017 |  | 0,003 | 0,006 | 0,038 | |
| Pre-existing post-exertional malaise | 2-1 |  | 0,084 | 0,076 | 0,030 |  | 0,074 | 0,078 | 0,014 | |
| Pre-existing cough | 2-1 |  | 0,060 | 0,060 | 0,002 |  | 0,059 | 0,059 | 0,003 | |
| Pre-existing brain fog | 2-1 |  | 0,065 | 0,045 | 0,090 |  | 0,071 | 0,068 | 0,011 | |
| Pre-existing polydipsia | 2-1 |  | 0,001 | 0,001 | 0,013 |  | 0,001 | 0,001 | 0,019 | |
| Pre-existing palpitations | 2-1 |  | 0,007 | 0,007 | 0,008 |  | 0,007 | 0,005 | 0,018 | |
| Pre-existing chest pain | 2-1 |  | 0,057 | 0,062 | 0,021 |  | 0,048 | 0,050 | 0,007 | |
| Pre-existing fatigue | 2-1 |  | 0,003 | 0,004 | 0,015 |  | 0,002 | 0,005 | 0,042 | |
| Pre-existing sexual dysfunction | 2-1 |  | 0,038 | 0,046 | 0,042 |  | 0,037 | 0,038 | 0,007 | |
| Pre-existing dizziness | 2-1 |  | 0,089 | 0,122 | 0,109 |  | 0,113 | 0,118 | 0,019 | |
| Pre-existing gastrointestinal disorders | 2-1 |  | 0,113 | 0,101 | 0,040 |  | 0,110 | 0,129 | 0,060 | |
| Pre-existing movement disorders | 2-1 |  | 0,032 | 0,027 | 0,029 |  | 0,031 | 0,030 | 0,007 | |
| Charlson: Cerebrovascular disease | 2-1 |  | 0,189 | 0,175 | 0,036 |  | 0,198 | 0,226 | 0,074 | |
| Charlson: Dementia | 2-1 |  | 0,089 | 0,071 | 0,064 |  | 0,091 | 0,099 | 0,030 | |
| Charlson: Peptic ulcer disease | 2-1 |  | 0,028 | 0,018 | 0,061 |  | 0,024 | 0,030 | 0,039 | |
| Charlson: Renal disease | 2-1 |  | 0,287 | 0,234 | 0,122 |  | 0,286 | 0,296 | 0,023 | |
| Elixhauser: Alcohol abuse | 2-1 |  | 0,109 | 0,041 | 0,262 |  | 0,094 | 0,096 | 0,009 | |
| Elixhauser: Cardiac arrhythmias | 2-1 |  | 0,293 | 0,227 | 0,152 |  | 0,302 | 0,288 | 0,031 | |
| Elixhauser: Chronic pulmonary disease | 2-1 |  | 0,512 | 0,282 | 0,484 |  | 0,371 | 0,344 | 0,057 | |
| Elixhauser: Congestive heart failure | 2-1 |  | 0,335 | 0,218 | 0,265 |  | 0,325 | 0,303 | 0,050 | |
| Elixhauser: Depression | 2-1 |  | 0,318 | 0,290 | 0,060 |  | 0,299 | 0,292 | 0,014 | |
| Elixhauser: Diabetes, complicated | 2-1 |  | 0,250 | 0,251 | 0,003 |  | 0,260 | 0,269 | 0,021 | |
| Elixhauser: Diabetes, uncomplicated | 2-1 |  | 0,380 | 0,401 | 0,043 |  | 0,385 | 0,406 | 0,044 | |
| Elixhauser: Fluid and electrolyte disorders | 2-1 |  | 0,235 | 0,140 | 0,247 |  | 0,230 | 0,243 | 0,035 | |
| Elixhauser: Hypertension, complicated | 2-1 |  | 0,215 | 0,184 | 0,076 |  | 0,208 | 0,202 | 0,015 | |
| Elixhauser: Hypothyroidism | 2-1 |  | 0,157 | 0,170 | 0,037 |  | 0,149 | 0,173 | 0,063 | |
| Elixhauser: Liver disease | 2-1 |  | 0,210 | 0,177 | 0,085 |  | 0,214 | 0,207 | 0,019 | |
| Elixhauser: Lymphoma | 2-1 |  | 0,020 | 0,011 | 0,074 |  | 0,019 | 0,013 | 0,046 | |
| Elixhauser: Obesity | 2-1 |  | 0,306 | 0,355 | 0,104 |  | 0,319 | 0,320 | 0,001 | |
| Elixhauser: Other neurological disorders | 2-1 |  | 0,118 | 0,075 | 0,145 |  | 0,109 | 0,117 | 0,026 | |
| Elixhauser: Paralysis | 2-1 |  | 0,074 | 0,056 | 0,074 |  | 0,076 | 0,090 | 0,060 | |
| Elixhauser: Peripheral vascular disorders | 2-1 |  | 0,251 | 0,191 | 0,145 |  | 0,260 | 0,261 | 0,001 | |
| Elixhauser: Psychoses | 2-1 |  | 0,067 | 0,032 | 0,159 |  | 0,058 | 0,043 | 0,067 | |
| Elixhauser: Pulmonary circulation disorders | 2-1 |  | 0,074 | 0,040 | 0,145 |  | 0,063 | 0,063 | 0,002 | |
| Elixhauser: Rheumatic arthritis/ collagen vascular diseases | 2-1 |  | 0,089 | 0,077 | 0,041 |  | 0,085 | 0,081 | 0,014 | |
| Elixhauser: Solid tumor without metastasis | 2-1 |  | 0,134 | 0,101 | 0,100 |  | 0,136 | 0,145 | 0,030 | |
| Elixhauser: Valvular disease | 2-1 |  | 0,174 | 0,137 | 0,102 |  | 0,169 | 0,176 | 0,020 | |
| Elixhauser: Weight loss | 2-1 |  | 0,060 | 0,027 | 0,160 |  | 0,052 | 0,062 | 0,049 | |
| No pre-existing nursing care dependency | 2-1 |  | 0,672 | 0,756 | 0,187 |  | 0,687 | 0,656 | 0,071 | |
| Prior nursing care level 1 | 2-1 |  | 0,052 | 0,028 | 0,124 |  | 0,030 | 0,035 | 0,022 | |
| Prior nursing care level 2 | 2-1 |  | 0,107 | 0,095 | 0,040 |  | 0,114 | 0,131 | 0,057 | |
| Prior nursing care level 3 | 2-1 |  | 0,097 | 0,073 | 0,087 |  | 0,098 | 0,106 | 0,028 | |
| Prior nursing care level 4 | 2-1 |  | 0,042 | 0,037 | 0,028 |  | 0,043 | 0,052 | 0,046 | |
| Prior nursing care level 5 | 2-1 |  | 0,029 | 0,011 | 0,130 |  | 0,027 | 0,021 | 0,046 | |
| Age | 3-1 |  | 66,696 | 63,723 | 0,208 |  | 66,153 | 66,304 | 0,011 | |
| Sex | 3-1 |  | 0,355 | 0,381 | 0,053 |  | 0,364 | 0,376 | 0,025 | |
| Infection of genitourinary system | 3-1 |  | 0,360 | 0,217 | 0,319 |  | 0,319 | 0,300 | 0,042 | |
| Wound and soft tissue infection | 3-1 |  | 0,119 | 0,036 | 0,313 |  | 0,095 | 0,075 | 0,077 | |
| Intraabdominal and retroperitoneal infection | 3-1 |  | 0,175 | 0,030 | 0,492 |  | 0,130 | 0,102 | 0,094 | |
| Intrathoracic infection | 3-1 |  | 0,044 | 0,012 | 0,196 |  | 0,034 | 0,025 | 0,056 | |
| Central nervous system infection | 3-1 |  | 0,020 | 0,005 | 0,144 |  | 0,016 | 0,011 | 0,043 | |
| Bloodstream and vascular infection | 3-1 |  | 0,126 | 0,051 | 0,266 |  | 0,104 | 0,085 | 0,069 | |
| Infection of bones, cartilage, joints | 3-1 |  | 0,036 | 0,005 | 0,224 |  | 0,027 | 0,015 | 0,082 | |
| Infection of the gastrointestinal tract and diarrhea | 3-1 |  | 0,114 | 0,086 | 0,097 |  | 0,107 | 0,105 | 0,005 | |
| Infections of the genital tract and sexually transmitted diseases | 3-1 |  | 0,024 | 0,007 | 0,138 |  | 0,019 | 0,012 | 0,054 | |
| Device-related infection | 3-1 |  | 0,111 | 0,042 | 0,265 |  | 0,091 | 0,073 | 0,068 | |
| Pre-existing inpatient nursing care | 3-1 |  | 0,073 | 0,046 | 0,111 |  | 0,064 | 0,067 | 0,013 | |
| Previous vaccination against influenza | 3-1 |  | 0,334 | 0,316 | 0,039 |  | 0,329 | 0,323 | 0,011 | |
| Pre-existing anticoagulation | 3-1 |  | 0,200 | 0,139 | 0,162 |  | 0,182 | 0,182 | 0,001 | |
| Pre-existing contraceptive medication | 3-1 |  | 0,009 | 0,014 | 0,047 |  | 0,010 | 0,013 | 0,026 | |
| Pre-existing number of outpatient treatments | 3-1 |  | 9,326 | 9,569 | 0,042 |  | 9,307 | 9,399 | 0,016 | |
| Pre-existing number of rehospitalizations | 3-1 |  | 1,613 | 0,778 | 0,461 |  | 1,366 | 1,249 | 0,064 | |
| Pre-existing immunosuppression incl. asplenia | 3-1 |  | 0,348 | 0,225 | 0,274 |  | 0,314 | 0,304 | 0,024 | |
| Pre-existing medical immunosuppression | 3-1 |  | 0,212 | 0,147 | 0,170 |  | 0,199 | 0,187 | 0,031 | |
| Pre-existing asplenia | 3-1 |  | 0,005 | 0,002 | 0,049 |  | 0,004 | 0,003 | 0,011 | |
| Prior organ transplantation | 3-1 |  | 0,012 | 0,010 | 0,020 |  | 0,011 | 0,012 | 0,004 | |
| Pre-existing infection/colonisation with multi-resistant pathogens | 3-1 |  | 0,044 | 0,015 | 0,172 |  | 0,035 | 0,030 | 0,031 | |
| Previously implanted foreign material | 3-1 |  | 0,285 | 0,191 | 0,221 |  | 0,259 | 0,252 | 0,017 | |
| Pre-existing mechanical ventilation | 3-1 |  | 0,111 | 0,032 | 0,309 |  | 0,090 | 0,077 | 0,050 | |
| Pre-existing long-term mechanical ventilation | 3-1 |  | 0,032 | 0,012 | 0,140 |  | 0,026 | 0,025 | 0,007 | |
| Pre-existing dialysis | 3-1 |  | 0,032 | 0,028 | 0,025 |  | 0,030 | 0,033 | 0,017 | |
| Pre-existing immobility | 3-1 |  | 0,252 | 0,141 | 0,282 |  | 0,221 | 0,210 | 0,027 | |
| Prior palliative treatment | 3-1 |  | 0,031 | 0,016 | 0,101 |  | 0,026 | 0,025 | 0,004 | |
| Any pre-existing pneumological diagnosis | 3-1 |  | 0,386 | 0,270 | 0,249 |  | 0,355 | 0,343 | 0,027 | |
| Pre-existing respiratory insufficiency, dyspnoea, respiratory distress syndrome | 3-1 |  | 0,298 | 0,162 | 0,327 |  | 0,263 | 0,244 | 0,047 | |
| Prior abnormal lung function results | 3-1 |  | 0,023 | 0,020 | 0,022 |  | 0,023 | 0,019 | 0,028 | |
| Pre-existing cardiovascular diagnoses | 3-1 |  | 0,799 | 0,744 | 0,133 |  | 0,789 | 0,787 | 0,005 | |
| Pre-existing renal diagnoses | 3-1 |  | 0,334 | 0,242 | 0,205 |  | 0,311 | 0,313 | 0,004 | |
| Pre-existing metabolic diagnoses | 3-1 |  | 0,425 | 0,426 | 0,003 |  | 0,423 | 0,431 | 0,017 | |
| Pre-existing anemia | 3-1 |  | 0,211 | 0,128 | 0,222 |  | 0,186 | 0,181 | 0,014 | |
| Pre-existing hepatic diagnoses | 3-1 |  | 0,121 | 0,118 | 0,009 |  | 0,117 | 0,120 | 0,011 | |
| Pre-existing gastroenterological diagnoses | 3-1 |  | 0,355 | 0,307 | 0,102 |  | 0,334 | 0,332 | 0,004 | |
| Any pre-existing ENT diagnosis | 3-1 |  | 0,315 | 0,307 | 0,018 |  | 0,303 | 0,311 | 0,016 | |
| Pre-existing dysphagia | 3-1 |  | 0,070 | 0,029 | 0,187 |  | 0,057 | 0,053 | 0,020 | |
| Pre-existing tracheostoma-related diagnoses | 3-1 |  | 0,027 | 0,005 | 0,181 |  | 0,020 | 0,010 | 0,077 | |
| Pre-existing taste and smelling disorders | 3-1 |  | 0,003 | 0,003 | 0,011 |  | 0,003 | 0,006 | 0,057 | |
| Pre-existing tinnitus | 3-1 |  | 0,029 | 0,054 | 0,125 |  | 0,032 | 0,038 | 0,030 | |
| Pre-existing dermatological diagnoses | 3-1 |  | 0,283 | 0,220 | 0,145 |  | 0,263 | 0,247 | 0,037 | |
| Pre-existing urogynecological diagnoses | 3-1 |  | 0,300 | 0,264 | 0,081 |  | 0,283 | 0,288 | 0,010 | |
| Pre-existing ophthalmological diagnoses | 3-1 |  | 0,168 | 0,198 | 0,078 |  | 0,174 | 0,184 | 0,025 | |
| Pre-existing musculoskeletal diagnoses | 3-1 |  | 0,377 | 0,229 | 0,326 |  | 0,335 | 0,315 | 0,043 | |
| Pre-existing neurological diagnoses | 3-1 |  | 0,145 | 0,099 | 0,141 |  | 0,132 | 0,133 | 0,003 | |
| Pre-existing Intensive Care Unit-Acquired Weakness | 3-1 |  | 0,121 | 0,090 | 0,101 |  | 0,113 | 0,116 | 0,008 | |
| Pre-existing cognitive diagnoses | 3-1 |  | 0,249 | 0,131 | 0,303 |  | 0,214 | 0,198 | 0,040 | |
| Pre-existing (mild) cognitive impairment | 3-1 |  | 0,094 | 0,045 | 0,195 |  | 0,079 | 0,068 | 0,044 | |
| Pre-existing dementia | 3-1 |  | 0,108 | 0,070 | 0,135 |  | 0,096 | 0,098 | 0,005 | |
| Pre-existing psychological diagnoses | 3-1 |  | 0,628 | 0,518 | 0,223 |  | 0,597 | 0,575 | 0,045 | |
| Other pre-existing psychological diagnoses | 3-1 |  | 0,231 | 0,227 | 0,010 |  | 0,225 | 0,226 | 0,001 | |
| Pre-existing depression | 3-1 |  | 0,293 | 0,275 | 0,041 |  | 0,285 | 0,277 | 0,018 | |
| Pre-existing anxiety | 3-1 |  | 0,098 | 0,096 | 0,007 |  | 0,095 | 0,097 | 0,006 | |
| Pre-existing trauma- and stressor-related disorders, PTSD | 3-1 |  | 0,086 | 0,077 | 0,033 |  | 0,082 | 0,072 | 0,035 | |
| Pre-existing sleep disorders | 3-1 |  | 0,183 | 0,181 | 0,004 |  | 0,178 | 0,187 | 0,021 | |
| Pre-existing substance use disorders | 3-1 |  | 0,296 | 0,109 | 0,478 |  | 0,251 | 0,231 | 0,051 | |
| Pre-existing impairment of the general condition | 3-1 |  | 0,632 | 0,665 | 0,069 |  | 0,634 | 0,630 | 0,008 | |
| Pre-existing impairment of nutrition | 3-1 |  | 0,110 | 0,038 | 0,277 |  | 0,089 | 0,078 | 0,041 | |
| Pre-existing fatigue | 3-1 |  | 0,085 | 0,058 | 0,103 |  | 0,076 | 0,065 | 0,041 | |
| Pre-existing (chronic) pain | 3-1 |  | 0,578 | 0,647 | 0,143 |  | 0,589 | 0,593 | 0,009 | |
| Pre-existing medical diagnoses | 3-1 |  | 0,949 | 0,949 | 0,002 |  | 0,948 | 0,951 | 0,011 | |
| Pre-existing venous thromboembolism | 3-1 |  | 0,037 | 0,027 | 0,054 |  | 0,034 | 0,035 | 0,004 | |
| Pre-existing hair loss | 3-1 |  | 0,007 | 0,011 | 0,042 |  | 0,006 | 0,008 | 0,017 | |
| Pre-existing disturbances of smell and taste | 3-1 |  | 0,003 | 0,003 | 0,011 |  | 0,003 | 0,006 | 0,057 | |
| Pre-existing post-exertional malaise | 3-1 |  | 0,098 | 0,076 | 0,079 |  | 0,089 | 0,078 | 0,040 | |
| Pre-existing cough | 3-1 |  | 0,050 | 0,060 | 0,046 |  | 0,052 | 0,059 | 0,030 | |
| Pre-existing brain fog | 3-1 |  | 0,094 | 0,045 | 0,195 |  | 0,079 | 0,068 | 0,044 | |
| Pre-existing polydipsia | 3-1 |  | 0,001 | 0,001 | 0,001 |  | 0,001 | 0,001 | 0,018 | |
| Pre-existing palpitations | 3-1 |  | 0,005 | 0,007 | 0,021 |  | 0,005 | 0,005 | 0,009 | |
| Pre-existing chest pain | 3-1 |  | 0,042 | 0,062 | 0,087 |  | 0,046 | 0,050 | 0,019 | |
| Pre-existing fatigue | 3-1 |  | 0,003 | 0,004 | 0,006 |  | 0,003 | 0,005 | 0,029 | |
| Pre-existing sexual dysfunction | 3-1 |  | 0,034 | 0,046 | 0,061 |  | 0,036 | 0,038 | 0,008 | |
| Pre-existing dizziness | 3-1 |  | 0,114 | 0,122 | 0,025 |  | 0,112 | 0,118 | 0,021 | |
| Pre-existing gastrointestinal disorders | 3-1 |  | 0,155 | 0,101 | 0,162 |  | 0,139 | 0,129 | 0,029 | |
| Pre-existing movement disorders | 3-1 |  | 0,034 | 0,027 | 0,042 |  | 0,033 | 0,030 | 0,014 | |
| Charlson: Cerebrovascular disease | 3-1 |  | 0,254 | 0,175 | 0,195 |  | 0,234 | 0,226 | 0,018 | |
| Charlson: Dementia | 3-1 |  | 0,111 | 0,071 | 0,139 |  | 0,099 | 0,099 | 0,001 | |
| Charlson: Peptic ulcer disease | 3-1 |  | 0,043 | 0,018 | 0,141 |  | 0,036 | 0,030 | 0,034 | |
| Charlson: Renal disease | 3-1 |  | 0,303 | 0,234 | 0,155 |  | 0,285 | 0,296 | 0,025 | |
| Elixhauser: Alcohol abuse | 3-1 |  | 0,130 | 0,041 | 0,324 |  | 0,106 | 0,096 | 0,036 | |
| Elixhauser: Cardiac arrhythmias | 3-1 |  | 0,327 | 0,227 | 0,225 |  | 0,299 | 0,288 | 0,025 | |
| Elixhauser: Chronic pulmonary disease | 3-1 |  | 0,382 | 0,282 | 0,213 |  | 0,363 | 0,344 | 0,039 | |
| Elixhauser: Congestive heart failure | 3-1 |  | 0,338 | 0,218 | 0,271 |  | 0,310 | 0,303 | 0,016 | |
| Elixhauser: Depression | 3-1 |  | 0,309 | 0,290 | 0,042 |  | 0,301 | 0,292 | 0,019 | |
| Elixhauser: Diabetes, complicated | 3-1 |  | 0,277 | 0,251 | 0,059 |  | 0,271 | 0,269 | 0,006 | |
| Elixhauser: Diabetes, uncomplicated | 3-1 |  | 0,396 | 0,401 | 0,011 |  | 0,395 | 0,406 | 0,024 | |
| Elixhauser: Fluid and electrolyte disorders | 3-1 |  | 0,301 | 0,140 | 0,397 |  | 0,255 | 0,243 | 0,028 | |
| Elixhauser: Hypertension, complicated | 3-1 |  | 0,207 | 0,184 | 0,057 |  | 0,200 | 0,202 | 0,006 | |
| Elixhauser: Hypothyroidism | 3-1 |  | 0,167 | 0,170 | 0,009 |  | 0,164 | 0,173 | 0,024 | |
| Elixhauser: Liver disease | 3-1 |  | 0,218 | 0,177 | 0,104 |  | 0,208 | 0,207 | 0,002 | |
| Elixhauser: Lymphoma | 3-1 |  | 0,020 | 0,011 | 0,075 |  | 0,018 | 0,013 | 0,041 | |
| Elixhauser: Obesity | 3-1 |  | 0,304 | 0,355 | 0,108 |  | 0,312 | 0,320 | 0,017 | |
| Elixhauser: Other neurological disorders | 3-1 |  | 0,161 | 0,075 | 0,269 |  | 0,137 | 0,117 | 0,062 | |
| Elixhauser: Paralysis | 3-1 |  | 0,113 | 0,056 | 0,207 |  | 0,098 | 0,090 | 0,027 | |
| Elixhauser: Peripheral vascular disorders | 3-1 |  | 0,299 | 0,191 | 0,252 |  | 0,271 | 0,261 | 0,024 | |
| Elixhauser: Psychoses | 3-1 |  | 0,053 | 0,032 | 0,103 |  | 0,049 | 0,043 | 0,029 | |
| Elixhauser: Pulmonary circulation disorders | 3-1 |  | 0,084 | 0,040 | 0,183 |  | 0,073 | 0,063 | 0,041 | |
| Elixhauser: Rheumatic arthritis/ collagen vascular diseases | 3-1 |  | 0,072 | 0,077 | 0,019 |  | 0,075 | 0,081 | 0,023 | |
| Elixhauser: Solid tumor without metastasis | 3-1 |  | 0,184 | 0,101 | 0,238 |  | 0,162 | 0,145 | 0,047 | |
| Elixhauser: Valvular disease | 3-1 |  | 0,190 | 0,137 | 0,143 |  | 0,177 | 0,176 | 0,002 | |
| Elixhauser: Weight loss | 3-1 |  | 0,087 | 0,027 | 0,262 |  | 0,070 | 0,062 | 0,036 | |
| No pre-existing nursing care dependency | 3-1 |  | 0,606 | 0,756 | 0,327 |  | 0,647 | 0,656 | 0,018 | |
| Prior nursing care level 1 | 3-1 |  | 0,036 | 0,028 | 0,045 |  | 0,035 | 0,035 | 0,005 | |
| Prior nursing care level 2 | 3-1 |  | 0,151 | 0,095 | 0,169 |  | 0,134 | 0,131 | 0,011 | |
| Prior nursing care level 3 | 3-1 |  | 0,117 | 0,073 | 0,150 |  | 0,104 | 0,106 | 0,006 | |
| Prior nursing care level 4 | 3-1 |  | 0,058 | 0,037 | 0,100 |  | 0,052 | 0,052 | 0,003 | |
| Prior nursing care level 5 | 3-1 |  | 0,033 | 0,011 | 0,151 |  | 0,027 | 0,021 | 0,046 | |
| Age | 3-2 |  | 66,696 | 65,567 | 0,080 |  | 66,153 | 66,205 | 0,004 | |
| Sex | 3-2 |  | 0,355 | 0,430 | 0,152 |  | 0,364 | 0,386 | 0,045 | |
| Infection of genitourinary system | 3-2 |  | 0,360 | 0,206 | 0,347 |  | 0,319 | 0,274 | 0,102 | |
| Wound and soft tissue infection | 3-2 |  | 0,119 | 0,054 | 0,234 |  | 0,095 | 0,073 | 0,077 | |
| Intraabdominal and retroperitoneal infection | 3-2 |  | 0,175 | 0,039 | 0,450 |  | 0,130 | 0,083 | 0,157 | |
| Intrathoracic infection | 3-2 |  | 0,044 | 0,015 | 0,175 |  | 0,034 | 0,013 | 0,128 | |
| Central nervous system infection | 3-2 |  | 0,020 | 0,007 | 0,113 |  | 0,016 | 0,006 | 0,080 | |
| Bloodstream and vascular infection | 3-2 |  | 0,126 | 0,055 | 0,249 |  | 0,104 | 0,077 | 0,096 | |
| Infection of bones, cartilage, joints | 3-2 |  | 0,036 | 0,017 | 0,115 |  | 0,027 | 0,018 | 0,056 | |
| Infection of the gastrointestinal tract and diarrhea | 3-2 |  | 0,114 | 0,094 | 0,066 |  | 0,107 | 0,106 | 0,004 | |
| Infections of the genital tract and sexually transmitted diseases | 3-2 |  | 0,024 | 0,010 | 0,105 |  | 0,019 | 0,008 | 0,083 | |
| Device-related infection | 3-2 |  | 0,111 | 0,049 | 0,230 |  | 0,091 | 0,065 | 0,095 | |
| Pre-existing inpatient nursing care | 3-2 |  | 0,073 | 0,052 | 0,084 |  | 0,064 | 0,052 | 0,050 | |
| Previous vaccination against influenza | 3-2 |  | 0,334 | 0,287 | 0,101 |  | 0,329 | 0,305 | 0,050 | |
| Pre-existing anticoagulation | 3-2 |  | 0,200 | 0,155 | 0,116 |  | 0,182 | 0,166 | 0,043 | |
| Pre-existing contraceptive medication | 3-2 |  | 0,009 | 0,013 | 0,040 |  | 0,010 | 0,007 | 0,028 | |
| Pre-existing number of outpatient treatments | 3-2 |  | 9,326 | 9,534 | 0,035 |  | 9,307 | 9,276 | 0,005 | |
| Pre-existing number of rehospitalizations | 3-2 |  | 1,613 | 1,279 | 0,158 |  | 1,366 | 1,175 | 0,091 | |
| Pre-existing immunosuppression incl. asplenia | 3-2 |  | 0,348 | 0,299 | 0,104 |  | 0,314 | 0,300 | 0,031 | |
| Pre-existing medical immunosuppression | 3-2 |  | 0,212 | 0,255 | 0,103 |  | 0,199 | 0,190 | 0,021 | |
| Pre-existing asplenia | 3-2 |  | 0,005 | 0,000 | 0,095 |  | 0,004 | 0,000 | 0,075 | |
| Prior organ transplantation | 3-2 |  | 0,012 | 0,015 | 0,020 |  | 0,011 | 0,015 | 0,027 | |
| Pre-existing infection/colonisation with multi-resistant pathogens | 3-2 |  | 0,044 | 0,026 | 0,099 |  | 0,035 | 0,023 | 0,069 | |
| Previously implanted foreign material | 3-2 |  | 0,285 | 0,200 | 0,198 |  | 0,259 | 0,211 | 0,113 | |
| Pre-existing mechanical ventilation | 3-2 |  | 0,111 | 0,102 | 0,030 |  | 0,090 | 0,085 | 0,017 | |
| Pre-existing long-term mechanical ventilation | 3-2 |  | 0,032 | 0,041 | 0,046 |  | 0,026 | 0,030 | 0,018 | |
| Pre-existing dialysis | 3-2 |  | 0,032 | 0,044 | 0,059 |  | 0,030 | 0,035 | 0,025 | |
| Pre-existing immobility | 3-2 |  | 0,252 | 0,193 | 0,141 |  | 0,221 | 0,199 | 0,051 | |
| Prior palliative treatment | 3-2 |  | 0,031 | 0,020 | 0,067 |  | 0,026 | 0,023 | 0,018 | |
| Any pre-existing pneumological diagnosis | 3-2 |  | 0,386 | 0,427 | 0,082 |  | 0,355 | 0,375 | 0,040 | |
| Pre-existing respiratory insufficiency, dyspnoea, respiratory distress syndrome | 3-2 |  | 0,298 | 0,329 | 0,069 |  | 0,263 | 0,278 | 0,032 | |
| Prior abnormal lung function results | 3-2 |  | 0,023 | 0,032 | 0,052 |  | 0,023 | 0,023 | 0,002 | |
| Pre-existing cardiovascular diagnoses | 3-2 |  | 0,799 | 0,792 | 0,017 |  | 0,789 | 0,793 | 0,012 | |
| Pre-existing renal diagnoses | 3-2 |  | 0,334 | 0,305 | 0,064 |  | 0,311 | 0,305 | 0,014 | |
| Pre-existing metabolic diagnoses | 3-2 |  | 0,425 | 0,421 | 0,008 |  | 0,423 | 0,426 | 0,005 | |
| Pre-existing anemia | 3-2 |  | 0,211 | 0,176 | 0,090 |  | 0,186 | 0,179 | 0,017 | |
| Pre-existing hepatic diagnoses | 3-2 |  | 0,121 | 0,116 | 0,015 |  | 0,117 | 0,109 | 0,023 | |
| Pre-existing gastroenterological diagnoses | 3-2 |  | 0,355 | 0,292 | 0,135 |  | 0,334 | 0,292 | 0,089 | |
| Any pre-existing ENT diagnosis | 3-2 |  | 0,315 | 0,253 | 0,139 |  | 0,303 | 0,290 | 0,029 | |
| Pre-existing dysphagia | 3-2 |  | 0,070 | 0,036 | 0,149 |  | 0,057 | 0,044 | 0,056 | |
| Pre-existing tracheostoma-related diagnoses | 3-2 |  | 0,027 | 0,004 | 0,183 |  | 0,020 | 0,006 | 0,115 | |
| Pre-existing taste and smelling disorders | 3-2 |  | 0,003 | 0,004 | 0,028 |  | 0,003 | 0,003 | 0,013 | |
| Pre-existing tinnitus | 3-2 |  | 0,029 | 0,028 | 0,008 |  | 0,032 | 0,038 | 0,033 | |
| Pre-existing dermatological diagnoses | 3-2 |  | 0,283 | 0,241 | 0,095 |  | 0,263 | 0,246 | 0,038 | |
| Pre-existing urogynecological diagnoses | 3-2 |  | 0,300 | 0,277 | 0,050 |  | 0,283 | 0,290 | 0,014 | |
| Pre-existing ophthalmological diagnoses | 3-2 |  | 0,168 | 0,170 | 0,006 |  | 0,174 | 0,169 | 0,014 | |
| Pre-existing musculoskeletal diagnoses | 3-2 |  | 0,377 | 0,303 | 0,156 |  | 0,335 | 0,312 | 0,048 | |
| Pre-existing neurological diagnoses | 3-2 |  | 0,145 | 0,120 | 0,072 |  | 0,132 | 0,126 | 0,018 | |
| Pre-existing Intensive Care Unit-Acquired Weakness | 3-2 |  | 0,121 | 0,118 | 0,010 |  | 0,113 | 0,113 | 0,001 | |
| Pre-existing cognitive diagnoses | 3-2 |  | 0,249 | 0,173 | 0,188 |  | 0,214 | 0,177 | 0,090 | |
| Pre-existing (mild) cognitive impairment | 3-2 |  | 0,094 | 0,065 | 0,107 |  | 0,079 | 0,071 | 0,031 | |
| Pre-existing dementia | 3-2 |  | 0,108 | 0,084 | 0,081 |  | 0,096 | 0,085 | 0,040 | |
| Pre-existing psychological diagnoses | 3-2 |  | 0,628 | 0,653 | 0,053 |  | 0,597 | 0,615 | 0,036 | |
| Other pre-existing psychological diagnoses | 3-2 |  | 0,231 | 0,229 | 0,005 |  | 0,225 | 0,216 | 0,022 | |
| Pre-existing depression | 3-2 |  | 0,293 | 0,305 | 0,026 |  | 0,285 | 0,286 | 0,003 | |
| Pre-existing anxiety | 3-2 |  | 0,098 | 0,126 | 0,089 |  | 0,095 | 0,098 | 0,008 | |
| Pre-existing trauma- and stressor-related disorders, PTSD | 3-2 |  | 0,086 | 0,091 | 0,019 |  | 0,082 | 0,086 | 0,017 | |
| Pre-existing sleep disorders | 3-2 |  | 0,183 | 0,187 | 0,012 |  | 0,178 | 0,179 | 0,002 | |
| Pre-existing substance use disorders | 3-2 |  | 0,296 | 0,313 | 0,037 |  | 0,251 | 0,252 | 0,001 | |
| Pre-existing impairment of the general condition | 3-2 |  | 0,632 | 0,611 | 0,043 |  | 0,634 | 0,605 | 0,058 | |
| Pre-existing impairment of nutrition | 3-2 |  | 0,110 | 0,070 | 0,143 |  | 0,089 | 0,061 | 0,098 | |
| Pre-existing fatigue | 3-2 |  | 0,085 | 0,070 | 0,056 |  | 0,076 | 0,060 | 0,059 | |
| Pre-existing (chronic) pain | 3-2 |  | 0,578 | 0,565 | 0,026 |  | 0,589 | 0,566 | 0,046 | |
| Pre-existing medical diagnoses | 3-2 |  | 0,949 | 0,955 | 0,030 |  | 0,948 | 0,949 | 0,004 | |
| Pre-existing venous thromboembolism | 3-2 |  | 0,037 | 0,019 | 0,109 |  | 0,034 | 0,020 | 0,084 | |
| Pre-existing hair loss | 3-2 |  | 0,007 | 0,009 | 0,023 |  | 0,006 | 0,006 | 0,003 | |
| Pre-existing disturbances of smell and taste | 3-2 |  | 0,003 | 0,004 | 0,028 |  | 0,003 | 0,003 | 0,013 | |
| Pre-existing post-exertional malaise | 3-2 |  | 0,098 | 0,084 | 0,049 |  | 0,089 | 0,074 | 0,052 | |
| Pre-existing cough | 3-2 |  | 0,050 | 0,060 | 0,044 |  | 0,052 | 0,059 | 0,027 | |
| Pre-existing brain fog | 3-2 |  | 0,094 | 0,065 | 0,107 |  | 0,079 | 0,071 | 0,031 | |
| Pre-existing polydipsia | 3-2 |  | 0,001 | 0,001 | 0,014 |  | 0,001 | 0,001 | 0,003 | |
| Pre-existing palpitations | 3-2 |  | 0,005 | 0,007 | 0,029 |  | 0,005 | 0,007 | 0,027 | |
| Pre-existing chest pain | 3-2 |  | 0,042 | 0,057 | 0,067 |  | 0,046 | 0,048 | 0,011 | |
| Pre-existing fatigue | 3-2 |  | 0,003 | 0,003 | 0,009 |  | 0,003 | 0,002 | 0,012 | |
| Pre-existing sexual dysfunction | 3-2 |  | 0,034 | 0,038 | 0,019 |  | 0,036 | 0,037 | 0,001 | |
| Pre-existing dizziness | 3-2 |  | 0,114 | 0,089 | 0,084 |  | 0,112 | 0,113 | 0,003 | |
| Pre-existing gastrointestinal disorders | 3-2 |  | 0,155 | 0,113 | 0,123 |  | 0,139 | 0,110 | 0,083 | |
| Pre-existing movement disorders | 3-2 |  | 0,034 | 0,032 | 0,013 |  | 0,033 | 0,031 | 0,007 | |
| Charlson: Cerebrovascular disease | 3-2 |  | 0,254 | 0,189 | 0,159 |  | 0,234 | 0,198 | 0,087 | |
| Charlson: Dementia | 3-2 |  | 0,111 | 0,089 | 0,075 |  | 0,099 | 0,091 | 0,026 | |
| Charlson: Peptic ulcer disease | 3-2 |  | 0,043 | 0,028 | 0,082 |  | 0,036 | 0,024 | 0,064 | |
| Charlson: Renal disease | 3-2 |  | 0,303 | 0,287 | 0,033 |  | 0,285 | 0,286 | 0,003 | |
| Elixhauser: Alcohol abuse | 3-2 |  | 0,130 | 0,109 | 0,065 |  | 0,106 | 0,094 | 0,038 | |
| Elixhauser: Cardiac arrhythmias | 3-2 |  | 0,327 | 0,293 | 0,072 |  | 0,299 | 0,302 | 0,005 | |
| Elixhauser: Chronic pulmonary disease | 3-2 |  | 0,382 | 0,512 | 0,265 |  | 0,363 | 0,371 | 0,017 | |
| Elixhauser: Congestive heart failure | 3-2 |  | 0,338 | 0,335 | 0,007 |  | 0,310 | 0,325 | 0,032 | |
| Elixhauser: Depression | 3-2 |  | 0,309 | 0,318 | 0,018 |  | 0,301 | 0,299 | 0,005 | |
| Elixhauser: Diabetes, complicated | 3-2 |  | 0,277 | 0,250 | 0,062 |  | 0,271 | 0,260 | 0,026 | |
| Elixhauser: Diabetes, uncomplicated | 3-2 |  | 0,396 | 0,380 | 0,032 |  | 0,395 | 0,385 | 0,020 | |
| Elixhauser: Fluid and electrolyte disorders | 3-2 |  | 0,301 | 0,235 | 0,149 |  | 0,255 | 0,230 | 0,057 | |
| Elixhauser: Hypertension, complicated | 3-2 |  | 0,207 | 0,215 | 0,019 |  | 0,200 | 0,208 | 0,021 | |
| Elixhauser: Hypothyroidism | 3-2 |  | 0,167 | 0,157 | 0,027 |  | 0,164 | 0,149 | 0,039 | |
| Elixhauser: Liver disease | 3-2 |  | 0,218 | 0,210 | 0,019 |  | 0,208 | 0,214 | 0,016 | |
| Elixhauser: Lymphoma | 3-2 |  | 0,020 | 0,020 | 0,001 |  | 0,018 | 0,019 | 0,004 | |
| Elixhauser: Obesity | 3-2 |  | 0,304 | 0,306 | 0,005 |  | 0,312 | 0,319 | 0,016 | |
| Elixhauser: Other neurological disorders | 3-2 |  | 0,161 | 0,118 | 0,125 |  | 0,137 | 0,109 | 0,080 | |
| Elixhauser: Paralysis | 3-2 |  | 0,113 | 0,074 | 0,134 |  | 0,098 | 0,076 | 0,076 | |
| Elixhauser: Peripheral vascular disorders | 3-2 |  | 0,299 | 0,251 | 0,106 |  | 0,271 | 0,260 | 0,024 | |
| Elixhauser: Psychoses | 3-2 |  | 0,053 | 0,067 | 0,058 |  | 0,049 | 0,058 | 0,036 | |
| Elixhauser: Pulmonary circulation disorders | 3-2 |  | 0,084 | 0,074 | 0,038 |  | 0,073 | 0,063 | 0,035 | |
| Elixhauser: Rheumatic arthritis/ collagen vascular diseases | 3-2 |  | 0,072 | 0,089 | 0,060 |  | 0,075 | 0,085 | 0,036 | |
| Elixhauser: Solid tumor without metastasis | 3-2 |  | 0,184 | 0,134 | 0,138 |  | 0,162 | 0,136 | 0,071 | |
| Elixhauser: Valvular disease | 3-2 |  | 0,190 | 0,174 | 0,041 |  | 0,177 | 0,169 | 0,020 |  |
| Elixhauser: Weight loss | 3-2 |  | 0,087 | 0,060 | 0,107 |  | 0,070 | 0,052 | 0,070 | |
| No pre-existing nursing care dependency | 3-2 |  | 0,606 | 0,672 | 0,138 |  | 0,647 | 0,687 | 0,084 | |
| Prior nursing care level 1 | 3-2 |  | 0,036 | 0,052 | 0,080 |  | 0,035 | 0,030 | 0,025 | |
| Prior nursing care level 2 | 3-2 |  | 0,151 | 0,107 | 0,129 |  | 0,134 | 0,114 | 0,062 | |
| Prior nursing care level 3 | 3-2 |  | 0,117 | 0,097 | 0,063 |  | 0,104 | 0,098 | 0,019 | |
| Prior nursing care level 4 | 3-2 |  | 0,058 | 0,042 | 0,072 |  | 0,052 | 0,043 | 0,038 | |
| Prior nursing care level 5 | 3-2 |  | 0,033 | 0,029 | 0,022 |  | 0,027 | 0,027 | 0,001 | |

Abbr.: ENT = Ear, Nose and Throat

# **e-Figure 2. Covariate balance checks based on absolute standardized mean differences in sub-sample of N = 11396 sepsis survivors with minimum survival time of 3 months after hospital discharge**


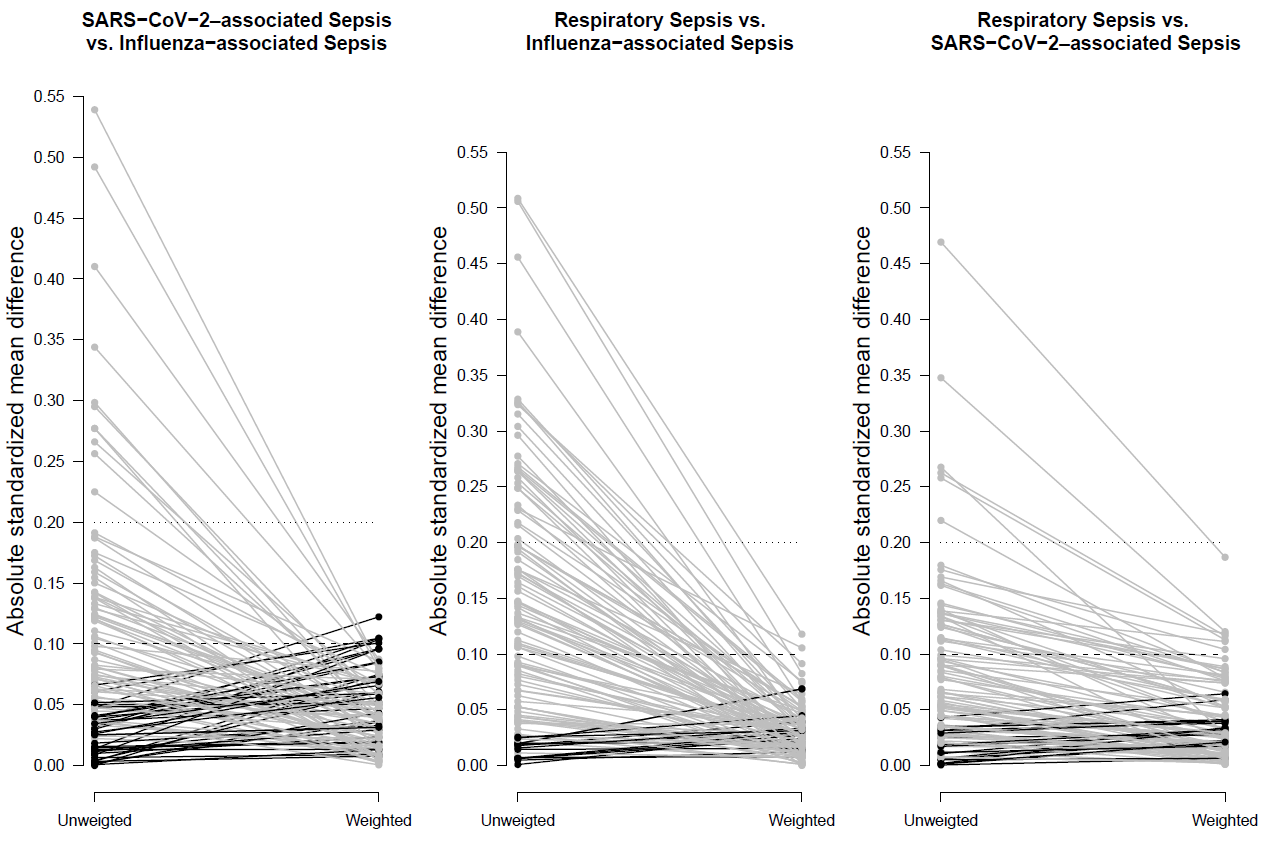


Pairwise unadjusted (e.g. unweighted) and adjusted (e.g. weighted IPTW) absolute standardized mean differences (ASMD) for the three groups of N = 11396 sepsis survivors with minimum survival time of 3 months

# **e-Table 2. Unadjusted (e.g. unweighted) and adjusted (e.g.weighted IPTW) means and absolute standardized mean differences (ASMD) between three groups of N = 11396 sepsis survivors with minimum survival time of 3 months (e.g., influenza-associated sepsis (group 1), SARS-CoV-2–associated sepsis (group 2), and respiratory Sepsis (group 3))**

|  |  |  | Unadjusted | | | |  | | Adjusted (IPTW) | | | |
| --- | --- | --- | --- | --- | --- | --- | --- | --- | --- | --- | --- | --- |
| Variable | Contrast group *j* - group *k* |  | M group *k* | M group *j* | ASMD |  | | M group *k* | | M group *j* | ASMD |  |
| Age | 2-1 |  | 64,698 | 63,179 | 0,106 |  | | 65,278 | | 65,597 | 0,022 |  |
| Sex | 2-1 |  | 0,438 | 0,377 | 0,124 |  | | 0,390 | | 0,372 | 0,038 |  |
| Infection of genitourinary system | 2-1 |  | 0,199 | 0,212 | 0,032 |  | | 0,257 | | 0,292 | 0,085 |  |
| Wound and soft tissue infection | 2-1 |  | 0,049 | 0,035 | 0,071 |  | | 0,064 | | 0,071 | 0,035 |  |
| Intraabdominal and retroperitoneal infection | 2-1 |  | 0,036 | 0,028 | 0,048 |  | | 0,074 | | 0,096 | 0,122 |  |
| Intrathoracic infection | 2-1 |  | 0,016 | 0,012 | 0,031 |  | | 0,014 | | 0,027 | 0,105 |  |
| Central nervous system infection | 2-1 |  | 0,008 | 0,005 | 0,040 |  | | 0,007 | | 0,012 | 0,059 |  |
| Bloodstream and vascular infection | 2-1 |  | 0,052 | 0,051 | 0,004 |  | | 0,071 | | 0,087 | 0,075 |  |
| Infection of bones, cartilage, joints | 2-1 |  | 0,016 | 0,004 | 0,124 |  | | 0,016 | | 0,012 | 0,042 |  |
| Infection of the gastrointestinal tract and diarrhea | 2-1 |  | 0,095 | 0,085 | 0,036 |  | | 0,105 | | 0,101 | 0,014 |  |
| Infections of the genital tract and sexually transmitted diseases | 2-1 |  | 0,011 | 0,007 | 0,045 |  | | 0,009 | | 0,013 | 0,042 |  |
| Device-related infection | 2-1 |  | 0,052 | 0,041 | 0,054 |  | | 0,069 | | 0,071 | 0,007 |  |
| Pre-existing inpatient nursing care | 2-1 |  | 0,047 | 0,040 | 0,037 |  | | 0,045 | | 0,060 | 0,073 |  |
| Previous vaccination against influenza | 2-1 |  | 0,295 | 0,311 | 0,033 |  | | 0,313 | | 0,320 | 0,016 |  |
| Pre-existing anticoagulation | 2-1 |  | 0,153 | 0,136 | 0,050 |  | | 0,160 | | 0,178 | 0,050 |  |
| Pre-existing contraceptive medication | 2-1 |  | 0,014 | 0,014 | 0,002 |  | | 0,008 | | 0,012 | 0,036 |  |
| Pre-existing number of outpatient treatments | 2-1 |  | 9,501 | 9,570 | 0,012 |  | | 9,233 | | 9,448 | 0,037 |  |
| Pre-existing number of rehospitalizations | 2-1 |  | 1,239 | 0,742 | 0,277 |  | | 1,132 | | 1,193 | 0,034 |  |
| Pre-existing immunosuppression incl. asplenia | 2-1 |  | 0,288 | 0,219 | 0,159 |  | | 0,285 | | 0,297 | 0,027 |  |
| Pre-existing medical immunosuppression | 2-1 |  | 0,262 | 0,144 | 0,298 |  | | 0,196 | | 0,182 | 0,036 |  |
| Pre-existing asplenia | 2-1 |  | 0,000 | 0,002 | 0,061 |  | | 0,000 | | 0,003 | 0,105 |  |
| Prior organ transplantation | 2-1 |  | 0,013 | 0,010 | 0,022 |  | | 0,014 | | 0,012 | 0,014 |  |
| Pre-existing infection/colonisation with multi-resistant pathogens | 2-1 |  | 0,027 | 0,014 | 0,087 |  | | 0,024 | | 0,029 | 0,034 |  |
| Previously implanted foreign material | 2-1 |  | 0,199 | 0,187 | 0,030 |  | | 0,210 | | 0,248 | 0,096 |  |
| Pre-existing mechanical ventilation | 2-1 |  | 0,103 | 0,030 | 0,295 |  | | 0,083 | | 0,071 | 0,049 |  |
| Pre-existing long-term mechanical ventilation | 2-1 |  | 0,041 | 0,011 | 0,187 |  | | 0,030 | | 0,024 | 0,037 |  |
| Pre-existing dialysis | 2-1 |  | 0,043 | 0,028 | 0,082 |  | | 0,036 | | 0,032 | 0,019 |  |
| Pre-existing immobility | 2-1 |  | 0,180 | 0,130 | 0,138 |  | | 0,185 | | 0,195 | 0,026 |  |
| Prior palliative treatment | 2-1 |  | 0,013 | 0,014 | 0,014 |  | | 0,011 | | 0,023 | 0,104 |  |
| Any pre-existing pneumological diagnosis | 2-1 |  | 0,425 | 0,264 | 0,344 |  | | 0,369 | | 0,332 | 0,079 |  |
| Pre-existing respiratory insufficiency, dyspnoea, respiratory distress syndrome | 2-1 |  | 0,327 | 0,155 | 0,410 |  | | 0,270 | | 0,233 | 0,087 |  |
| Prior abnormal lung function results | 2-1 |  | 0,032 | 0,020 | 0,075 |  | | 0,022 | | 0,018 | 0,029 |  |
| Pre-existing cardiovascular diagnoses | 2-1 |  | 0,776 | 0,736 | 0,093 |  | | 0,772 | | 0,779 | 0,017 |  |
| Pre-existing renal diagnoses | 2-1 |  | 0,288 | 0,234 | 0,122 |  | | 0,289 | | 0,305 | 0,037 |  |
| Pre-existing metabolic diagnoses | 2-1 |  | 0,414 | 0,423 | 0,018 |  | | 0,415 | | 0,432 | 0,033 |  |
| Pre-existing anemia | 2-1 |  | 0,166 | 0,124 | 0,119 |  | | 0,170 | | 0,177 | 0,020 |  |
| Pre-existing hepatic diagnoses | 2-1 |  | 0,115 | 0,119 | 0,010 |  | | 0,111 | | 0,118 | 0,023 |  |
| Pre-existing gastroenterological diagnoses | 2-1 |  | 0,286 | 0,304 | 0,039 |  | | 0,281 | | 0,325 | 0,096 |  |
| Any pre-existing ENT diagnosis | 2-1 |  | 0,242 | 0,301 | 0,133 |  | | 0,273 | | 0,303 | 0,068 |  |
| Pre-existing dysphagia | 2-1 |  | 0,033 | 0,027 | 0,037 |  | | 0,040 | | 0,048 | 0,047 |  |
| Pre-existing tracheostoma-related diagnoses | 2-1 |  | 0,005 | 0,005 | 0,000 |  | | 0,006 | | 0,011 | 0,072 |  |
| Pre-existing taste and smelling disorders | 2-1 |  | 0,005 | 0,003 | 0,025 |  | | 0,004 | | 0,006 | 0,031 |  |
| Pre-existing tinnitus | 2-1 |  | 0,027 | 0,055 | 0,143 |  | | 0,034 | | 0,040 | 0,026 |  |
| Pre-existing dermatological diagnoses | 2-1 |  | 0,237 | 0,215 | 0,053 |  | | 0,243 | | 0,242 | 0,003 |  |
| Pre-existing urogynecological diagnoses | 2-1 |  | 0,270 | 0,254 | 0,038 |  | | 0,277 | | 0,269 | 0,018 |  |
| Pre-existing ophthalmological diagnoses | 2-1 |  | 0,166 | 0,194 | 0,074 |  | | 0,161 | | 0,180 | 0,049 |  |
| Pre-existing musculoskeletal diagnoses | 2-1 |  | 0,288 | 0,217 | 0,163 |  | | 0,301 | | 0,299 | 0,005 |  |
| Pre-existing neurological diagnoses | 2-1 |  | 0,115 | 0,097 | 0,061 |  | | 0,117 | | 0,131 | 0,047 |  |
| Pre-existing Intensive Care Unit-Acquired Weakness | 2-1 |  | 0,115 | 0,087 | 0,093 |  | | 0,110 | | 0,108 | 0,007 |  |
| Pre-existing cognitive diagnoses | 2-1 |  | 0,156 | 0,122 | 0,098 |  | | 0,160 | | 0,186 | 0,077 |  |
| Pre-existing (mild) cognitive impairment | 2-1 |  | 0,057 | 0,041 | 0,072 |  | | 0,060 | | 0,062 | 0,008 |  |
| Pre-existing dementia | 2-1 |  | 0,074 | 0,062 | 0,048 |  | | 0,075 | | 0,089 | 0,052 |  |
| Pre-existing psychological diagnoses | 2-1 |  | 0,645 | 0,514 | 0,266 |  | | 0,599 | | 0,570 | 0,058 |  |
| Other pre-existing psychological diagnoses | 2-1 |  | 0,227 | 0,228 | 0,001 |  | | 0,212 | | 0,230 | 0,043 |  |
| Pre-existing depression | 2-1 |  | 0,303 | 0,272 | 0,069 |  | | 0,278 | | 0,279 | 0,002 |  |
| Pre-existing anxiety | 2-1 |  | 0,128 | 0,096 | 0,101 |  | | 0,095 | | 0,099 | 0,012 |  |
| Pre-existing trauma- and stressor-related disorders, PTSD | 2-1 |  | 0,095 | 0,077 | 0,063 |  | | 0,087 | | 0,072 | 0,051 |  |
| Pre-existing sleep disorders | 2-1 |  | 0,179 | 0,180 | 0,004 |  | | 0,165 | | 0,188 | 0,061 |  |
| Pre-existing substance use disorders | 2-1 |  | 0,319 | 0,106 | 0,539 |  | | 0,256 | | 0,223 | 0,083 |  |
| Pre-existing impairment of the general condition | 2-1 |  | 0,605 | 0,667 | 0,129 |  | | 0,598 | | 0,630 | 0,067 |  |
| Pre-existing impairment of nutrition | 2-1 |  | 0,065 | 0,035 | 0,138 |  | | 0,056 | | 0,070 | 0,068 |  |
| Pre-existing fatigue | 2-1 |  | 0,071 | 0,057 | 0,057 |  | | 0,061 | | 0,065 | 0,016 |  |
| Pre-existing (chronic) pain | 2-1 |  | 0,559 | 0,651 | 0,188 |  | | 0,558 | | 0,600 | 0,086 |  |
| Pre-existing medical diagnoses | 2-1 |  | 0,951 | 0,948 | 0,016 |  | | 0,944 | | 0,948 | 0,019 |  |
| Pre-existing venous thromboembolism | 2-1 |  | 0,017 | 0,027 | 0,066 |  | | 0,020 | | 0,035 | 0,101 |  |
| Pre-existing hair loss | 2-1 |  | 0,009 | 0,011 | 0,013 |  | | 0,007 | | 0,008 | 0,013 |  |
| Pre-existing disturbances of smell and taste | 2-1 |  | 0,005 | 0,003 | 0,025 |  | | 0,004 | | 0,006 | 0,031 |  |
| Pre-existing post-exertional malaise | 2-1 |  | 0,085 | 0,076 | 0,036 |  | | 0,075 | | 0,077 | 0,010 |  |
| Pre-existing cough | 2-1 |  | 0,058 | 0,059 | 0,003 |  | | 0,058 | | 0,060 | 0,008 |  |
| Pre-existing brain fog | 2-1 |  | 0,057 | 0,041 | 0,072 |  | | 0,060 | | 0,062 | 0,008 |  |
| Pre-existing polydipsia | 2-1 |  | 0,002 | 0,001 | 0,015 |  | | 0,001 | | 0,001 | 0,019 |  |
| Pre-existing palpitations | 2-1 |  | 0,006 | 0,007 | 0,007 |  | | 0,007 | | 0,006 | 0,009 |  |
| Pre-existing chest pain | 2-1 |  | 0,052 | 0,063 | 0,048 |  | | 0,046 | | 0,053 | 0,030 |  |
| Pre-existing fatigue | 2-1 |  | 0,002 | 0,004 | 0,041 |  | | 0,001 | | 0,005 | 0,073 |  |
| Pre-existing sexual dysfunction | 2-1 |  | 0,039 | 0,047 | 0,038 |  | | 0,039 | | 0,039 | 0,001 |  |
| Pre-existing dizziness | 2-1 |  | 0,084 | 0,117 | 0,111 |  | | 0,099 | | 0,113 | 0,049 |  |
| Pre-existing gastrointestinal disorders | 2-1 |  | 0,100 | 0,097 | 0,009 |  | | 0,093 | | 0,122 | 0,096 |  |
| Pre-existing movement disorders | 2-1 |  | 0,025 | 0,027 | 0,012 |  | | 0,027 | | 0,031 | 0,029 |  |
| Charlson: Cerebrovascular disease | 2-1 |  | 0,185 | 0,170 | 0,040 |  | | 0,188 | | 0,220 | 0,085 |  |
| Charlson: Dementia | 2-1 |  | 0,077 | 0,063 | 0,056 |  | | 0,079 | | 0,089 | 0,040 |  |
| Charlson: Peptic ulcer disease | 2-1 |  | 0,030 | 0,018 | 0,081 |  | | 0,027 | | 0,029 | 0,016 |  |
| Charlson: Renal disease | 2-1 |  | 0,272 | 0,226 | 0,105 |  | | 0,273 | | 0,289 | 0,038 |  |
| Elixhauser: Alcohol abuse | 2-1 |  | 0,112 | 0,040 | 0,277 |  | | 0,097 | | 0,094 | 0,011 |  |
| Elixhauser: Cardiac arrhythmias | 2-1 |  | 0,284 | 0,219 | 0,150 |  | | 0,295 | | 0,273 | 0,051 |  |
| Elixhauser: Chronic pulmonary disease | 2-1 |  | 0,513 | 0,280 | 0,492 |  | | 0,376 | | 0,338 | 0,080 |  |
| Elixhauser: Congestive heart failure | 2-1 |  | 0,322 | 0,210 | 0,256 |  | | 0,310 | | 0,292 | 0,042 |  |
| Elixhauser: Depression | 2-1 |  | 0,318 | 0,287 | 0,066 |  | | 0,292 | | 0,293 | 0,003 |  |
| Elixhauser: Diabetes, complicated | 2-1 |  | 0,246 | 0,247 | 0,001 |  | | 0,260 | | 0,266 | 0,013 |  |
| Elixhauser: Diabetes, uncomplicated | 2-1 |  | 0,373 | 0,398 | 0,052 |  | | 0,377 | | 0,406 | 0,059 |  |
| Elixhauser: Fluid and electrolyte disorders | 2-1 |  | 0,213 | 0,129 | 0,225 |  | | 0,201 | | 0,224 | 0,059 |  |
| Elixhauser: Hypertension, complicated | 2-1 |  | 0,210 | 0,181 | 0,073 |  | | 0,207 | | 0,198 | 0,024 |  |
| Elixhauser: Hypothyroidism | 2-1 |  | 0,156 | 0,169 | 0,034 |  | | 0,148 | | 0,172 | 0,066 |  |
| Elixhauser: Liver disease | 2-1 |  | 0,209 | 0,178 | 0,078 |  | | 0,211 | | 0,209 | 0,003 |  |
| Elixhauser: Lymphoma | 2-1 |  | 0,019 | 0,011 | 0,063 |  | | 0,017 | | 0,014 | 0,024 |  |
| Elixhauser: Obesity | 2-1 |  | 0,302 | 0,358 | 0,120 |  | | 0,311 | | 0,325 | 0,030 |  |
| Elixhauser: Other neurological disorders | 2-1 |  | 0,115 | 0,071 | 0,155 |  | | 0,111 | | 0,112 | 0,004 |  |
| Elixhauser: Paralysis | 2-1 |  | 0,073 | 0,053 | 0,083 |  | | 0,075 | | 0,089 | 0,059 |  |
| Elixhauser: Peripheral vascular disorders | 2-1 |  | 0,242 | 0,185 | 0,138 |  | | 0,254 | | 0,252 | 0,004 |  |
| Elixhauser: Psychoses | 2-1 |  | 0,070 | 0,031 | 0,175 |  | | 0,059 | | 0,043 | 0,075 |  |
| Elixhauser: Pulmonary circulation disorders | 2-1 |  | 0,077 | 0,038 | 0,169 |  | | 0,067 | | 0,058 | 0,039 |  |
| Elixhauser: Rheumatic arthritis/ collagen vascular diseases | 2-1 |  | 0,084 | 0,077 | 0,024 |  | | 0,076 | | 0,082 | 0,023 |  |
| Elixhauser: Solid tumor without metastasis | 2-1 |  | 0,122 | 0,097 | 0,079 |  | | 0,117 | | 0,140 | 0,071 |  |
| Elixhauser: Valvular disease | 2-1 |  | 0,177 | 0,133 | 0,121 |  | | 0,175 | | 0,172 | 0,009 |  |
| Elixhauser: Weight loss | 2-1 |  | 0,058 | 0,024 | 0,173 |  | | 0,051 | | 0,056 | 0,027 |  |
| No pre-existing nursing care dependency | 2-1 |  | 0,689 | 0,773 | 0,191 |  | | 0,708 | | 0,679 | 0,067 |  |
| Prior nursing care level 1 | 2-1 |  | 0,055 | 0,027 | 0,142 |  | | 0,033 | | 0,034 | 0,007 |  |
| Prior nursing care level 2 | 2-1 |  | 0,098 | 0,090 | 0,026 |  | | 0,100 | | 0,120 | 0,069 |  |
| Prior nursing care level 3 | 2-1 |  | 0,093 | 0,067 | 0,095 |  | | 0,097 | | 0,100 | 0,014 |  |
| Prior nursing care level 4 | 2-1 |  | 0,036 | 0,031 | 0,028 |  | | 0,036 | | 0,046 | 0,056 |  |
| Prior nursing care level 5 | 2-1 |  | 0,028 | 0,011 | 0,130 |  | | 0,026 | | 0,021 | 0,041 |  |
| Age | 3-1 |  | 65,634 | 63,179 | 0,172 |  | | 65,105 | | 65,597 | 0,034 |  |
| Sex | 3-1 |  | 0,357 | 0,377 | 0,041 |  | | 0,365 | | 0,372 | 0,015 |  |
| Infection of genitourinary system | 3-1 |  | 0,352 | 0,212 | 0,315 |  | | 0,310 | | 0,292 | 0,042 |  |
| Wound and soft tissue infection | 3-1 |  | 0,120 | 0,035 | 0,324 |  | | 0,095 | | 0,071 | 0,091 |  |
| Intraabdominal and retroperitoneal infection | 3-1 |  | 0,178 | 0,028 | 0,509 |  | | 0,131 | | 0,096 | 0,118 |  |
| Intrathoracic infection | 3-1 |  | 0,045 | 0,012 | 0,197 |  | | 0,035 | | 0,027 | 0,048 |  |
| Central nervous system infection | 3-1 |  | 0,021 | 0,005 | 0,146 |  | | 0,016 | | 0,012 | 0,038 |  |
| Bloodstream and vascular infection | 3-1 |  | 0,126 | 0,051 | 0,266 |  | | 0,104 | | 0,087 | 0,058 |  |
| Infection of bones, cartilage, joints | 3-1 |  | 0,035 | 0,004 | 0,230 |  | | 0,026 | | 0,012 | 0,106 |  |
| Infection of the gastrointestinal tract and diarrhea | 3-1 |  | 0,111 | 0,085 | 0,090 |  | | 0,103 | | 0,101 | 0,008 |  |
| Infections of the genital tract and sexually transmitted diseases | 3-1 |  | 0,024 | 0,007 | 0,137 |  | | 0,018 | | 0,013 | 0,047 |  |
| Device-related infection | 3-1 |  | 0,112 | 0,041 | 0,271 |  | | 0,090 | | 0,071 | 0,076 |  |
| Pre-existing inpatient nursing care | 3-1 |  | 0,059 | 0,040 | 0,089 |  | | 0,051 | | 0,060 | 0,039 |  |
| Previous vaccination against influenza | 3-1 |  | 0,324 | 0,311 | 0,028 |  | | 0,319 | | 0,320 | 0,001 |  |
| Pre-existing anticoagulation | 3-1 |  | 0,189 | 0,136 | 0,146 |  | | 0,173 | | 0,178 | 0,015 |  |
| Pre-existing contraceptive medication | 3-1 |  | 0,010 | 0,014 | 0,040 |  | | 0,011 | | 0,012 | 0,013 |  |
| Pre-existing number of outpatient treatments | 3-1 |  | 9,236 | 9,570 | 0,058 |  | | 9,226 | | 9,448 | 0,038 |  |
| Pre-existing number of rehospitalizations | 3-1 |  | 1,537 | 0,742 | 0,456 |  | | 1,292 | | 1,193 | 0,057 |  |
| Pre-existing immunosuppression incl. asplenia | 3-1 |  | 0,329 | 0,219 | 0,249 |  | | 0,296 | | 0,297 | 0,002 |  |
| Pre-existing medical immunosuppression | 3-1 |  | 0,206 | 0,144 | 0,164 |  | | 0,194 | | 0,182 | 0,032 |  |
| Pre-existing asplenia | 3-1 |  | 0,005 | 0,002 | 0,052 |  | | 0,004 | | 0,003 | 0,010 |  |
| Prior organ transplantation | 3-1 |  | 0,012 | 0,010 | 0,021 |  | | 0,012 | | 0,012 | 0,007 |  |
| Pre-existing infection/colonisation with multi-resistant pathogens | 3-1 |  | 0,041 | 0,014 | 0,161 |  | | 0,032 | | 0,029 | 0,018 |  |
| Previously implanted foreign material | 3-1 |  | 0,267 | 0,187 | 0,192 |  | | 0,242 | | 0,248 | 0,014 |  |
| Pre-existing mechanical ventilation | 3-1 |  | 0,113 | 0,030 | 0,325 |  | | 0,090 | | 0,071 | 0,074 |  |
| Pre-existing long-term mechanical ventilation | 3-1 |  | 0,032 | 0,011 | 0,142 |  | | 0,026 | | 0,024 | 0,009 |  |
| Pre-existing dialysis | 3-1 |  | 0,031 | 0,028 | 0,020 |  | | 0,029 | | 0,032 | 0,021 |  |
| Pre-existing immobility | 3-1 |  | 0,231 | 0,130 | 0,264 |  | | 0,201 | | 0,195 | 0,017 |  |
| Prior palliative treatment | 3-1 |  | 0,025 | 0,014 | 0,077 |  | | 0,021 | | 0,023 | 0,014 |  |
| Any pre-existing pneumological diagnosis | 3-1 |  | 0,379 | 0,264 | 0,249 |  | | 0,347 | | 0,332 | 0,033 |  |
| Pre-existing respiratory insufficiency, dyspnoea, respiratory distress syndrome | 3-1 |  | 0,290 | 0,155 | 0,329 |  | | 0,254 | | 0,233 | 0,051 |  |
| Prior abnormal lung function results | 3-1 |  | 0,022 | 0,020 | 0,018 |  | | 0,022 | | 0,018 | 0,029 |  |
| Pre-existing cardiovascular diagnoses | 3-1 |  | 0,787 | 0,736 | 0,120 |  | | 0,775 | | 0,779 | 0,010 |  |
| Pre-existing renal diagnoses | 3-1 |  | 0,316 | 0,234 | 0,185 |  | | 0,293 | | 0,305 | 0,026 |  |
| Pre-existing metabolic diagnoses | 3-1 |  | 0,414 | 0,423 | 0,018 |  | | 0,412 | | 0,432 | 0,039 |  |
| Pre-existing anemia | 3-1 |  | 0,196 | 0,124 | 0,198 |  | | 0,172 | | 0,177 | 0,013 |  |
| Pre-existing hepatic diagnoses | 3-1 |  | 0,121 | 0,119 | 0,007 |  | | 0,116 | | 0,118 | 0,008 |  |
| Pre-existing gastroenterological diagnoses | 3-1 |  | 0,347 | 0,304 | 0,091 |  | | 0,325 | | 0,325 | 0,001 |  |
| Any pre-existing ENT diagnosis | 3-1 |  | 0,301 | 0,301 | 0,001 |  | | 0,290 | | 0,303 | 0,028 |  |
| Pre-existing dysphagia | 3-1 |  | 0,062 | 0,027 | 0,173 |  | | 0,051 | | 0,048 | 0,014 |  |
| Pre-existing tracheostoma-related diagnoses | 3-1 |  | 0,026 | 0,005 | 0,176 |  | | 0,019 | | 0,011 | 0,064 |  |
| Pre-existing taste and smelling disorders | 3-1 |  | 0,002 | 0,003 | 0,020 |  | | 0,002 | | 0,006 | 0,069 |  |
| Pre-existing tinnitus | 3-1 |  | 0,029 | 0,055 | 0,131 |  | | 0,033 | | 0,040 | 0,035 |  |
| Pre-existing dermatological diagnoses | 3-1 |  | 0,271 | 0,215 | 0,132 |  | | 0,251 | | 0,242 | 0,021 |  |
| Pre-existing urogynecological diagnoses | 3-1 |  | 0,281 | 0,254 | 0,063 |  | | 0,265 | | 0,269 | 0,010 |  |
| Pre-existing ophthalmological diagnoses | 3-1 |  | 0,164 | 0,194 | 0,080 |  | | 0,170 | | 0,180 | 0,028 |  |
| Pre-existing musculoskeletal diagnoses | 3-1 |  | 0,353 | 0,217 | 0,304 |  | | 0,311 | | 0,299 | 0,028 |  |
| Pre-existing neurological diagnoses | 3-1 |  | 0,137 | 0,097 | 0,127 |  | | 0,125 | | 0,131 | 0,019 |  |
| Pre-existing Intensive Care Unit-Acquired Weakness | 3-1 |  | 0,119 | 0,087 | 0,106 |  | | 0,111 | | 0,108 | 0,009 |  |
| Pre-existing cognitive diagnoses | 3-1 |  | 0,227 | 0,122 | 0,277 |  | | 0,192 | | 0,186 | 0,016 |  |
| Pre-existing (mild) cognitive impairment | 3-1 |  | 0,090 | 0,041 | 0,197 |  | | 0,075 | | 0,062 | 0,053 |  |
| Pre-existing dementia | 3-1 |  | 0,090 | 0,062 | 0,106 |  | | 0,080 | | 0,089 | 0,033 |  |
| Pre-existing psychological diagnoses | 3-1 |  | 0,627 | 0,514 | 0,229 |  | | 0,594 | | 0,570 | 0,049 |  |
| Other pre-existing psychological diagnoses | 3-1 |  | 0,230 | 0,228 | 0,005 |  | | 0,225 | | 0,230 | 0,012 |  |
| Pre-existing depression | 3-1 |  | 0,290 | 0,272 | 0,040 |  | | 0,282 | | 0,279 | 0,007 |  |
| Pre-existing anxiety | 3-1 |  | 0,099 | 0,096 | 0,011 |  | | 0,096 | | 0,099 | 0,008 |  |
| Pre-existing trauma- and stressor-related disorders, PTSD | 3-1 |  | 0,088 | 0,077 | 0,038 |  | | 0,082 | | 0,072 | 0,037 |  |
| Pre-existing sleep disorders | 3-1 |  | 0,183 | 0,180 | 0,006 |  | | 0,177 | | 0,188 | 0,027 |  |
| Pre-existing substance use disorders | 3-1 |  | 0,304 | 0,106 | 0,506 |  | | 0,256 | | 0,223 | 0,082 |  |
| Pre-existing impairment of the general condition | 3-1 |  | 0,626 | 0,667 | 0,085 |  | | 0,629 | | 0,630 | 0,002 |  |
| Pre-existing impairment of nutrition | 3-1 |  | 0,101 | 0,035 | 0,264 |  | | 0,080 | | 0,070 | 0,039 |  |
| Pre-existing fatigue | 3-1 |  | 0,080 | 0,057 | 0,091 |  | | 0,072 | | 0,065 | 0,027 |  |
| Pre-existing (chronic) pain | 3-1 |  | 0,575 | 0,651 | 0,156 |  | | 0,588 | | 0,600 | 0,026 |  |
| Pre-existing medical diagnoses | 3-1 |  | 0,944 | 0,948 | 0,015 |  | | 0,944 | | 0,948 | 0,015 |  |
| Pre-existing venous thromboembolism | 3-1 |  | 0,036 | 0,027 | 0,049 |  | | 0,033 | | 0,035 | 0,010 |  |
| Pre-existing hair loss | 3-1 |  | 0,007 | 0,011 | 0,045 |  | | 0,006 | | 0,008 | 0,019 |  |
| Pre-exsiting disturbances of smell and taste | 3-1 |  | 0,002 | 0,003 | 0,020 |  | | 0,002 | | 0,006 | 0,069 |  |
| Pre-existing post-exertional malaise | 3-1 |  | 0,094 | 0,076 | 0,067 |  | | 0,086 | | 0,077 | 0,029 |  |
| Pre-existing cough | 3-1 |  | 0,051 | 0,059 | 0,038 |  | | 0,054 | | 0,060 | 0,028 |  |
| Pre-existing brain fog | 3-1 |  | 0,090 | 0,041 | 0,197 |  | | 0,075 | | 0,062 | 0,053 |  |
| Pre-existing polydipsia | 3-1 |  | 0,001 | 0,001 | 0,006 |  | | 0,001 | | 0,001 | 0,024 |  |
| Pre-existing palpitations | 3-1 |  | 0,005 | 0,007 | 0,024 |  | | 0,004 | | 0,006 | 0,018 |  |
| Pre-existing chest pain | 3-1 |  | 0,042 | 0,063 | 0,093 |  | | 0,047 | | 0,053 | 0,027 |  |
| Pre-existing fatigue | 3-1 |  | 0,003 | 0,004 | 0,007 |  | | 0,003 | | 0,005 | 0,030 |  |
| Pre-existing sexual dysfunction | 3-1 |  | 0,033 | 0,047 | 0,073 |  | | 0,035 | | 0,039 | 0,018 |  |
| Pre-existing dizziness | 3-1 |  | 0,108 | 0,117 | 0,027 |  | | 0,107 | | 0,113 | 0,019 |  |
| Pre-existing gastrointestinal disorders | 3-1 |  | 0,145 | 0,097 | 0,147 |  | | 0,130 | | 0,122 | 0,024 |  |
| Pre-existing movement disorders | 3-1 |  | 0,033 | 0,027 | 0,033 |  | | 0,031 | | 0,031 | 0,001 |  |
| Charlson: Cerebrovascular disease | 3-1 |  | 0,240 | 0,170 | 0,176 |  | | 0,220 | | 0,220 | 0,000 |  |
| Charlson: Dementia | 3-1 |  | 0,093 | 0,063 | 0,110 |  | | 0,082 | | 0,089 | 0,030 |  |
| Charlson: Peptic ulcer disease | 3-1 |  | 0,042 | 0,018 | 0,144 |  | | 0,035 | | 0,029 | 0,035 |  |
| Charlson: Renal disease | 3-1 |  | 0,284 | 0,226 | 0,133 |  | | 0,268 | | 0,289 | 0,050 |  |
| Elixhauser: Alcohol abuse | 3-1 |  | 0,129 | 0,040 | 0,326 |  | | 0,104 | | 0,094 | 0,034 |  |
| Elixhauser: Cardiac arrhythmias | 3-1 |  | 0,309 | 0,219 | 0,204 |  | | 0,282 | | 0,273 | 0,020 |  |
| Elixhauser: Chronic pulmonary disease | 3-1 |  | 0,382 | 0,280 | 0,218 |  | | 0,362 | | 0,338 | 0,052 |  |
| Elixhauser: Congestive heart failure | 3-1 |  | 0,323 | 0,210 | 0,258 |  | | 0,294 | | 0,292 | 0,005 |  |
| Elixhauser: Depression | 3-1 |  | 0,307 | 0,287 | 0,044 |  | | 0,299 | | 0,293 | 0,012 |  |
| Elixhauser: Diabetes, complicated | 3-1 |  | 0,270 | 0,247 | 0,053 |  | | 0,264 | | 0,266 | 0,003 |  |
| Elixhauser: Diabetes, uncomplicated | 3-1 |  | 0,386 | 0,398 | 0,025 |  | | 0,384 | | 0,406 | 0,045 |  |
| Elixhauser: Fluid and electrolyte disorders | 3-1 |  | 0,284 | 0,129 | 0,389 |  | | 0,238 | | 0,224 | 0,035 |  |
| Elixhauser: Hypertension, complicated | 3-1 |  | 0,198 | 0,181 | 0,044 |  | | 0,191 | | 0,198 | 0,016 |  |
| Elixhauser: Hypothyroidism | 3-1 |  | 0,163 | 0,169 | 0,016 |  | | 0,160 | | 0,172 | 0,033 |  |
| Elixhauser: Liver disease | 3-1 |  | 0,217 | 0,178 | 0,098 |  | | 0,206 | | 0,209 | 0,008 |  |
| Elixhauser: Lymphoma | 3-1 |  | 0,020 | 0,011 | 0,068 |  | | 0,018 | | 0,014 | 0,031 |  |
| Elixhauser: Obesity | 3-1 |  | 0,305 | 0,358 | 0,112 |  | | 0,312 | | 0,325 | 0,028 |  |
| Elixhauser: Other neurological disorders | 3-1 |  | 0,154 | 0,071 | 0,268 |  | | 0,130 | | 0,112 | 0,059 |  |
| Elixhauser: Paralysis | 3-1 |  | 0,105 | 0,053 | 0,194 |  | | 0,090 | | 0,089 | 0,003 |  |
| Elixhauser: Peripheral vascular disorders | 3-1 |  | 0,284 | 0,185 | 0,233 |  | | 0,256 | | 0,252 | 0,010 |  |
| Elixhauser: Psychoses | 3-1 |  | 0,053 | 0,031 | 0,108 |  | | 0,049 | | 0,043 | 0,028 |  |
| Elixhauser: Pulmonary circulation disorders | 3-1 |  | 0,079 | 0,038 | 0,176 |  | | 0,068 | | 0,058 | 0,042 |  |
| Elixhauser: Rheumatic arthritis/ collagen vascular diseases | 3-1 |  | 0,070 | 0,077 | 0,026 |  | | 0,074 | | 0,082 | 0,031 |  |
| Elixhauser: Solid tumor without metastasis | 3-1 |  | 0,170 | 0,097 | 0,216 |  | | 0,148 | | 0,140 | 0,025 |  |
| Elixhauser: Valvular disease | 3-1 |  | 0,181 | 0,133 | 0,133 |  | | 0,168 | | 0,172 | 0,010 |  |
| Elixhauser: Weight loss | 3-1 |  | 0,080 | 0,024 | 0,253 |  | | 0,064 | | 0,056 | 0,035 |  |
| No pre-existing nursing care dependency | 3-1 |  | 0,640 | 0,773 | 0,296 |  | | 0,680 | | 0,679 | 0,004 |  |
| Prior nursing care level 1 | 3-1 |  | 0,036 | 0,027 | 0,052 |  | | 0,035 | | 0,034 | 0,008 |  |
| Prior nursing care level 2 | 3-1 |  | 0,145 | 0,090 | 0,170 |  | | 0,129 | | 0,120 | 0,025 |  |
| Prior nursing care level 3 | 3-1 |  | 0,104 | 0,067 | 0,129 |  | | 0,091 | | 0,100 | 0,032 |  |
| Prior nursing care level 4 | 3-1 |  | 0,047 | 0,031 | 0,083 |  | | 0,042 | | 0,046 | 0,022 |  |
| Prior nursing care level 5 | 3-1 |  | 0,028 | 0,011 | 0,128 |  | | 0,023 | | 0,021 | 0,016 |  |
| Age | 3-2 |  | 65,634 | 64,698 | 0,066 |  | | 65,105 | | 65,278 | 0,012 |  |
| Sex | 3-2 |  | 0,357 | 0,438 | 0,166 |  | | 0,365 | | 0,390 | 0,053 |  |
| Infection of genitourinary system | 3-2 |  | 0,352 | 0,199 | 0,348 |  | | 0,310 | | 0,257 | 0,120 |  |
| Wound and soft tissue infection | 3-2 |  | 0,120 | 0,049 | 0,258 |  | | 0,095 | | 0,064 | 0,113 |  |
| Intraabdominal and retroperitoneal infection | 3-2 |  | 0,178 | 0,036 | 0,469 |  | | 0,131 | | 0,074 | 0,187 |  |
| Intrathoracic infection | 3-2 |  | 0,045 | 0,016 | 0,169 |  | | 0,035 | | 0,014 | 0,118 |  |
| Central nervous system infection | 3-2 |  | 0,021 | 0,008 | 0,112 |  | | 0,016 | | 0,007 | 0,075 |  |
| Bloodstream and vascular infection | 3-2 |  | 0,126 | 0,052 | 0,262 |  | | 0,104 | | 0,071 | 0,116 |  |
| Infection of bones, cartilage, joints | 3-2 |  | 0,035 | 0,016 | 0,124 |  | | 0,026 | | 0,016 | 0,066 |  |
| Infection of the gastrointestinal tract and diarrhea | 3-2 |  | 0,111 | 0,095 | 0,054 |  | | 0,103 | | 0,105 | 0,005 |  |
| Infections of the genital tract and sexually transmitted diseases | 3-2 |  | 0,024 | 0,011 | 0,096 |  | | 0,018 | | 0,009 | 0,074 |  |
| Device-related infection | 3-2 |  | 0,112 | 0,052 | 0,220 |  | | 0,090 | | 0,069 | 0,078 |  |
| Pre-existing inpatient nursing care | 3-2 |  | 0,059 | 0,047 | 0,052 |  | | 0,051 | | 0,045 | 0,029 |  |
| Previous vaccination against influenza | 3-2 |  | 0,324 | 0,295 | 0,062 |  | | 0,319 | | 0,313 | 0,014 |  |
| Pre-existing anticoagulation | 3-2 |  | 0,189 | 0,153 | 0,096 |  | | 0,173 | | 0,160 | 0,032 |  |
| Pre-existing contraceptive medication | 3-2 |  | 0,010 | 0,014 | 0,042 |  | | 0,011 | | 0,008 | 0,026 |  |
| Pre-existing number of outpatient treatments | 3-2 |  | 9,236 | 9,501 | 0,045 |  | | 9,226 | | 9,233 | 0,001 |  |
| Pre-existing number of rehospitalizations | 3-2 |  | 1,537 | 1,239 | 0,146 |  | | 1,292 | | 1,132 | 0,078 |  |
| Pre-existing immunosuppression incl. asplenia | 3-2 |  | 0,329 | 0,288 | 0,089 |  | | 0,296 | | 0,285 | 0,023 |  |
| Pre-existing medical immunosuppression | 3-2 |  | 0,206 | 0,262 | 0,134 |  | | 0,194 | | 0,196 | 0,005 |  |
| Pre-existing asplenia | 3-2 |  | 0,005 | 0,000 | 0,099 |  | | 0,004 | | 0,000 | 0,076 |  |
| Prior organ transplantation | 3-2 |  | 0,012 | 0,013 | 0,001 |  | | 0,012 | | 0,014 | 0,020 |  |
| Pre-existing infection/colonisation with multi-resistant pathogens | 3-2 |  | 0,041 | 0,027 | 0,077 |  | | 0,032 | | 0,024 | 0,043 |  |
| Previously implanted foreign material | 3-2 |  | 0,267 | 0,199 | 0,162 |  | | 0,242 | | 0,210 | 0,076 |  |
| Pre-existing mechanical ventilation | 3-2 |  | 0,113 | 0,103 | 0,032 |  | | 0,090 | | 0,083 | 0,022 |  |
| Pre-existing long-term mechanical ventilation | 3-2 |  | 0,032 | 0,041 | 0,049 |  | | 0,026 | | 0,030 | 0,024 |  |
| Pre-existing dialysis | 3-2 |  | 0,031 | 0,043 | 0,062 |  | | 0,029 | | 0,036 | 0,038 |  |
| Pre-existing immobility | 3-2 |  | 0,231 | 0,180 | 0,125 |  | | 0,201 | | 0,185 | 0,039 |  |
| Prior palliative treatment | 3-2 |  | 0,025 | 0,013 | 0,090 |  | | 0,021 | | 0,011 | 0,074 |  |
| Any pre-existing pneumological diagnosis | 3-2 |  | 0,379 | 0,425 | 0,094 |  | | 0,347 | | 0,369 | 0,045 |  |
| Pre-existing respiratory insufficiency, dyspnoea, respiratory distress syndrome | 3-2 |  | 0,290 | 0,327 | 0,080 |  | | 0,254 | | 0,270 | 0,034 |  |
| Prior abnormal lung function results | 3-2 |  | 0,022 | 0,032 | 0,057 |  | | 0,022 | | 0,022 | 0,003 |  |
| Pre-existing cardiovascular diagnoses | 3-2 |  | 0,787 | 0,776 | 0,027 |  | | 0,775 | | 0,772 | 0,007 |  |
| Pre-existing renal diagnoses | 3-2 |  | 0,316 | 0,288 | 0,062 |  | | 0,293 | | 0,289 | 0,010 |  |
| Pre-existing metabolic diagnoses | 3-2 |  | 0,414 | 0,414 | 0,001 |  | | 0,412 | | 0,415 | 0,006 |  |
| Pre-existing anemia | 3-2 |  | 0,196 | 0,166 | 0,079 |  | | 0,172 | | 0,170 | 0,006 |  |
| Pre-existing hepatic diagnoses | 3-2 |  | 0,121 | 0,115 | 0,017 |  | | 0,116 | | 0,111 | 0,015 |  |
| Pre-existing gastroenterological diagnoses | 3-2 |  | 0,347 | 0,286 | 0,131 |  | | 0,325 | | 0,281 | 0,096 |  |
| Any pre-existing ENT diagnosis | 3-2 |  | 0,301 | 0,242 | 0,134 |  | | 0,290 | | 0,273 | 0,039 |  |
| Pre-existing dysphagia | 3-2 |  | 0,062 | 0,033 | 0,138 |  | | 0,051 | | 0,040 | 0,051 |  |
| Pre-existing tracheostoma-related diagnoses | 3-2 |  | 0,026 | 0,005 | 0,176 |  | | 0,019 | | 0,006 | 0,104 |  |
| Pre-existing taste and smelling disorders | 3-2 |  | 0,002 | 0,005 | 0,044 |  | | 0,002 | | 0,004 | 0,027 |  |
| Pre-existing tinnitus | 3-2 |  | 0,029 | 0,027 | 0,012 |  | | 0,033 | | 0,034 | 0,011 |  |
| Pre-existing dermatological diagnoses | 3-2 |  | 0,271 | 0,237 | 0,079 |  | | 0,251 | | 0,243 | 0,017 |  |
| Pre-existing urogynecological diagnoses | 3-2 |  | 0,281 | 0,270 | 0,025 |  | | 0,265 | | 0,277 | 0,028 |  |
| Pre-existing ophthalmological diagnoses | 3-2 |  | 0,164 | 0,166 | 0,006 |  | | 0,170 | | 0,161 | 0,022 |  |
| Pre-existing musculoskeletal diagnoses | 3-2 |  | 0,353 | 0,288 | 0,140 |  | | 0,311 | | 0,301 | 0,023 |  |
| Pre-existing neurological diagnoses | 3-2 |  | 0,137 | 0,115 | 0,066 |  | | 0,125 | | 0,117 | 0,025 |  |
| Pre-existing Intensive Care Unit-Acquired Weakness | 3-2 |  | 0,119 | 0,115 | 0,013 |  | | 0,111 | | 0,110 | 0,002 |  |
| Pre-existing cognitive diagnoses | 3-2 |  | 0,227 | 0,156 | 0,180 |  | | 0,192 | | 0,160 | 0,083 |  |
| Pre-existing (mild) cognitive impairment | 3-2 |  | 0,090 | 0,057 | 0,127 |  | | 0,075 | | 0,060 | 0,057 |  |
| Pre-existing dementia | 3-2 |  | 0,090 | 0,074 | 0,058 |  | | 0,080 | | 0,075 | 0,016 |  |
| Pre-existing psychological diagnoses | 3-2 |  | 0,627 | 0,645 | 0,037 |  | | 0,594 | | 0,599 | 0,009 |  |
| Other pre-existing psychological diagnoses | 3-2 |  | 0,230 | 0,227 | 0,006 |  | | 0,225 | | 0,212 | 0,031 |  |
| Pre-existing depression | 3-2 |  | 0,290 | 0,303 | 0,029 |  | | 0,282 | | 0,278 | 0,010 |  |
| Pre-existing anxiety | 3-2 |  | 0,099 | 0,128 | 0,090 |  | | 0,096 | | 0,095 | 0,004 |  |
| Pre-existing trauma- and stressor-related disorders, PTSD | 3-2 |  | 0,088 | 0,095 | 0,025 |  | | 0,082 | | 0,087 | 0,015 |  |
| Pre-existing sleep disorders | 3-2 |  | 0,183 | 0,179 | 0,011 |  | | 0,177 | | 0,165 | 0,033 |  |
| Pre-existing substance use disorders | 3-2 |  | 0,304 | 0,319 | 0,032 |  | | 0,256 | | 0,256 | 0,001 |  |
| Pre-existing impairment of the general condition | 3-2 |  | 0,626 | 0,605 | 0,043 |  | | 0,629 | | 0,598 | 0,064 |  |
| Pre-existing impairment of nutrition | 3-2 |  | 0,101 | 0,065 | 0,130 |  | | 0,080 | | 0,056 | 0,089 |  |
| Pre-existing fatigue | 3-2 |  | 0,080 | 0,071 | 0,034 |  | | 0,072 | | 0,061 | 0,041 |  |
| Pre-existing (chronic) pain | 3-2 |  | 0,575 | 0,559 | 0,031 |  | | 0,588 | | 0,558 | 0,059 |  |
| Pre-existing medical diagnoses | 3-2 |  | 0,944 | 0,951 | 0,030 |  | | 0,944 | | 0,944 | 0,003 |  |
| Pre-existing venous thromboembolism | 3-2 |  | 0,036 | 0,017 | 0,114 |  | | 0,033 | | 0,020 | 0,081 |  |
| Pre-existing hair loss | 3-2 |  | 0,007 | 0,009 | 0,032 |  | | 0,006 | | 0,007 | 0,005 |  |
| Pre-existing disturbances of smell and taste | 3-2 |  | 0,002 | 0,005 | 0,044 |  | | 0,002 | | 0,004 | 0,027 |  |
| Pre-existing post-exertional malaise | 3-2 |  | 0,094 | 0,085 | 0,032 |  | | 0,086 | | 0,075 | 0,038 |  |
| Pre-existing cough | 3-2 |  | 0,051 | 0,058 | 0,035 |  | | 0,054 | | 0,058 | 0,021 |  |
| Pre-existing brain fog | 3-2 |  | 0,090 | 0,057 | 0,127 |  | | 0,075 | | 0,060 | 0,057 |  |
| Pre-existing polydipsia | 3-2 |  | 0,001 | 0,002 | 0,021 |  | | 0,001 | | 0,001 | 0,002 |  |
| Pre-existing palpitations | 3-2 |  | 0,005 | 0,006 | 0,017 |  | | 0,004 | | 0,007 | 0,029 |  |
| Pre-existing chest pain | 3-2 |  | 0,042 | 0,052 | 0,045 |  | | 0,047 | | 0,046 | 0,004 |  |
| Pre-existing fatigue | 3-2 |  | 0,003 | 0,002 | 0,035 |  | | 0,003 | | 0,001 | 0,040 |  |
| Pre-existing sexual dysfunction | 3-2 |  | 0,033 | 0,039 | 0,035 |  | | 0,035 | | 0,039 | 0,020 |  |
| Pre-existing dizziness | 3-2 |  | 0,108 | 0,084 | 0,084 |  | | 0,107 | | 0,099 | 0,029 |  |
| Pre-existing gastrointestinal disorders | 3-2 |  | 0,145 | 0,100 | 0,139 |  | | 0,130 | | 0,093 | 0,111 |  |
| Pre-existing movement disorders | 3-2 |  | 0,033 | 0,025 | 0,044 |  | | 0,031 | | 0,027 | 0,027 |  |
| Charlson: Cerebrovascular disease | 3-2 |  | 0,240 | 0,185 | 0,136 |  | | 0,220 | | 0,188 | 0,080 |  |
| Charlson: Dementia | 3-2 |  | 0,093 | 0,077 | 0,054 |  | | 0,082 | | 0,079 | 0,008 |  |
| Charlson: Peptic ulcer disease | 3-2 |  | 0,042 | 0,030 | 0,065 |  | | 0,035 | | 0,027 | 0,045 |  |
| Charlson: Renal disease | 3-2 |  | 0,284 | 0,272 | 0,028 |  | | 0,268 | | 0,273 | 0,011 |  |
| Elixhauser: Alcohol abuse | 3-2 |  | 0,129 | 0,112 | 0,051 |  | | 0,104 | | 0,097 | 0,020 |  |
| Elixhauser: Cardiac arrhythmias | 3-2 |  | 0,309 | 0,284 | 0,053 |  | | 0,282 | | 0,295 | 0,029 |  |
| Elixhauser: Chronic pulmonary disease | 3-2 |  | 0,382 | 0,513 | 0,268 |  | | 0,362 | | 0,376 | 0,028 |  |
| Elixhauser: Congestive heart failure | 3-2 |  | 0,323 | 0,322 | 0,002 |  | | 0,294 | | 0,310 | 0,034 |  |
| Elixhauser: Depression | 3-2 |  | 0,307 | 0,318 | 0,022 |  | | 0,299 | | 0,292 | 0,015 |  |
| Elixhauser: Diabetes, complicated | 3-2 |  | 0,270 | 0,246 | 0,054 |  | | 0,264 | | 0,260 | 0,010 |  |
| Elixhauser: Diabetes, uncomplicated | 3-2 |  | 0,386 | 0,373 | 0,027 |  | | 0,384 | | 0,377 | 0,015 |  |
| Elixhauser: Fluid and electrolyte disorders | 3-2 |  | 0,284 | 0,213 | 0,163 |  | | 0,238 | | 0,201 | 0,084 |  |
| Elixhauser: Hypertension, complicated | 3-2 |  | 0,198 | 0,210 | 0,029 |  | | 0,191 | | 0,207 | 0,040 |  |
| Elixhauser: Hypothyroidism | 3-2 |  | 0,163 | 0,156 | 0,019 |  | | 0,160 | | 0,148 | 0,032 |  |
| Elixhauser: Liver disease | 3-2 |  | 0,217 | 0,209 | 0,020 |  | | 0,206 | | 0,211 | 0,011 |  |
| Elixhauser: Lymphoma | 3-2 |  | 0,020 | 0,019 | 0,005 |  | | 0,018 | | 0,017 | 0,007 |  |
| Elixhauser: Obesity | 3-2 |  | 0,305 | 0,302 | 0,008 |  | | 0,312 | | 0,311 | 0,003 |  |
| Elixhauser: Other neurological disorders | 3-2 |  | 0,154 | 0,115 | 0,115 |  | | 0,130 | | 0,111 | 0,058 |  |
| Elixhauser: Paralysis | 3-2 |  | 0,105 | 0,073 | 0,113 |  | | 0,090 | | 0,075 | 0,053 |  |
| Elixhauser: Peripheral vascular disorders | 3-2 |  | 0,284 | 0,242 | 0,095 |  | | 0,256 | | 0,254 | 0,005 |  |
| Elixhauser: Psychoses | 3-2 |  | 0,053 | 0,070 | 0,068 |  | | 0,049 | | 0,059 | 0,045 |  |
| Elixhauser: Pulmonary circulation disorders | 3-2 |  | 0,079 | 0,077 | 0,007 |  | | 0,068 | | 0,067 | 0,003 |  |
| Elixhauser: Rheumatic arthritis/ collagen vascular diseases | 3-2 |  | 0,070 | 0,084 | 0,050 |  | | 0,074 | | 0,076 | 0,008 |  |
| Elixhauser: Solid tumor without metastasis | 3-2 |  | 0,170 | 0,122 | 0,137 |  | | 0,148 | | 0,117 | 0,087 |  |
| Elixhauser: Valvular disease | 3-2 |  | 0,181 | 0,177 | 0,012 |  | | 0,168 | | 0,175 | 0,018 |  |
| Elixhauser: Weight loss | 3-2 |  | 0,080 | 0,058 | 0,085 |  | | 0,064 | | 0,051 | 0,052 |  |
| No pre-existing nursing care dependency | 3-2 |  | 0,640 | 0,689 | 0,104 |  | | 0,680 | | 0,708 | 0,059 |  |
| Prior nursing care level 1 | 3-2 |  | 0,036 | 0,055 | 0,091 |  | | 0,035 | | 0,033 | 0,013 |  |
| Prior nursing care level 2 | 3-2 |  | 0,145 | 0,098 | 0,144 |  | | 0,129 | | 0,100 | 0,087 |  |
| Prior nursing care level 3 | 3-2 |  | 0,104 | 0,093 | 0,035 |  | | 0,091 | | 0,097 | 0,018 |  |
| Prior nursing care level 4 | 3-2 |  | 0,047 | 0,036 | 0,055 |  | | 0,042 | | 0,036 | 0,029 |  |
| Prior nursing care level 5 | 3-2 |  | 0,028 | 0,028 | 0,002 |  | | 0,023 | | 0,026 | 0,021 |  |

Abbr.: ENT = Ear, Nose and Throat

# **e-Table 3. Baseline characteristics of the unweighted cohort in the groups SS, IS and RS**

|  | **Percentage [95% CI]** |  |  |  |
| --- | --- | --- | --- | --- |
| **Characteristics of patients** | **SARS-CoV-2–associated sepsis**  **(n = 3964)** | **Influenza-associated sepsis (n = 689)** | **Respiratory sepsis**  **(n = 8201)** | ***P* value^a^** |
| Age, mean (SD) | 63.72 (14.51) | 65.57 (14.22) | 66.70 (14.10) | < 0.001 |
| Female gender | 38.12 [36.62-39.64] | 42.96 [39.31-46.69] | 35.54 [34.52-36.59] | < 0.001 |
| Prior employment | 33.15 [31.70-34.63] | 23.22 [20.22-26.52] | 21.23 [20.36-22.13] | < 0.001 |
| **Prior health status** |  |  |  |  |
| Dependency on nursing care (care level 1-5) | 24.37 [23.07-25.63] | 32.80 [29.41-36.14] | 39.40 [38.34-40.45] | < 0.001 |
| Inpatient nursing care | 4.64 [4.03-5.34] | 5.22 [3.80-7.15] | 7.27 [6.73-7.85] | < 0.001 |
| Vaccination against influenza | 31.61 [30.18-33.07] | 28.74 [25.48-32.23] | 33.42 [32.41-34.45] | 0.011 |
| Charlson Comorbidity Index, mean (SD) | 2.37 (2.18) | 3.01 (2.22) | 3.21 (2.32) | < 0.001 |
| Pre-existing immunosuppression incl. asplenia | 22.48 [21.20-23.80] | 29.90 [26.60-33.42] | 34.76 [33.74-35.80] | < 0.001 |
| Pre-existing medical immunosuppression | 14.68 [13.61-15.82] | 25.54 [22.43-28.93] | 21.19 [20.32-22.09] | < 0.001 |
| Pre-existing asplenia | 0.18 [0.09-0.36] | 0.00 [0.00-0.55] | 0.45 [0.33-0.62] | 0.017 |
| Prior organ transplantation | 1.01 [0.74-1.37] | 1.45 [0.79-2.65] | 1.22 [1.00-1.48] | 0.464 |
| Pre-existing infection/colonisation with multi-resistant pathogens | 1.51 [1.18-1.94] | 2.61 [1.66-4.09] | 4.43 [4.00-4.89] | < 0.001 |
| Previously implanted foreign material | 19.12 [17.93-20.38] | 20.03 [17.21-23.18] | 28.46 [27.49-29.45] | < 0.001 |
| Pre-existing mechanical ventilation | 3.20 [2.70-3.80] | 10.16 [8.12-12.64] | 11.07 [10.41-11.77] | < 0.001 |
| Pre-existing long-term mechanical ventilation | 1.16 [0.87-1.54] | 4.06 [2.83-5.81] | 3.19 [2.84-3.60] | < 0.001 |
| Pre-existing dialysis | 2.80 [2.33-3.36] | 4.35 [3.07-6.15] | 3.22 [2.86-3.62] | 0.082 |
| Pre-existing immobility | 14.08 [13.03-15.19] | 19.30 [16.53-22.42] | 25.17 [24.24-26.12] | < 0.001 |
| Prior palliative treatment | 1.56 [1.22-2.00] | 2.03 [1.21-3.38] | 3.08 [2.73-3.48] | < 0.001 |
| **Characteristics of the index hospital stay** |  |  |  |  |
| Septic shock | 4.26 [3.68-4.94] | 8.13 [6.31-10.41] | 26.61 [25.66-27.57] | < 0.001 |
| Hospital length of stay (incl. transfer chains), mean (SD) | 28.74 (25.62) | 30.14 (28.14) | 48.67 (40.28) | < 0.001 |
| Discharge disposition of sepsis hospital survivors (incl. transfer chains)  Regular discharge  Rehabilitation  Nursing home  Hospice  Other | 84.61 [83.55-85.68]  7.95 [6.89-9.01]  4.39 [3.33-5.45]  0.03 [0.00-1.09]  3.03 [1.97-4.09] | 76.92 [74.02-80.01]  10.60 [7.69-13.68]  7.98 [5.08-11.07]  0.00 [0.00-3.08]  4.50 [1.60-7.58] | 68.50 [67.52-69.51]  14.14 [13.16-15.15]  12.01 [11.02-13.02]  0.39 [0.00-1.40]  4.95 [3.96-5.96] | < 0.001 |
| **Focus of infection** |  |  |  |  |
| Upper respiratory tract | 1.84 [1.47-2.31] | 0.87 [0.40-1.89] | 2.17 [1.88-2.51] | 0.045 |
| Lower respiratory tract | 90.87 [89.93-91.73] | 83.02 [80.03-85.64] | 98.94 [98.69-99.14] | < 0.001 |
| Lower respiratory tract, spec. bronchi | 5.85 [5.16-6.63] | 25.54 [22.43-28.93] | 15.11 [14.35-15.90] | < 0.001 |
| Lower respiratory tract, spec. lung | 89.51 [88.51-90.42] | 73.44 [70.02-76.60] | 91.14 [90.50-91.73] | < 0.001 |
| Respiratory tract, non-specific | 0.86 [0.61-1.20] | 40.93 [37.32-44.64] | 0.84 [0.67-1.06] | < 0.001 |
| Genitourinary system | 21.72 [20.46-23.03] | 20.61 [17.76-23.79] | 36.01 [34.98-37.05] | < 0.001 |
| Wound and soft tissue infection | 3.63 [3.09-4.26] | 5.37 [3.92-7.31] | 11.90 [11.22-12.62] | < 0.001 |
| Intraabdominal and retroperitoneal | 3.00 [2.51-3.58] | 3.92 [2.71-5.64] | 17.49 [16.68-18.32] | < 0.001 |
| Intrathoracic | 1.19 [0.89-1.57] | 1.45 [0.79-2.65] | 4.39 [3.97-4.85] | < 0.001 |
| Central nervous system | 0.45 [0.29-0.72] | 0.73 [0.31-1.69] | 2.05 [1.76-2.38] | < 0.001 |
| Bloodstream and vascular infection | 5.10 [4.45-5.82] | 5.52 [4.04-7.48] | 12.60 [11.90-13.33] | < 0.001 |
| Bones, cartilage, joints | 0.45 [0.29-0.72] | 1.74 [1.00-3.02] | 3.58 [3.20-4.01] | < 0.001 |
| Gastrointestinal tract and diarrhea | 8.55 [7.72-9.46] | 9.43 [7.47-11.85] | 11.45 [10.78-12.16] | < 0.001 |
| Systemic viral infection | 100.00 [99.90-100.00] | 2.47 [1.55-3.92] | 3.21 [2.85-3.61] | < 0.001 |
| Infections of the genital tract and sexually transmitted diseases | 0.68 [0.47-0.99] | 1.02 [0.49-2.08] | 2.37 [2.06-2.72] | < 0.001 |
| Non-specific | 57.24 [55.69-58.77] | 40.35 [36.75-44.06] | 71.74 [70.75-72.70] | < 0.001 |
| Device-related infection | 4.16 [3.58-4.83] | 4.93 [3.55-6.82] | 11.13 [10.47-11.83] | < 0.001 |
| Nosocomial infection, pneumonia | 10.57 [9.65-11.57] | 18.29 [15.58-21.35] | 41.54 [40.48-42.61] | < 0.001 |
| **Organ dysfunction** |  |  |  |  |
| Respiratory dysfunction | 94.22 [93.45-94.91] | 93.32 [91.21-94.96] | 90.61 [89.96-91.22] | < 0.001 |
| Coagulopathy | 9.11 [8.25-10.04] | 12.48 [10.22-15.16] | 28.40 [27.43-29.38] | < 0.001 |
| Cardiovascular dysfunction | 22.10 [20.83-23.42] | 29.75 [26.46-33.27] | 49.21 [48.13-50.30] | < 0.001 |
| Hepatic dysfunction | 1.11 [0.83-1.49] | 1.74 [1.00-3.02] | 3.87 [3.47-4.30] | < 0.001 |
| Renal dysfunction | 16.40 [15.28-17.58] | 25.69 [22.57-29.08] | 41.10 [40.04-42.17] | < 0.001 |
| Central nervous system dysfunction | 20.59 [19.36-21.87] | 29.17 [25.90-32.67] | 41.34 [40.27-42.41] | < 0.001 |
| **Therapies received during index hospital stay** |  |  |  |  |
| Dialysis | 6.96 [6.21-7.80] | 11.90 [9.69-14.53] | 17.85 [17.04-18.70] | < 0.001 |
| Extracorporeal membrane oxygenation (ECMO) | 0.98 [0.72-1.34] | 1.60 [0.89-2.84] | 1.67 [1.41-1.97] | 0.012 |
| Mechanical ventilation | 57.52 [55.97-59.05] | 66.62 [63.01-70.04] | 67.66 [66.64-68.67] | < 0.001 |
| Extracorporeal liver replacement therapy | 0.05 [0.01-0.18] | 0.00 [0.00-0.55] | 0.01 [0.00-0.07] | 0.342 |
| Surgical treatment | 22.12 [20.86-23.44] | 30.77 [27.44-34.31] | 61.79 [60.73-62.83] | < 0.001 |
| Tracheostomy | 10.49 [9.58-11.49] | 13.21 [10.88-15.94] | 23.22 [22.32-24.14] | < 0.001 |

Abbr.: n = total number; SD = standard deviation; CI = confidence interval

^a^ *P* value of the *χ*^2^-Test of the Null hypothesis that the three groups do not differ in the distribution of the characteristics (categorical variables), or the *P* value of the *F* Test of the Null hypothesis that the three groups have equal population means (metric variables).

# **e-Table 4. Pairwise risk differences (RD), mean differences (MD), risk ratios (RR) and standardized mean differences (SMD) of selected outcomes within 3 to 12 months from hospital discharge among N = 11396 3-month survivors**

| **Outcome** | **Percentage [95% CI]** | | **RD or MD, % [95% CI]** | **RR [95% CI]** | **SMD** | ***P* value** |
| --- | --- | --- | --- | --- | --- | --- |
| **Respiratory sepsis vs. SARS-CoV-2–associated sepsis** | **Respiratory sepsis** | **SARS-CoV-2–associated sepsis** |  |  |  |  |
| Death | 14.94 [14.10-15.82] | 7.88 [6.43-9.63] | 7.06 [5.25-8.87] | 1.90 [1.54-2.34] | - | < 0.001 |
| At least one medical, psychological or cognitive diagnosis | 96.91 [96.47-97.30] | 97.00 [96.13-97.69] | -0.09 [-0.97-0.78] | 1.00 [0.99-1.01] | - | 0.836 |
| Number of affected domains, mean (SD) | 1.86 (0.76) | 1.76 (0.74) | 0.10 [0.06-0.13] | - | 0.13 | < 0.001 |
| Medical diagnosis | 96.13 [95.64-96.57] | 96.42 [95.51-97.15] | -0.28 [-1,22-0.65] | 1.00 [0.99-1.01] | - | 0.560 |
| Cognitive diagnosis | 28.06 [26.95-29.21] | 21.45 [19.63-23.40] | 6.61 [4.41-8.81] | 1.31 [1.19-1.44] | - | < 0.001 |
| Psychological diagnosis | 61.69 [60.42-62.95] | 58.29 [56.21-60.35] | 3.40 [0.97-5.83] | 1.06 [1.02-1.10] | - | 0.006 |
| Number of outpatient treatments, mean (SD) | 7.07 (4.41) | 7.57 (4.55) | -0.50 [-0.72-(-0.29)] | - | - 0.11 | < 0.001 |
| Number of rehospitalizations, mean (SD) | 1.18 (1.68) | 0.82 (1.57) | 0.35 [0.27-0.44] | - | 0.21 | < 0.001 |
| **Respiratory sepsis vs. Influenza-associated sepsis** | **Respiratory sepsis** | **Influenza-associated sepsis** |  |  |  |  |
| Death | 14.94 [14.10-15.82] | 10.78 [8.08-14.23] | 4.16 [0.99-7.33] | 1.39 [1.04-1.85] | - | 0.024 |
| At least one medical, psychological or cognitive diagnosis | 96.91 [96.47-97.30] | 97.80 [96.05-98.79] | -0.89 [-2.25-0.47] | 0.99 [0.98-1.00] | - | 0.268 |
| Number of affected domain, mean (SD) | 1.86 (0.76) | 1.83 (0.76) | 0.03 [-0.05-0.10] | - | 0.04 | 0.469 |
| Medical diagnosis | 96.13 [95.64-96.57] | 96.98 [94.99-98.19] | -0,84 [-2,45-0.77] | 0.99 [0.98-1.01] | - | 0.355 |
| Cognitive diagnosis | 28.06 [26.95-29.21] | 25.30 [21.64-29.34] | 2.76 [-1.25-6.77] | 1.11 [0.95-1.30] | - | 0.189 |
| Psychological diagnosis | 61.69 [60.42-62.95] | 60.96 [56.43-65.30] | 0.74 [-3.88-5.35] | 1.01 [0.94-1.09] | - | 0.754 |
| Number of outpatient treatments, mean (SD) | 7.07 (4.41) | 6.81 (4.19) | 0.26 [-0.13-0.65] | - | 0.06 | 0.188 |
| Number of rehospitalizations, mean (SD) | 1.18 (1.68) | 1.10 (1.75) | 0.08 [-0.08-0.23] | - | 0.05 | 0.315 |
| **Influenza-associated sepsis vs. SARS-CoV-2–associated sepsis** | **Influenza-associated sepsis** | **SARS-CoV-2–associated sepsis** |  |  |  |  |
| Death | 10.78 [8.08-14.23] | 7.88 [6.43-9.63] | 2.90 [-0.54-6.34] | 1.37 [0.97-1.94] | - | 0.080 |
| At least one medical, psychological or cognitive diagnosis | 97.80 [96.05-98.79] | 97.00 [96.13-97.69] | 0.80 [-0.71-2.31] | 1.01 [0.99-1.02] | - | 0.345 |
| Number of affected domains, mean (SD) | 1.83 (0.76) | 1.76 (0.74) | 0.07 [-0.01-0.15] | - | 0.09 | 0.070 |
| Medical diagnosis | 96.98 [94.99-98.19] | 96.42 [95.51-97.15] | 0.56 [-1.18-2,30] | 1.01 [0.99-1.02] | - | 0.551 |
| Cognitive diagnosis | 25.30 [21.64-29.34] | 21.45 [19.63-23.40] | 3.85 [-0.44-8.13] | 1.18 [0.99-1.41] | - | 0.069 |
| Psychological diagnosis | 60.96 [56.43-65.30] | 58.29 [56.21-60.35] | 2.66 [-2.24-7.56] | 1.05 [0.96-1.13] | - | 0.290 |
| Number of outpatient treatments, mean (SD) | 6.81 (4.19) | 7.57 (4.55) | -0.76 [-1.18-(-0.35)] | - | - 0.17 | < 0.001 |
| Number of rehospitalizations, mean (SD) | 1.10 (1.75) | 0.82 (1.57) | 0.27 [0.11-0.44] | - | 0.16 | 0.001 |

Abbr.: CI = confidence interval; RD = Risk difference; MD = mean difference; RR = risk ratio; SMD = standardized mean difference

# **e-References**

1. Thaweethai T, Jolley SE, Karlson EW, et al. Development of a Definition of Postacute Sequelae of SARS-CoV-2 Infection. *Jama*. Jun 13 2023;329(22):1934-1946. doi:10.1001/jama.2023.8823

2. Quan H, Sundararajan V, Halfon P, et al. Coding Algorithms for Defining Comorbidities in ICD-9-CM and ICD-10 Administrative Data. *Medical Care*. 2005;43(11):1130-1139. doi:10.1097/01.mlr.0000182534.19832.83

3. Wedekind L, Fleischmann-Struzek C, Rose N, et al. Development and validation of risk-adjusted quality indicators for the long-term outcome of acute sepsis care in German hospitals based on health claims data. *Front Med (Lausanne)*. 2022;9:1069042. doi:10.3389/fmed.2022.1069042
